# Cholic Acid-Based Antimicrobial Peptide Mimics as Antibacterial Agents

**DOI:** 10.3390/ijms23094623

**Published:** 2022-04-21

**Authors:** Jie Wu, Tsz Tin Yu, Rajesh Kuppusamy, Md. Musfizur Hassan, Amani Alghalayini, Charles G. Cranfield, Mark D. P. Willcox, David StC. Black, Naresh Kumar

**Affiliations:** 1 School of Chemistry, The University of New South Wales, Sydney, NSW 2052, Australia; jie.wu1@unsw.edu.au (J.W.); tsztin.yu@unsw.edu.au (T.T.Y.); r.kuppusamy@unsw.edu.au (R.K.); md.m.hassan@unsw.edu.au (M.M.H.); 2 School of Optometry and Vision Science, University of New South Wales, Sydney, NSW 2052, Australia; m.willcox@unsw.edu.au; 3 School of Life Sciences, University of Technology Sydney, P.O. Box 123, Ultimo, NSW 2007, Australia; amani.alghalayini@student.uts.edu.au (A.A.); charles.cranfield@uts.edu.au (C.G.C.)

**Keywords:** cholic acid, antimicrobial peptide, peptidomimetics, antibacterial, membrane disruption

## Abstract

There is a significant and urgent need for the development of novel antibacterial agents to tackle the increasing incidence of antibiotic resistance. Cholic acid-based small molecular antimicrobial peptide mimics are reported as potential new leads to treat bacterial infection. Here, we describe the design, synthesis and biological evaluation of cholic acid-based small molecular antimicrobial peptide mimics. The synthesis of cholic acid analogues involves the attachment of a hydrophobic moiety at the carboxyl terminal of the cholic acid scaffold, followed by the installation of one to three amino acid residues on the hydroxyl groups present on the cholic acid scaffold. Structure–activity relationship studies suggest that the tryptophan moiety is important for high antibacterial activity. Moreover, a minimum of +2 charge is also important for antimicrobial activity. In particular, analogues containing lysine-like residues showed the highest antibacterial potency against Gram-positive *S. aureus*. All di-substituted analogues possess high antimicrobial activity against both Gram-positive *S. aureus* as well as Gram-negative *E. coli* and *P. aeruginosa*. Analogues **17c** and **17d** with a combination of these features were found to be the most potent in this study. These compounds were able to depolarise the bacterial membrane, suggesting that they are potential antimicrobial pore forming agents.

## 1. Introduction

Treatment of bacterial infections is one of the most prevalent and challenging health issues faced today [1]. The evolutionary stress on a bacterial population exerted by the use of conventional antimicrobial agents induces antibiotics resistance [2]. Nowadays, infections caused by multi-drug resistant pathogens account for one of the leading causes of hospitalisation and increased mortality worldwide [3,4]. A team of European researchers estimated that over 33,000 people in Europe die each year as a direct consequence of an infection due to bacteria that are resistant to antibiotics [5]. A review commissioned by the government of the United Kingdom in 2014 estimated that the total of about 700,000 deaths occurring globally every year from drug-resistant bacterial infections could rise to 10 million each year by 2050 if corrective action is not taken [3].

Naturally occurring antimicrobial peptides (AMPs) have long been found to be ubiquitous and play a central role in the first line of defence in the innate immune system in all classes of organisms, ranging from prokaryotes to higher eukaryotes [6]. AMPs possess a broad spectrum activity against bacteria, viruses, parasites and fungi [6]. These AMPs adopt a wide range of secondary structures and non-conserved sequence homology, suggesting that antimicrobial activity is not dependent on any conserved amino acid sequences nor specific protein folding [7,8]. Instead, their facially amphiphilic character with hydrophobic residues clustered on one face of the helix and the cationic groups oriented on the opposite face appears to be essential for their antimicrobial properties [9]. As a result of this distinct structural property of AMPs, it is believed that AMPs act directly on the bacterial cell membrane through an initial electrostatic interaction between the cationic residues and the negatively charged cell membrane, followed by the membrane insertion via hydrophobic interactions between the hydrophobic residues and the fatty acid chains of the phospholipids, disrupting the bacterial cell membrane permeability.

Despite their desirable characteristics, AMPs have several drawbacks. Their relatively large size (ca. 20–50 amino acids) aggravates structural complexity and makes AMPs relatively difficult and expensive to synthesise on a large scale [8]. Another major challenge in the development of AMPs for clinical use is the lack of metabolic stability due to their susceptibility to proteolytic degradation [8,10]. Consequently, interest in antimicrobial strategies has shifted from AMPs to small molecular antimicrobial peptidomimetics (SMAMPs), which are non-peptidic small molecules that adopt the same mechanism of action as AMPs while circumventing the problems associated with high manufacturing costs and stability.

**Figure 1:**
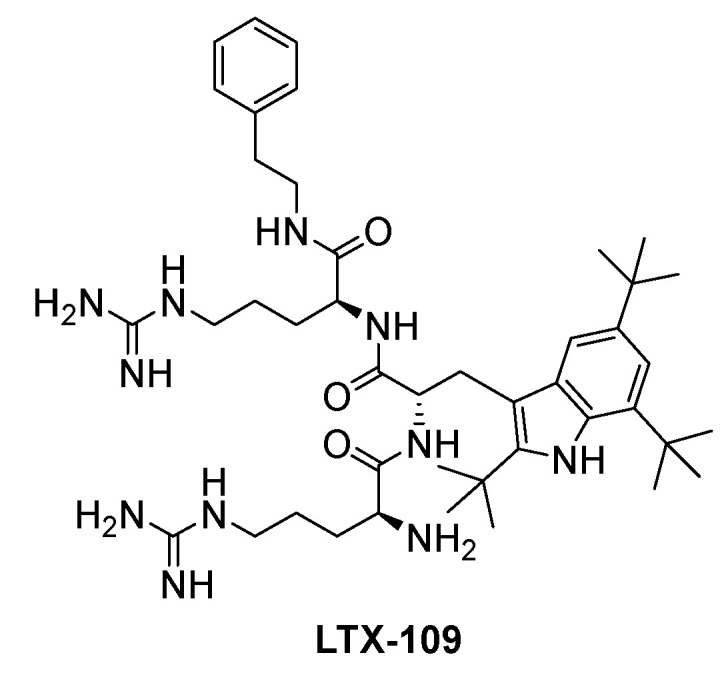
Structure of LTX-109.

Many synthetic SMAMPs have been investigated using a variety of scaffolds to achieve structural amphiphilicity with several currently undergoing clinical trials [11,12]. For example, LTX-109 (Figure 1), a tripeptide composed of a modified tryptophan connected to guanidine and arginine with the end capped with a hydrophobic phenethylamino group, recently completed clinical phase II clinical trials for the treatment of nasal decolonisation of methicillin-resistant and -sensitive *Staphylococcus aureus* strains [13]. Given their improved properties over natural AMPs and low resistance potential, SMAMPs are a promising class for the further development of therapeutic antibacterial agents.

**Figure 2:**
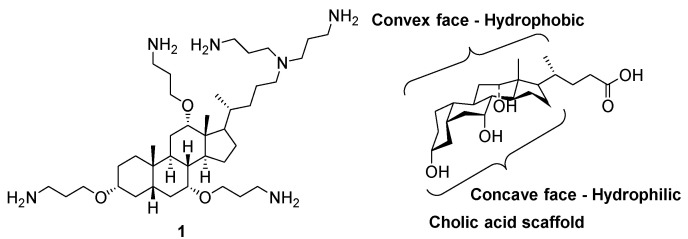
Structure of the synthetic SMAMP **1** and the facial amphiphilicity of the cholic-acid scaffold.

Previously, the Savage group introduced synthetic SMAMPs (e.g., **1**) consisting of a sterol backbone appended with different cationic groups, which showed broad-spectrum activity against multi-drug resistant strains of Gram-positive and Gram-negative bacteria [14,15]. The facial amphiphilicity of the cholic-acid scaffold is attributed to the presence of hydroxyl groups at the concave face and the presence of the hydrocarbons at the convex face (Figure 2) [8]. In addition, the four fused rings provide a hydrophobic core that is large enough to allow spatial segregation between the hydrophobic and hydrophilic groups, further conferring the amphiphilic nature of the scaffold [10].

**Figure 3:**
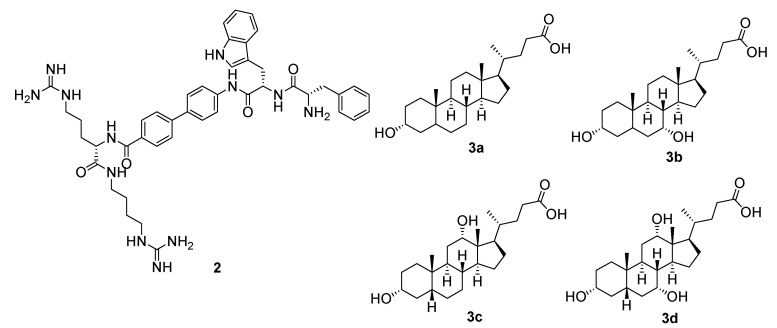
Structures of biphenyl-based peptidomimetic **2** and cholic acid scaffolds **3**.

Many studies have highlighted the prevalence of tryptophan in potent naturally occurring AMPs, such as indolicidin and tritrpticin, and its importance in hydrophobic lipid binding has been widely recognised [16]. Our research group has also recently developed a potent, selective biphenyl-based peptidomimetic **2** (Figure 3), which incorporates the hydrophobic tryptophan motif to act as an antibacterial agent against both Gram-positive *S. aureus* and Gram-negative *Escherichia coli* [17]. Based upon the observations made in these studies, it was envisaged that the cholic acid scaffolds (lithocholic acid (LICA) **3a**, chenodeoxycholic acid (CHCA) **3b**, deoxycholic acid (DCA) **3c** and cholic acid (CA) **3d**) could be used to generate SMAMPs by incorporating a tryptophan moiety at the carboxyl terminal and cationic amino acid residues at the hydroxyl groups of the molecule. These SMAMPs would allow us to further elucidate the role of tryptophan in membrane permeability and to explore the impact that total net charge has on biological activity.

## 2. Results and Discussion

### 2.1. Synthesis of Cholic Acid-Based Antimicrobial Peptide Mimics

**Scheme 1:**
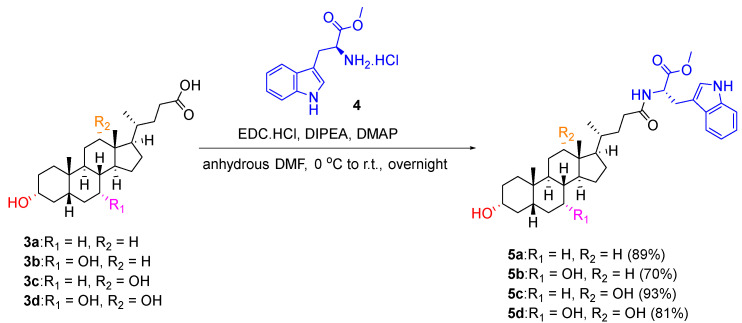
Synthesis of tryptophan methyl ester substituted cholic acid derivatives **5a–5d**.

**Scheme 2:**
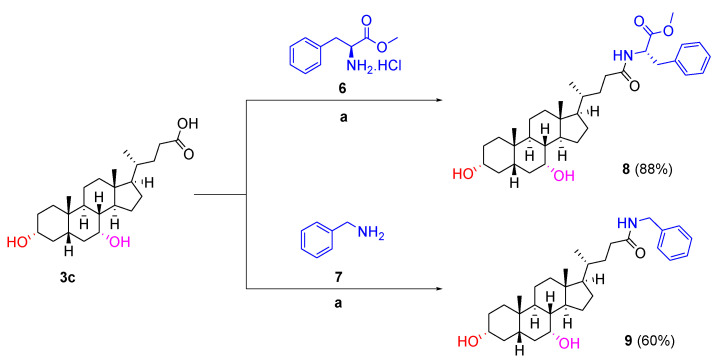
Synthesis of **8** and **9**. Reaction condition: (a) EDC.HCl, DIPEA, DMAP, anhydrous DMF, 0 °C to rt, overnight.

The cholic acid scaffolds (lithocholic acid **3a**, chenodeoxycholic acid **3b**, deoxycholic acid **3c**, cholic acid **3d**) (Figure 3) that varied in the number and position of the hydroxyl groups were chosen as starting materials to generate a library of facially amphiphilic SMAMPs by incorporating a hydrophobic moiety at the carboxyl terminal and cationic amino acid residues at the hydroxyl groups of the starting scaffolds. To achieve this, the carboxyl terminus of the cholic acid derivatives **3a–3d** were firstly functionalised with tryptophan methyl ester via *N*-(3-dimethylaminopropyl)-*N′*-ethylcarbodiimide (EDC) coupling using the method reported by Gomez-Mendoza et al. in 70–93% yield (Scheme 1) [18]. Similarly, phenylalanine methyl ester conjugated chenodeoxycholic acid derivative **8** was synthesised from chenodeoxycholic acid **3c** and L-phenylalanine methyl ester hydrochloride **6** in 88% yield (Scheme 2). The aromatic benzylamine **7** was also conjugated to chenodeoxycholic acid **3c** using similar reaction conditions to result in the benzylamine derivative **9** in 60% yield (Scheme 2).

**Scheme 3:**
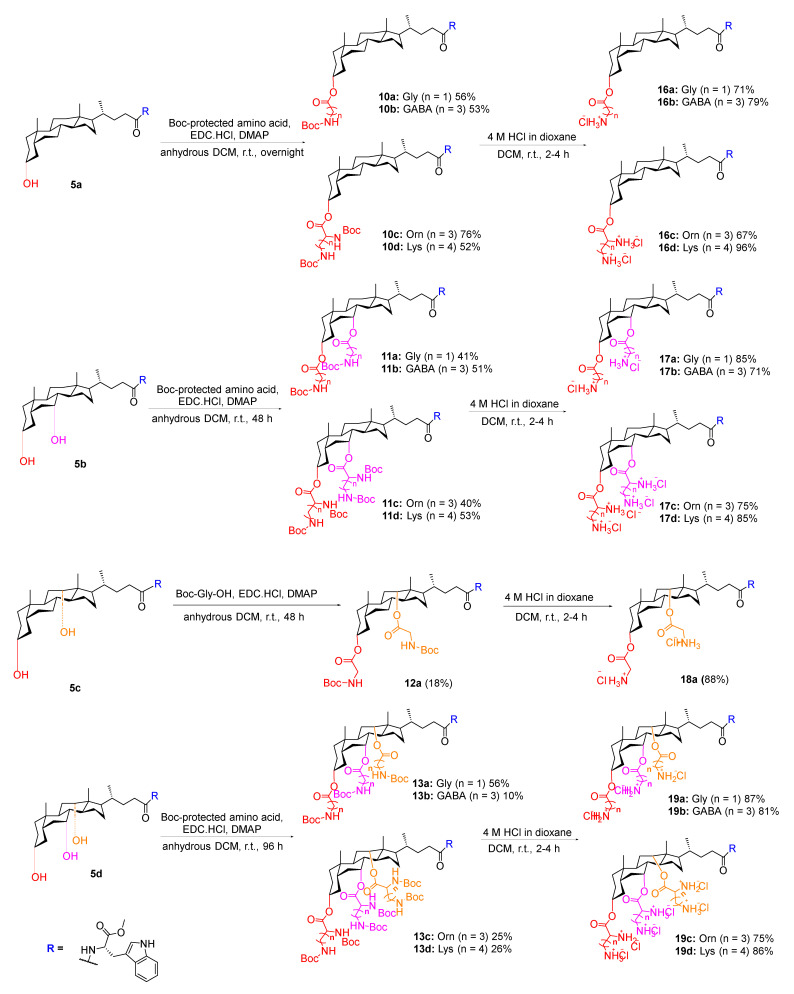

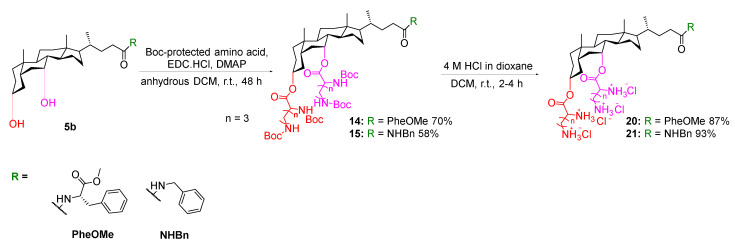
Synthesis of the desired mono-, di- and tri- substituted cholic acid based SMAMPs. Amino acids are **a**: Gly, **b**: GABA, **c**: Orn and **d**: Lys.

Following the successful coupling of the hydrophobic group to the carboxyl terminal of the cholic acid scaffolds, the cationic amino groups were tethered to the hydroxyl termini. This was achieved via ester bond formation using Steglich esterification conditions, following the procedure described by Yadav et al. (Scheme 3) [19]. Amino acids such as glycine (Gly), *γ*-aminobutyric acid (GABA), ornithine (Orn) or lysine (Lys) were chosen to provide the charged characteristic in order to investigate the effect of total net charge on antibacterial activity. Moreover, the number of ester bonds installed was dependent on the number of hydroxyl groups present on the starting scaffold. Thus, the Boc-protected mono-, di- and tri- amino derived cholic acid intermediates were firstly synthesised in 10–76% yield. Further treatment with 4 M hydrogen chloride/dioxane solution in dichloromethane at room temperature for 2–4 h resulted in the formation of desired mono-, di- and tri- substituted cholic acid based SMAMPs in 67–96% yield (Scheme 3). The variable yield of the SMAMPs product could be due to the loss of product when washing the crude product with diethyl ether. The purity of the SMAMPs was estimated from NMR spectra and was judged to be >95% (see Supplementary Materials File S1).

**Scheme 4:**
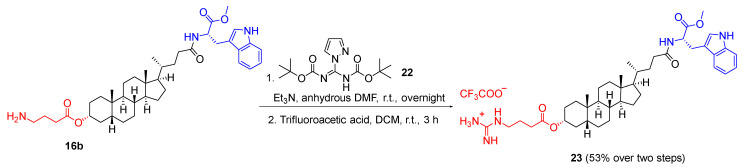
Synthesis of analogue **23** bearing a terminal guanidine group.

To investigate the effect of specific amino functionality, a terminal guanidine was prepared from deprotected GABA monoester **16b** and *N*,*N*′-di-Boc-1*H*-pyrazole-1-carboxamidine **22** in the presence of basic triethylamine at room temperature (Scheme 4), followed by the removal of Boc protecting groups with trifluoroacetic acid (TFA) to give analogue **23** in a yield of 53% over two steps, following the method reported by Yu et al. [20,21].

In this study, two dipeptide cholic acid analogues, **24** and **26**, were also synthesised to investigate the effect of chain length and functionality of the cationic residues on the antibacterial activity. In particular, analogue **26** was designed in accordance with the previously reported most active glycine-valine derivative by Yadav et al. [19]. Dipeptide derivatives were synthesised by two different reaction pathways, namely amine-reactive crosslinker reactive groups, or carboxyl-reactive chemical groups.

**Scheme 5:**
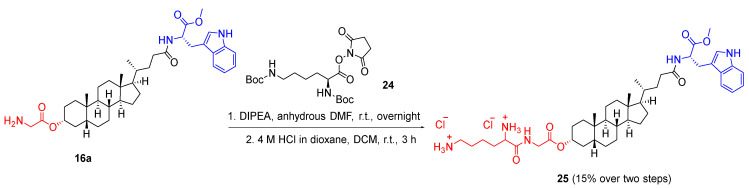
Synthesis of glycine–lysine dipeptide analogues **25**.

For the synthesis of analogue **25**, the glycine terminal of the deprotected lithocholic acid derivative **16a** was conjugated with a lysine residue. This was achieved by treating analogue **16a** with *N_ε_*-Z-*N_α_*-Boc-L-lysine hydroxysuccinimide ester **24** in the presence of DIPEA in room temperature overnight to afford the *N*-Boc-protected glycine–lysine dipeptide analogues. The Boc-protected product was then deprotected using 4 M hydrogen chloride/dioxane solution to afford the dipeptide analogue **25** in a yield of 15% over two steps (Scheme 5).

**Scheme 6:**
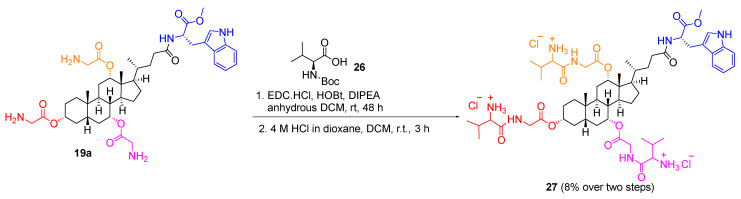
Synthesis of glycine–valine dipeptide analogues **27**.

Conversely, a glycine–valine dipeptide analogue **27** was synthesised via EDC coupling following the procedure reported by Yadav et al. [19] *N*-Boc-protected valine **26** was stirred with glycine triester derivative **19a**, EDC.HCl, 1-hydroxybenzotriazole (HOBt) hydrate and DIPEA in anhydrous dichloromethane at room temperature for 48 h to yield the Boc-protected dipeptide cholic acid derivative. The deprotection of the Boc groups was performed using 4 M HCl in dioxane to afford the Gly–Val dipeptide analogue **27** in a yield of 8% over two steps (Scheme 6).

### 2.2. Structure–Activity Relationship Study

The antibacterial activity of each compound was measured as the minimum inhibitory concentration (MIC, i.e., the lowest concentration of the compound needed to inhibit the growth of bacterial cells) following a previously published protocol [22]. The compounds were tested in a concentration range between 1 and 125 μM against Gram-positive *S. aureus* as well as Gram-negative *E. coli* and *Pseudomonas aeruginosa*. MIC values for tested compounds ranged from 4 μM to greater than 125 μM against *S. aureus* (Table 1). The most active compounds against the Gram-positive *S. aureus* SA38 strain were **16d**, **17c** and **17d**, sharing a MIC value of 4 μM. A selection of compounds that had high antibacterial activity (with MIC values of 4 or 8 μM) against the Gram-positive *S*. *aureus* SA38 strain were also tested against antibiotic-resistant *S*. *aureus* SA111 and SA112 strains [23]. These compounds showed similar MIC values of 4 or 8 μM against the antibiotic-resistant *S*. *aureus* SA111 and SA112 strains compared to *S*. *aureus* SA38 strain. Across all three tested *S. aureus* strains, compounds **17c** and **17d** were the most active compounds, with MIC value of 4 μM against all three *S. aureus* strains.

Against Gram-negative *E. coli* and *P. aeruginosa*, the tested compounds generally showed lower antibacterial activities with higher MIC values compared to Gram-positive *S. aureus*, except compounds **17b**, **19c** and **27**, which had lower MIC values of 4, 4 and 8 μM, respectively, against *E. coli* and were the most active compounds against *E. coli*. On the other hand, compounds **17c** and **17d** were the most active compounds against *P. aeruginosa*, sharing a MIC value of 8 μM.

#### 2.2.1. Effect of Total Net Charge

Positively charged groups are one of the most important features for non-receptor mediated membrane antimicrobial agents, due to the subtle difference in membrane composition between prokaryotic and eukaryotic cell membranes [6,24]. A number of studies had suggested that the number of total net charge played a crucial role in describing the antimicrobial activities of AMPs and their peptidomimetics against bacterial membranes [24]. Many reported higher positive charges were correlated with enhanced bactericidal effect [25,26]. In this study, analogues where the number of positively charged residues varied from 1 to 3 were synthesised, and the number of net charges of these analogues varied between +1 and +6. Against Gram-positive *S. aureus* SA38 strain, increasing the net charge of analogues from +1 to +2 resulted in greater than 32-fold increase in antimicrobial activity (i.e., **16a** (>250 μM) to **17a** (8 μM)). Meanwhile, the activity remained largely unchanged when the net charge of the analogues was further increased from +2 to +4, with a slight increase in activity observed between **16c** and **17c**, while no difference was observed between **16d** and **17d** against Gram-positive *S. aureus* SA38 strain. Analogues bearing three cationic residues (with net charge of +3 or +6) resulted in either equal (e.g., **17b** and **19b**) or a four-fold reduction in antimicrobial activity (e.g., **17a** and **19a**) against Gram-positive *S. aureus* SA38 strain. This trend was also observed in the antibacterial activities of compounds against Gram-negative *P. aeruginosa*, as compounds **17c** and **17d** with the optimum net charge of +4 shard a MIC value of 8 μM against Gram-negative *P. aeruginosa* and were the most potent compounds against Gram-negative *P. aeruginosa*. Compounds with a net charge of above or below the optimum value of +4 showed lower antibacterial activity against Gram-negative *P. aeruginosa*. Overall, the di-substituted cholic acid derivatives **17c** and **17d**, each bearing two cationic amino acid groups with an overall net charge of +4, were optimal for antibacterial activity.

#### 2.2.2. Effect of the Tryptophan

In this study, the tryptophan derivative at the carboxyl terminal was modified to further elucidate the significance of tryptophan in antimicrobial activity. Replacement of tryptophan methyl ester with a phenylalanine methyl ester (**20**) or a benzylamine (**21**) both resulted in a two-fold decrease in antimicrobial activity against all tested Gram-positive *S. aureus* strains compared to tryptophan-containing derivative **17c**, as suggested by an increase in MIC value from 4 μM for compound **17c** to 8 μM for compounds **20** and **21**. A similar trend was also observed against Gram-negative *P. aeruginosa*, as tryptophan-containing compound **17c** with MIC value of 8 μM was about four times more active than phenylalanine-containing compound **20** and benzylamine **21**, which had MIC value of 31 μM against *P. aeruginosa*. However, activity against Gram-negative *E. coli* was maintained after the replacement of the tryptophan methyl ester residue (**17c)**. This result indicated that the tryptophan residue is not essential for activity against Gram-negative *E. coli*. Indeed, analogue **27** bearing a tryptophan methyl ester and a previously reported literature compound with a benzyl residue at the carboxyl terminal of cholic acid in place of the tryptophan methyl ester moiety showed the same antibacterial activity of 8 μM against Gram-negative *E. coli* [19]. While tryptophan, phenylalanine and benzylamine are all hydrophobic and aromatic, tryptophan constitutes a unique biochemistry with characteristic indole functionality. The hydrophobic aromatic ring has a strong tendency to facilitate the insertion of the molecule into the biological membrane, however, the presence of an indole side chain allows hydrogen bonding, also giving a hydrophilic character. Hence, tryptophan containing amphiphiles has a stronger preference for the membrane-water interfacial regions of lipid bilayers [16,27]. Theoretically, non-tryptophan counterparts may promote deeper penetration into the membrane due to their high hydrophobicity [28]. However, this trend was not aligned with the actual MICs values, suggesting that the formation of membrane-spanning pores is unlikely. The disparity in activities between the Gram-negative and Gram-positive bacterial strains could be explained by the presence of the lipopolysaccharide outer cell membrane external to the peptidoglycan cell wall present only in Gram-negative bacteria, suggesting a different mode of action between Gram-positive and Gram-negative strains. Gram-negative bacteria have a stronger efflux system that recognise noxious compounds and act as a transporter to excrete compounds from within the bacterial cell [29].

#### 2.2.3. Presence of Specific Amino Acid Residues

Despite the significant role played by hydrophobic tryptophan amino acids in membrane permeability, cholic acid scaffold encompassing cationic amino acid residues with different length and charges were also synthesised to investigate the role of specific amino acids in relation to antibacterial activity. Analogues **16c, 16d** and **23** containing ornithine, lysine or guanidine were identified to be the active compounds in the mono-substituted lithocholic acid-based scaffold against Gram-positive *S. aureus* SA38 strain. Among these analogues, the guanidine bearing compound **23** was least active, with the highest MIC value of 16 μM, and the lysine substituted analogue **16d** was the most active, with the lowest MIC value of 4 μM. Ornithine is a non-proteinogenic cationic amino acid that retains the primary amino groups of lysine, but it is one carbon shorter than lysine. However, replacement of lysine (**16d**) with ornithine (**16c**) resulted in a two-fold reduction in activity. The two-fold reduction in activity was also observed in the replacement of lysine with a dipeptide variant containing glycine and lysine residues (**25**). These findings, therefore, suggested that while cationic charges are essential for antibacterial activity, deviation from the optimum cationic chain length and specific hydrophobicity window would result in the reduction in antimicrobial activity.

#### 2.2.4. Effect of the Position and Number of the Cationic Groups

In this study, analogues **17a** and **18a** were synthesised to investigate whether a change in the position of the cationic groups on the cholic acid scaffold would influence their antimicrobial activities. Analogues **17a** and **18a** differ in the sites of cationic glycine amino side chains, with the second glycine side chains attached at either C7-OH or C12-OH of the tryptophan conjugated cholic acid scaffold. Equal activities of 8, 16 and >125 μM against all tested Gram-positive *S. aureus* strains, Gram-negative *E. coli* and *P. aeruginosa* for both analogues suggested that the antimicrobial mechanism was not dependent on the position of the substituted amine for the di-substituted cholic acid-based antimicrobial agents. However, attaching three cationic residues exhibited a reduction in activities against all tested strains, with an exception for analogue **19b** when compared with corresponding di-substituted compound **17b** against Gram-positive *S. aureus* SA38 strain and analogue **19c** when compared with corresponding di-substituted compound **17c** against Gram-negative *E. coli*. The reduction in activities could be explained by the repulsive interactions between the positive charged groups. In the case of **19a**, the shorter chain length of glycine residues may have resulted in the three repulsive charges being within closer proximity to one another, potentially negatively affecting the conformation of compound and its interaction with the bacterial cell membrane. Reduction in activities could also be due to a strong interaction between the cationic charges and the bacterial negatively charged headgroups. In the case of **19c** and **19d**, a total charge of +6 facilitates a much stronger electrostatic binding with the negatively charged membrane, that could potentially restrain deeper penetration of the AMP mimic into the inner leaflet of the membrane by a single hydrophobic tryptophan moiety.

### 2.3. Membrane Conduction

**Figure 4:**
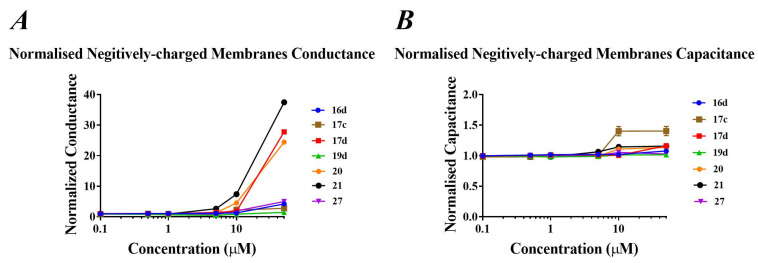
(**A**) Changes in membrane conduction and (**B**) changes in membrane capacitance in negative-charged tBLMs, in response to increasing concentrations of compounds **16d, 17c, 17d, 19d, 20, 21** and **27**.

Tethered bilayer lipid membranes (tBLMs), in association with AC electrical impedance spectroscopy techniques, were employed to measure changes in lipid bilayer conductance and capacitance in the presence of selected potent compounds (**16d, 17c, 17d, 19d, 20, 21, 27**) [30,31]. This experiment involved the use of negatively charged POPG lipids, which mimic the negatively-charged bacterial cell membrane. The responses to these selected compounds in terms of altering membrane conduction only became apparent at concentrations as high as ~50 µM (Figure 4A). Deeper penetration into the membrane due to high hydrophobicity facilitated deeper penetration was supported by non-tryptophan analogues **20** and **21**, as higher conduction changes were observed in negatively charged tBLMs. These responses can be considered small when compared to known pore-forming peptides such as PGLa when used on similar tBLM architecture, suggesting a transmembrane orientation described according to the barrel-stave model is less likely. Similarly, the relative responses in negatively charged tBLMs are modest in terms of capacitance changes (Figure 4B). Typically, increases in membrane capacitance indicate the tBLM is getting thinner, or there is an introduction of more water to the membrane. These data do suggest that these compounds are attracted to negatively charged lipid, like those that might be found in many bacterial membranes. The induction of increased overall membrane conduction with very small changes in membrane capacitance demonstrated that these compounds preferentially induce the formation of toroidal pore-like structures in membranes containing negatively charged lipids, either by themselves or by modulating the intrinsic pre-existing membrane pores via altering the critical packing parameter of the lipid bilayer. One molecule in particular, **17c**, had a significant impact on membrane capacitance, suggesting a more membrane lytic effect (Figure 4B).

### 2.4. Cytoplasmic Membrane Depolarisation

**Figure 5:**
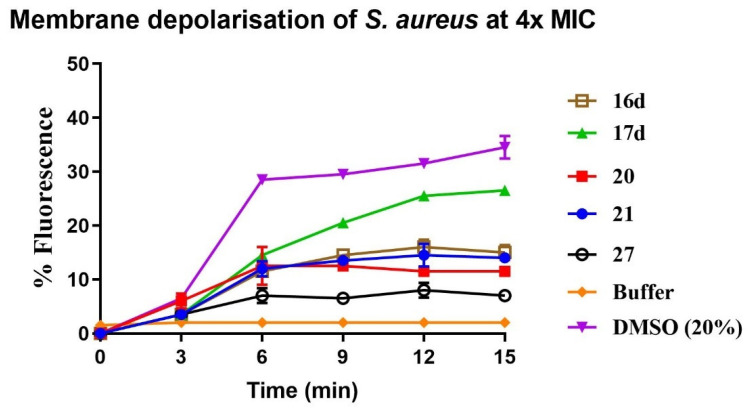
Percentage increase in fluorescence vs. time of membrane depolarisation of *S. aureus* with compounds **16d, 17c, 17d, 20, 21** and **27** at concentration of 4× MIC.

The mechanisms of actions of selected potent compounds (**16d, 17d, 20, 21, 27**) were further investigated using a membrane dye release assay on the cytoplasmic membrane of *S. aureus*. Firstly, 3,3′-dipropylthiadicarbocyanine iodine (diSC3-5) is a cationic membrane potential-sensitive fluorescent dye that can readily penetrate and accumulate in the polarised bacterial lipid bilayers [17,32]. This accumulation results in self-quenching of the overall level of fluorescence. The distribution of the membrane potential-sensitive dye between the cell membrane and periphery medium is dependent on the cytoplasmic membrane potential gradient. However, if an antimicrobial compound perturbs the bacterial cell membrane, the membrane potential gradient can be compromised, causing a rapid release of dye into the medium, resulting in dequenching. Therefore, an increase in the fluorescence intensity of the dye indicates membrane depolarisation. As shown in Figure 5, all tested compounds induced disruption of the cytoplasmic membrane of Gram-positive *S. aureus* in a time-dependent manner at four times (4×) of their MIC. The level of increase in fluorescence intensity was observed to be correlated with antimicrobial activity, with compounds **16d** and **17d** having lower MIC values showing greater increases in fluorescence intensity than compounds **20, 21** and **27** with higher MIC values. Amongst these tested compounds, compound **17d** was the most membrane active compound, as it induced the highest increase in fluorescence at 4× of its MIC at 15 min.

**Figure 6:**
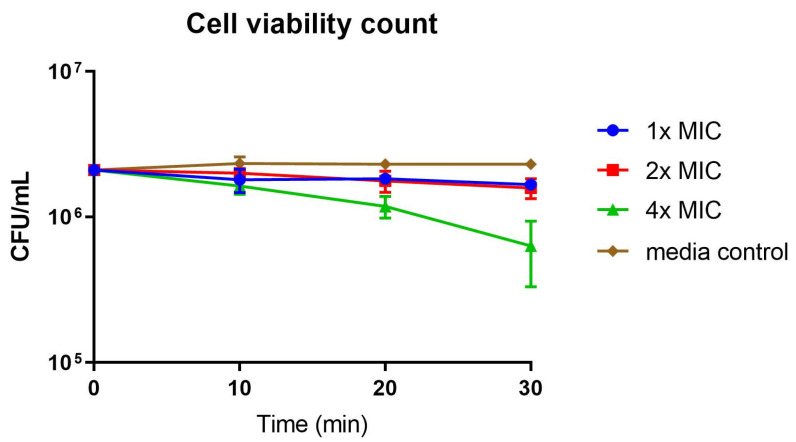
Cell viability count of *S. aureus* with the treatment of compound **17d** at 1×, 2× and 4× of its MIC.

Time-kill kinetic assay was also carried out to further investigate the bactericidal mechanism of the active compound **17d** (Figure 6). In this assay, *S. aureus* bacterial solution was treated with compound **17d** at 1×, 2× and 4× of its MIC, and the amount of viable bacterial cells was quantified over time. The bacterial cell viability upon the treatment of compound **17d** was time dependent and concentration dependent, with about 0.5-log reduction in viable bacteria at 4× of its MIC at 30 min. At lower concentrations of 1× and 2× of its MIC, the reductions in viable bacterial were modest.

Overall, these observations suggest that the tested compounds could act via permeabilisation of the bacterial cell membrane of *S. aureus*, which in turn causes cell death. However, these compounds could also exert their mechanism of action on various intracellular targets, and these will be investigated in future studies [33].

### 2.5. Cytotoxicity

The in vitro cytotoxicity of selected active compounds **16d** and **17d** was evaluated against MRC-5 normal human lung fibroblasts using the MTT assay at a concentration range of 1–500 μM. A dose–response curve was generated for each compound and the concentration of antimicrobial compound that kills 50% of the cell population (IC_50_) was determined. The selectivity ratio as the IC_50_ value divided by MIC value for each compound was then calculated (Table 2). A poor selectivity ratio of 1.24 was observed in compound **16d**, while **17d** obtained a higher selectivity ratio of 8.32. Compounds **16d** and **17d** varied in the number of lysine residues, and therefore an increase in the number of total net charges, showing a greater selectivity against bacterial cells. Whilst a higher selectivity from the scaffold is desirable, this result indicates a potential means of improving the selectivity ratio. The cytotoxicity results, along with the broad-spectrum activity of di-substituted cholic acid-based analogues, suggest that the scaffold is a promising candidate for the further development of antimicrobial drugs to treat human bacterial infection.

## 3. Materials and Methods

### 3.1. Synthesis of Analogues

All commercially available reagents were purchased from standard suppliers (Sigma Aldrich, St Louis, MO, USA and Alfa-Aesar, Ward Hill, MA, USA) and were used without further purification. All reactions were performed under anhydrous conditions with an atmosphere of nitrogen and anhydrous solvent (as required). Anhydrous solvents were obtained using PureSolv MD Solvent Purification System. Reactions were monitored by thin-layer chromatography precoated with Merck silica gel 60 F254 and visualization was performed by using short or long wavelength of ultraviolet light. Flash chromatography was performed using Grace Davisil LC60A silica.

Melting points were measured using an OptiMelt melting point apparatus and were uncorrected. ^1^H and ^13^C NMR spectra were obtained in the specified solvents on a Bruker Avance III 300, Bruker Avance III HD 400 or Bruker Avance III 600 Cryo spectrometer. Chemical shifts (δ) are in parts per million (ppm), internally referenced to the solvent nuclei. Multiplicities are assigned as singlet (s), broad singlet (bs), doublet (d), triplet (t), quartet (q), quintet (quint), sextet (sext), septet (sept), multiplet (m) or a combination of these (e.g., dd, dt, td), and coupling constants (*J*) are described in Hertz (Hz). Infrared (IR) spectra were recorded using a Cary 630 FTIR spectrometer fitted with a diamond attenuated total reflectance (ATR) sample interface. Low-resolution mass spectrometry was performed using a Thermo Fisher LCQ Mass Spectrometer while high-resolution mass spectrometry (HRMS) was performed by the UNSW Bioanalytical Mass Spectrometry Facility using a Thermo LTQ Orbitrap XL instrument.

#### 3.1.1. General Synthetic Procedure A for Coupling of Hydrophobic Groups

To a solution of appropriate cholic acid derivative (1.0 equivalent) in DMF (3 mL), EDC.HCl (1.1 equivalent) and DIPEA (2.0 equivalents) were added portion-wise at room temperature under nitrogen atmosphere. A solution of *L*-tryptophan methyl ester hydrochloride, *L*-phenylalanine methyl ester hydrochloride or benzylamine (1.5 equivalents) and DMAP (0.3 equivalent) in DMF (2 mL) was then added dropwise to the reaction mixture at 0 °C. The reaction mixture was stirred at room temperature overnight. After the completion of the reaction, the reaction mixture was poured into water (50 mL). The white precipitant was collected via vacuum filtration, redissolved in DCM (25 mL) and washed with NaHCO_3_ (2 × 50 mL), 2 M HCl (2 × 50 mL) and, finally, brine. The organic phase was then dried over anhydrous sodium sulfate and concentrated in vacuo to afford the product.

#### 3.1.2. General Synthetic Procedure B for N-Boc-Protected Esters

To a solution of substituted cholic acid derivatives (1 equivalent) in DCM (5 mL), EDC.HCl (1.5–8 equivalents), Boc-protected amino acids (1.5–8 equivalents) and DMAP (1.5–8 equivalents) was added portion-wise at room temperature under nitrogen atmosphere. The resulting suspension was stirred at room temperature for 16–96 h. After completion of reaction, water (100 mL) was added to the reaction mixture. The organic layer was extracted with DCM (3 × 25 mL), washed with brine (50 mL), dried over anhydrous sodium sulfate and concentrated in vacuo to provide the crude product. The crude product was then purified by flash column chromatography on silica using ethyl acetate/chloroform (25:75) as eluents to afford the *N*-Boc-protected esters.

#### 3.1.3. General Synthetic Procedure C for HCl Salts

To the Boc-protected product in DCM (10 mL), 4 M HCl in dioxane (3 mL) was added and the reaction mixture was stirred at room temperature for 2–4 h. After completion of reaction, the reaction mixture was concentrated in vacuo, triturated with diethyl ether and freeze-dried to afford the product as a HCl salt.

#### 3.1.4. General Synthetic Procedure D for Guanidinium Salt Compound

To a solution of GABA-substituted lithocholic ester (1.0 equivalent) and *N*,*N*′-di-Boc-1*H*-pyrazole-1-carboxamidine (1.3 equivalents) in anhydrous DMF (2 mL), triethylamine (2.5 equivalents) was added dropwise at 0 °C under nitrogen atmosphere. The reaction mixture was stirred at room temperature overnight. After the completion of the reaction, the reaction mixture was added into an ice-water mixture (50 mL) and the precipitate formed was collected by vacuum filtration to provide the crude product. The crude was then purified by flash column chromatography on silica using ethyl acetate/*n*-hexane (20–50%) to afford the Boc-protected product. To the Boc-protected product in DCM (5 mL), TFA (5 mL) was added at 0 °C. The reaction was stirred at room temperature for 3 h. After completion of reaction, the reaction mixture was concentrated in vacuo, triturated with diethyl ether and freeze-dried to afford the product as a TFA salt.

#### 3.1.5. General Synthetic Procedure E for Glycine-Lysine Monoester

To a solution of glycine-substituted lithocholic ester (1.0 equivalent) in DMF (3 mL), *N*,*N*′-di-Boc-*L*-lysine hydroxysuccinimide ester (1.2 equivalents) and DIPEA (4.0 equivalents) was added dropwise at 0 °C under nitrogen atmosphere. The reaction mixture was then stirred at room temperature overnight. After the completion of reaction, the reaction mixture was added to water (50 mL) and the white precipitant formed was collected via vacuum filtration, redissolved in DCM (25 mL), washed with brine (50 mL), dried over anhydrous sodium sulfate and concentrated in vacuo to provide the crude product. The crude product was purified by flash column chromatography on silica using ethyl acetate/*n*-hexane (25:75) to yield the Boc-protected product. To the Boc-protected product in DCM (10 mL), 4 M HCl in dioxane (3 mL) was added and stirred at room temperature for 4 h. After completion of reaction, the reaction mixture was concentrated in vacuo, triturated with diethyl ether and freeze-dried to afford the product as a HCl salt.

#### 3.1.6. General Synthetic Procedure F for Glycine-Valine Dipeptide

To a solution of glycine-substituted cholic acid triester (1.0 equivalent) in anhydrous DCM (3 mL), Boc-Val-OH (6.0 equivalents), EDC.HCl (6.0 equivalent), HOBt (3.0 equivalents) and DIPEA (5.0 equivalents) was added portion-wise at 0 °C under nitrogen atmosphere. The reaction was stirred at room temperature for 48 h. After the completion of the reaction, water (100 mL) was added to the reaction mixture and the product was extracted into DCM (3 × 25 mL), washed with saturated NaHCO_3_ (50 mL), 2 M HCl (50 mL) and brine (50 mL), dried over anhydrous sodium sulfate and concentrated in vacuo to give the crude product. The crude was purified by flash chromatography on silica using methanol/DCM (3%) as eluent to afford the Boc-protected product. To the Boc-protected product in DCM (10 mL), 4 M HCl in dioxane (3 mL) was added and stirred at room temperature for 4 h. After completion of the reaction, the reaction mixture was concentrated in vacuo, triturated with diethyl ether and freeze-dried to afford the product as a HCl salt.

#### 3.1.7. 2-(((3R,5R,8R,9S,10S,13R,14S,17R)-17-((R)-5-(((S)-3-(1H-Indol-3-yl)-1-methoxy-1-oxopropan-2-yl)amino)-5-oxopentan-2-yl)-10,13-dimethylhexadecahydro-1H-cyclopenta[α]phenanthren-3-yl)oxy)-2-oxoethan-1-aminium chloride (**16a**)

The title compound was synthesised from **10a** (0.300 g, 0.458 mmol) following general synthetic procedure C. The product **16a** was obtained as a white solid (0.195 g, 71%); mp 228.1 °C; ^1^H NMR (400 MHz, DMSO-*d*_6_) δ 0.60 (s, 3H, CH_3_), 0.86 (d, *J* = 6.4 Hz, 3H, CH_3_), 0.91 (s, 3H, CH_3_), 0.96–3.13 (m, CH, CH_2_ steroid), 2.97–3.18 (m, 2H, -CO-CH-CH_2_), 3.57 (s, 3H, OCH_3_), 3.76 (q, *J* = 5.6 Hz, 2H, -CO-CH_2_-NH_3_), 4.48 (td, *J* = 8.1, 5.8 Hz, 1H, -CO-CH-NH), 4.76 (tt, *J* = 11.1, 4.7 Hz, 1H, OCH), 6.98 (ddd, *J* = 8.0, 6.9, 1.1 Hz, 1H, ArH), 7.07 (ddd, *J* = 8.1, 6.9, 1.2 Hz, 1H, ArH), 7.14 (d, *J* = 2.4 Hz, 1H, ArH), 7.34 (d, *J* = 8.1 Hz, 1H, ArH), 7.49 (d, *J* = 7.8 Hz, 1H, ArH), 8.25 (d, *J* = 7.5 Hz, 1H, NH), 8.38 (d, *J* = 5.8 Hz, 3H, NH_3_), 10.91 (d, *J* = 2.4 Hz, 1H, NH-indole); ^13^C NMR (100 MHz, DMSO-*d*_6_) δ 173.2, 173.1, 167.5, 136.6, 127.5, 124.1, 121.4, 118.8, 118.5, 111.9, 110.1, 76.1, 66.8, 56.4, 56.1, 53.6, 52.2, 42.7, 41.5, 40.0, 39.8, 35.8, 35.4, 34.8, 34.6, 32.5, 32.2, 31.8, 28.2, 27.5, 27.0, 26.6, 26.4, 24.3, 23.4, 20.9, 18.8, 12.3; *ν*_max_ 3389, 2930, 2863, 2627, 2343, 1734, 1519, 1439, 1369, 1119, 1044, 973, 910, 745 cm^−1^; HRMS (+ESI): Found *m/z* 634.4214, [M]^+^, C_38_H_56_N_3_O_5_ required 634.4215.

#### 3.1.8. 4-(((3R,5R,8R,9S,10S,13R,14S,17R)-17-((R)-5-(((S)-3-(1H-Indol-3-yl)-1-methoxy-1-oxopropan-2-yl)amino)-5-oxopentan-2-yl)-10,13-dimethylhexadecahydro-1H-cyclopenta[α]phenanthren-3-yl)oxy)-4-oxobutan-1-aminium chloride (**16b**)

The title compound was synthesised from **10b** (0.273 g, 0.358 mmol) following general synthetic procedure C. The product **16b** was obtained as a white solid (0.198 g, 79%); mp 161.3 °C; ^1^H NMR (400 MHz, DMSO-*d*_6_) δ 0.60 (s, 3H, CH_3_), 0.86 (d, *J* = 6.4 Hz, 3H, CH_3_), 0.91 (s, 3H, CH_3_), 0.95–2.79 (m, CH, CH_2_, steroid), 3.02–3.14 (m, 2H, -CO-CH-CH_2_), 3.57 (s, 3H, OCH_3_), 4.48 (td, *J* = 8.1, 5.8 Hz, 1H, -CO-CH-NH), 4.65 (td, *J* = 11.3, 10.6, 4.9 Hz, 1H, -O-CH), 6.98 (ddd, *J* = 8.0, 7.0, 1.1 Hz, 1H, ArH), 7.07 (ddd, *J* = 8.1, 6.9, 1.2 Hz, 1H, ArH), 7.14 (d, *J* = 2.4 Hz, 1H, ArH), 7.34 (dt, *J* = 8.1, 0.9 Hz, 1H, ArH), 7.49 (d, *J* = 7.8 Hz, 1H, ArH), 8.00 (s, 3H, NH_3_), 8.24 (d, *J* = 7.6 Hz, 1H, NH), 10.90 (d, *J* = 2.5 Hz, 1H, NH-indole); ^13^C NMR (100 MHz, DMSO-*d*_6_) δ 173.2, 173.1 170.2, 136.6, 127.5, 124.1, 121.4, 118.8, 118.5, 111.9, 110.1, 74.8, 56.3, 56.1, 55.4, 53.6, 52.2, 42.7, 41.7, 40.0, 35.8, 35.3, 35.1, 34.9, 34.6, 32.5, 32.3, 32.1, 31.8, 31.0, 28.2, 27.3, 27.1, 26.7, 26.4, 24.3, 23.5, 20.9, 18.8, 12.3; *ν*_max_ 3390, 2928, 2863, 2321, 1934, 1721, 1665, 1521, 1440, 1356, 1294, 1204, 1118, 1020, 858 cm^−1^; HRMS (+ESI): Found *m/z* 662.4527, [M]^+^, C_40_H_60_N_3_O_5_ required 663.4528.

#### 3.1.9. (S)-5-(((3R,5R,8R,9S,10S,13R,14S,17R)-17-((R)-5-(((S)-3-(1H-Indol-3-yl)-1-methoxy-1-oxopropan-2-yl)amino)-5-oxopentan-2-yl)-10,13-dimethylhexadecahydro-1H-cyclopenta[α]phenanthren-3-yl)oxy)-5-oxopentane-1,4-diaminium chloride (**16c**)

The title compound was synthesised from **10c** (0.350 g, 0.393 mmol) following general synthetic procedure C. The product **16c** was obtained as a white solid (0.180 g, 67%); mp 185.7 °C; ^1^H NMR (400 MHz, DMSO-*d*_6_) δ 0.60 (s, 3H, CH_3_), 0.86 (d, *J* = 6.3 Hz, 3H, CH_3_), 0.92 (s, 3H, CH_3_), 0.97–2.79 (m, CH, CH_2_, steroid), 2.99–3.17 (m, 2H, -CO-CH-CH_2_), 3.57 (s, 3H, OCH_3_), 3.97 (t, *J* = 6.3 Hz, 1H, -CO-CH-NH_3_), 4.48 (td, *J* = 8.1, 5.7 Hz, 1H, -CO-CH-CH_2_), 4.75 (tt, *J* = 11.2, 4.6 Hz, 1H, OCH), 6.98 (t, *J* = 7.4 Hz, 1H, ArH), 7.06 (t, *J* = 7.5 Hz, 1H, ArH), 7.14 (d, *J* = 2.3 Hz, 1H, ArH), 7.34 (d, *J* = 8.1 Hz, 1H, ArH), 7.48 (d, *J* = 7.8 Hz, 1H, ArH), 8.25 (s, 1H, NH), 8.33 (m, 6H, NH), 10.93 (d, *J* = 2.4 Hz, 1H, NH-indole); ^13^C NMR (100 MHz, DMSO-*d*_6_) δ 173.2, 173.1, 169.3, 136.6, 127.5, 124.1, 121.4, 118.8, 118.4, 111.9, 110.0, 76.3, 56.4, 56.1, 53.6, 52.2, 52.2, 51.9, 42.7, 41.6, 40.2, 38.5, 35.8, 35.4, 34.8, 34.6, 32.5, 32.2, 31.8, 28.2, 27.7, 27.5, 27.1, 26.6, 26.5, 24.3, 23.4, 23.1, 20.9, 18.8, 12.3; IR (ATR): *ν*_max_ 3389, 2929, 2861, 2647, 2320, 2192, 2087, 1869, 1729, 1664, 1521, 1456, 1356, 1231, 1775, 1097, 933, 860 cm^−1^. HRMS (+ESI): Found *m/z* 692.4828, [M]^+2^, C_41_H_62_N_4_O_5_ required 692.4866.

#### 3.1.10. (S)-6-(((3R,5R,8R,9S,10S,13R,14S,17R)-17-((R)-5-(((S)-3-(1H-Indol-3-yl)-1-methoxy-1-oxopropan-2-yl)amino)-5-oxopentan-2-yl)-10,13-dimethylhexadecahydro-1H-cyclopenta[α]phenanthren-3-yl)oxy)-6-oxohexane-1,5-diaminium chloride (**16d**)

The title compound was synthesised from **10d** (0.527 g, 0.582 mmol) following general synthetic procedure C. The product **16d** was obtained as a white solid (0.416 g, 96%); mp 205.7 °C; ^1^H NMR (400 MHz, DMSO-*d*_6_) δ 0.60 (s, 3H, CH_3_), 0.86 (d, *J* = 6.4 Hz, 3H, CH_3_), 0.91 (s, 3H, CH_3_), 1.02–2.75 (m, CH, CH_2_, steroid), 2.98–3.17 (m, 2H, -CO-CH-CH_2_), 3.48 (s, 3H, OCH_3_), 3.92 (q, *J* = 5.8 Hz, 1H, -CO-CH-NH_3_), 4.48 (td, *J* = 8.1, 5.8 Hz, 1H, -CO-CH-NH), 4.76 (tt, *J* = 11.0, 4.6 Hz, 1H, OCH), 6.94–7.02 (m, 1H, ArH), 7.07 (ddd, *J* = 8.2, 6.9, 1.2 Hz, 1H, ArH), 7.14 (d, *J* = 2.3 Hz, 1H, ArH), 7.34 (d, *J* = 8.1 Hz, 1H, ArH), 7.48 (d, *J* = 7.9 Hz, 1H, ArH), 8.10 (d, *J* = 7.5 Hz, 3H, NH_3_), 8.26 (d, *J* = 7.5 Hz, 1H, NH), 8.57–8.67 (m, 3H, NH_3_), 10.93 (d, *J* = 2.4 Hz, 1H, NH-indole); ^13^C NMR (75 MHz, DMSO-*d*_6_) δ 173.2, 173.1, 169.3, 136.6, 127.5, 124.1, 121.4, 118.8, 118.4, 111.9, 110.0, 76.2, 66.8, 56.4, 56.1, 53.6, 52.2, 52.1, 52.1, 42.2, 41.6, 40.0, 38.6, 35.8, 35.4, 34.8, 34.6, 32.5, 32.2, 31.8, 29.8, 28.2, 27.5, 27.5, 27.1, 26.6, 24.3, 23.4, 21.6, 20.9, 18.8, 12.3; IR (ATR): *ν*_max_ 2857, 2281, 2171, 2109, 1722, 619, 1494, 1439, 1364, 1223, 1124, 1067, 1001, 892 cm^−1^; HRMS (+ESI): Found *m/z* 705.4953, [M]^2+^, C_42_H_64_N_4_O_5_ required 705.4950.

#### 3.1.11. 2,2′-(((3R,5S,7R,8R,9S,10S,13R,14S,17R)-17-((R)-5-(((S)-3-(1H-Indol-3-yl)-1-methoxy-1-oxopropan-2-yl)amino)-5-oxopentan-2-yl)-10,13-dimethylhexadecahydro-1H-cyclopenta[α]phenanthrene-3,7-diyl)bis(oxy))bis(2-oxoethan-1-aminium) chloride (**17a**)

The title compound was synthesised from **11a** (0.300 g, 0.331 mmol) following general synthetic procedure C. The product **17a** was obtained as a white solid (0.218 g, 85%); mp 253.1 °C; ^1^H NMR (400 MHz, DMSO-*d*_6_) δ 0.61 (s, 3H, CH_3_), 0.87 (d, *J* = 6.4 Hz, 3H, CH_3_), 0.92 (s, 3H, CH_3_), 1.02–2.11 (m, CH, CH_2_, steroid), 2.97–3.17 (m, 2H, -CO-CH-CH_2_), 3.57 (s, 3H, OCH_3_), 3.75–3.93 (m, 4H, 2 × -CO-CH_2_-NH_3_), 4.47 (td, *J* = 8.2, 5.7 Hz, 1H, -CO-CH-NH), 4.63 (dd, *J* = 10.5, 5.6 Hz, 1H, OCH), 4.92 (d, *J* = 3.6 Hz, 1H, OCH), 6.94–7.02 (m, 1H, ArH), 7.07 (ddd, *J* = 8.2, 7.0, 1.2 Hz, 1H, ArH), 7.14 (d, *J* = 2.3 Hz, 1H, ArH), 7.34 (d, *J* = 8.1 Hz, 1H, ArH), 7.48 (d, *J* = 7.8 Hz, 1H, ArH), 8.25 (d, *J* = 7.5 Hz, 1H, NH), 8.44 (d, *J* = 9.9 Hz, 6H, 3 × NH_3_), 10.91 (d, *J* = 2.4 Hz, 1H, NH-indole); ^13^C NMR (100 MHz, DMSO-*d*_6_) δ 173.2, 173.1, 167.7, 167.4, 136.6, 127.5, 124.1, 121.4, 118.8, 118.4, 111.9, 110.1, 75.8, 55.9, 53.6, 52.2, 50.1, 42.8, 37.5, 35.3, 34.8, 34.0, 27.5, 22.6, 18.8, 12.0; IR (ATR): *ν*_max_ 3181, 2928, 2861, 2621, 1734, 1651, 1498, 1434, 1367, 1230, 1118, 892 cm^−1^; HRMS (+ESI): Found *m/z* 354.2230, [M]^2+^, C_40_H_58_N_4_O_7_ required 354.2226.

#### 3.1.12. 4,4′-(((3R,5S,7R,8R,9S,10S,13R,14S,17R)-17-((R)-5-(((S)-3-(1H-Indol-3-yl)-1-methoxy-1-oxopropan-2-yl)amino)-5-oxopentan-2-yl)-10,13-dimethylhexadecahydro-1H-cyclopenta[α]phenanthrene-3,7-diyl)bis(oxy))bis(4-oxobutan-1-aminium) chloride (**17b**)

The title compound was synthesised from **11b** (0.760 g, 0.789 mmol) following general synthetic procedure C. The product **17b** was obtained as a white solid (0.467 g, 71%); mp 149.0 °C; ^1^H NMR (400 MHz, DMSO-*d*_6_) δ 0.60 (s, 3H, CH_3_), 0.86 (d, *J* = 6.3 Hz, 3H, CH_3_), 0.91 (s, 3H, CH_3_), 1.09–2.51 (m, CH, CH_2_, steroid), 2.80 (t, *J* = 6.9 Hz, 4H, 2 × -CH_2_-NH_3_), 2.98–3.17 (m, 2H, -CO-CH-CH_2_), 3.57 (s, 3H, OCH_3_), 4.50 (dtd, *J* = 21.5, 9.1, 8.0, 4.8 Hz, 2H, -OCH, -CO-CH-NH), 4.80 (q, *J* = 3.0 Hz, 1H, -OCH), 6.98 (t, *J* = 7.4 Hz, 1H, ArH), 7.02–7.11 (m, 1H, ArH), 7.15 (d, *J* = 2.4 Hz, 1H, ArH), 7.34 (d, *J* = 8.0 Hz, 1H, ArH), 7.48 (d, *J* = 7.8 Hz, 1H, ArH), 8.15 (d, *J* = 8.9 Hz, 6H, 2 × NH_3_), 8.28 (d, *J* = 7.5 Hz, 1H, NH), 10.93 (d, *J* = 2.4 Hz, 1H, NH-indole); ^13^C NMR (100 MHz, DMSO-*d*_6_) δ 173.2, 173.1, 172.1, 172.0, 136.6, 127.5, 124.1, 121.4, 118.8, 118.4, 111.9, 110.1, 74.0, 71.4, 65.4, 55.8, 53.6, 52.2, 50.6, 42.7, 40.6, 40.4, 40.2, 40.0, 39.8, 39.6, 39.4, 38.6, 38.5, 37.6, 35.3, 34.8, 34.8, 34.2, 32.4, 31.6, 31.3, 27.5, 23.0, 22.8, 18.8, 15.6, 12.0; *ν*_max_ 3745, 2954, 2832, 2475, 2285, 2103, 2177, 2103, 2047, 1995, 1944, 1719, 1589, 1492, 1236, 1068, 1128, 991, 916 cm^−1^; HRMS (+ESI): Found *m/z* 763.5000, [M]^+^, C_44_H_66_N_4_O_7_ required 763.5004.

#### 3.1.13. (4S,4′S)-5,5′-(((3R,5S,7R,8R,9S,10S,13R,14S,17R)-17-((R)-5-(((S)-3-(1H-Indol-3-yl)-1-methoxy-1-oxopropan-2-yl)amino)-5-oxopentan-2-yl)-10,13-dimethylhexadecahydro-1H-cyclopenta[α]phenanthrene-3,7-diyl)bis(oxy))bis(5-oxopentane-1,4-diaminium) chloride (**17c**)

The title compound was synthesised from **11c** (0.511 g, 0.418 mmol) following general synthetic procedure C. The product **17c** was obtained as a white solid (0.305 g, 75%); mp 235.9 °C; ^1^H NMR (400 MHz, DMSO-*d*_6_) δ 0.60 (s, 3H, CH_3_), 0.87 (d, *J* = 6.2 Hz, 3H, CH_3_), 0.92 (d, *J* = 3.9 Hz, 3H, CH_3_), 1.23– 2.18 (m, CH, CH_2_, steroid), 2.83 (m, 4H, 2 × -CH_2_-NH_3_), 2.97–3.19 (m, 2H, CH_2_-CH-NH), 3.57 (d, *J* = 2.8 Hz, 3H, OCH_3_), 3.96–4.13 (m, 2H, 2 × CH-NH_3_), 4.45 (ddd, *J* = 11.8, 8.1, 6.1 Hz, 1H, CO-CH-NH), 4.55–4.70 (m, 1H, OCH), 4.87 (q, *J* = 2.9 Hz, 1H, OCH), 6.95–7.01 (m, 1H, ArH), 7.03–7.10 (m, 1H, ArH), 7.15 (dd, *J* = 5.0, 2.4 Hz, 1H, ArH), 7.34 (d, *J* = 8.1 Hz, 1H, ArH), 7.48 (q, *J* = 8.6, 7.6 Hz, 1H, ArH), 8.27–8.34 (m, 6H, 2 × NH_3_), 8.70–8.92 (m, 7H, 2 × NH_3_, NH), 10.97 (d, *J* = 2.4 Hz, 1H, NH-indole); ^13^C NMR (100 MHz, DMSO-*d*_6_) δ 173.2, 173.1, 169.3, 136.6, 129.5, 127.5, 124.2, 121.4, 121.0, 118.8, 118.7, 112.0, 110.0, 76.5, 76.2, 74.3, 73.8, 65.4, 55.9, 55.8, 53.6, 52.6, 52.2, 52.0, 51.9, 51.8, 49.9, 42.9, 42.8, 38.7, 37.7, 34.9, 34.8, 29.8, 23.7, 23.2, 23.1, 22.6, 22.5, 20.8, 18.8, 15.6, 12.0, 12.0; IR (ATR): *ν*_max_ 3745, 2868, 2653, 2284, 2171, 2041, 1730, 1596, 1496, 1456, 1365, 1223, 1139, 1048, 1001, 890 cm^−1^; HRMS (+ESI): Found *m/z* 411.2809, [M]^4+^, C_46_H_74_N_6_O_7_ required 411.2804.

#### 3.1.14. (5S,5′S)-6,6′-(((3R,5S,7R,8R,9S,10S,13R,14S,17R)-17-((R)-5-(((S)-3-(1H-Indol-3-yl)-1-methoxy-1-oxopropan-2-yl)amino)-5-oxopentan-2-yl)-10,13-dimethylhexadecahydro-1H-cyclopenta[α]phenanthrene-3,7-diyl)bis(oxy))bis(6-oxohexane-1,5-diaminium) chloride (**17d**)

The title compound was synthesised from **11d** (0.061 g, 0.048 mmol) following general synthetic procedure C. The product **17d** was obtained as a white solid (0.041 g, 85%); mp 244.2 °C; ^1^H NMR (600 MHz, DMSO-*d*_6_) δ 0.60 (s, *J* = 5.0 Hz, 3H, CH_3_), 0.87 (d, *J* = 6.1 Hz, 3H, CH_3_), 0.92 (s, 3H, CH_3_), 0.98–2.07 (m, steroid), 2.75–2.81 (m, 4H, 2 × -CH_2_-NH_3_), 3.03–3.15 (m, 2H, -CO-CH-CH_2_), 3.57 (s, 3H, OCH_3_), 3.90 (d, *J* = 5.7 Hz, 1H, -CH-NH_3_), 4.05 (t, *J* = 5.8 Hz, 1H, -CH-NH_3_), 4.46 (td, *J* = 8.0, 5.8 Hz, 1H, -CO-CH-NH), 4.60 (dt, *J* = 11.3, 6.4 Hz, 1H, OCH), 4.89 (t, *J* = 3.3 Hz, 1H, OCH), 6.98 (t, *J* = 7.5 Hz, 1H, ArH), 7.07 (t, *J* = 7.5 Hz, 1H, ArH), 7.15 (dd, *J* = 7.7, 2.4 Hz, 1H, ArH), 7.35 (d, *J* = 8.1 Hz, 1H, ArH), 7.48 (d, *J* = 8.0 Hz, 1H, ArH), 8.07–8.19 (m, 6H, 2 × NH_3_), 8.30 (dd, *J* = 28.8, 7.5 Hz, 1H, NH), 8.63–8.73 (m, 6H, 2 × NH_3_), 10.93 (d, *J* = 2.5 Hz, 1H, NH); ^13^C NMR (150 MHz, DMSO-*d*_6_) δ 173.4, 173.1, 169.2, 136.6, 127.5, 124.2, 124.1, 121.4, 118.9, 118.4, 112.0, 110.0, 76.1, 74.1, 72.7, 72.6, 71.0, 66.8, 65.4, 60.7, 60.6, 53.7, 52.6, 52.3, 52.2, 50.4, 44.1, 42.8, 42.7, 38.7, 38.6, 35.4, 34.8, 34.6, 34.5, 33.9, 32.6, 29.8, 28.0, 27.5, 26.7, 26.7, 23.7, 22.7, 22.5, 18.8, 15.6, 12.0; IR (ATR): *ν*_max_ 3365, 3236, 2930, 2865, 2072, 1733, 1638, 1510, 1457, 1218, 1137, 1065, 999 cm^−1^; HRMS (+ESI): Found *m/z* 425.2962, [M]^4+^, C_48_H_72_N_6_O_7_ required 425.2961.

#### 3.1.15. 2,2′-(((3R,5R,8R,9S,10S,12S,13R,14S,17R)-17-((R)-5-(((S)-3-(1H-Indol-3-yl)-1-methoxy-1-oxopropan-2-yl)amino)-5-oxopentan-2-yl)-10,13-dimethylhexadecahydro-1H-cyclopenta[α]phenanthrene-3,12-diyl)bis(oxy))bis(2-oxoethan-1-aminium) chloride (**18a**)

The title compound was synthesised from **12a** (0.173 g, 0.191 mmol) following general synthetic procedure C. The product **18a** was obtained as a white solid (0.130 g, 87%); mp 242.9 °C; ^1^H NMR (300 MHz, DMSO-*d*_6_) δ 0.69 (s, 3H, CH_3_), 0.75 (d, *J* = 6.2 Hz, 3H, CH_3_), 0.90 (s, 3H, CH_3_), 1.01– 2.19 (m, CH, CH_2,_ steroid), 2.98–3.20 (m, 2H, -CO-CH-CH_2_), 3.57 (s, 3H, OCH_3_), 3.74 (d, *J* = 5.2 Hz, 2H, -CO-CH_2_-NH_3_), 3.83–3.95 (m, 2H, -CO-CH_2_-NH_3_), 4.47 (td, *J* = 8.1, 5.8 Hz, 1H, -CO-CH-NH), 4.72 (tt, *J* = 10.8, 5.1 Hz, 1H, OCH), 5.13 (d, *J* = 2.8 Hz, 1H, OCH), 6.98 (td, *J* = 7.4, 7.0, 1.1 Hz, 1H, ArH), 7.07 (ddd, *J* = 8.1, 7.0, 1.2 Hz, 1H, ArH), 7.16 (d, *J* = 2.4 Hz, 1H, ArH), 7.34 (d, *J* = 8.0 Hz, 1H, ArH), 7.48 (d, *J* = 7.8 Hz, 1H, ArH), 8.28 (d, *J* = 7.5 Hz, 1H, ArH), 8.47–8.63 (m, 6H, 2 × NH_3_), 10.97 (d, *J* = 2.4 Hz, 1H, NH-indole); ^13^C NMR (75 MHz, DMSO-*d*_6_) δ 173.3, 173.1, 167.5, 167.4, 136.6, 127.5, 124.1, 121.4, 118.8, 118.4, 111.9, 110.0, 77.6, 76.2, 65.4, 53.6, 52.2, 49.0, 47.4, 45.1, 41.5, 40.0, 40.0, 35.6, 34.9, 34.6, 34.2, 32.4, 32.1, 31.5, 27.5, 27.0, 26.5, 26.0, 25.7, 23.5, 23.1, 17.8, 15.6, 12.4; IR (ATR): *ν*_max_ 3237, 2923, 2863, 2321, 1920, 1735, 1640, 1509, 1435, 1367, 1236, 1120, 1049, 967, 913 cm^−1^; HRMS (+ESI): Found *m/z* 354.2226, [M]^2+^, C_40_H_58_N_4_O_7_ required 354.2226.

#### 3.1.16. 2,2′-(((3R,5S,7R,8R,9S,10S,12S,13R,14S,17R)-17-((R)-5-(((S)-3-(1H-Indol-3-yl)-1-methoxy-1-oxopropan-2-yl)amino)-5-oxopentan-2-yl)-3-(2-aminoacetoxy)-10,13-dimethylhexadecahydro-1H-cyclopenta[α]phenanthrene-7,12-diyl)bis(oxy))bis(2-oxoethan-1-aminium) chloride (**19a**)

The title compound was synthesised from **13a** (0.500 g, 0.559 mmol) following general synthetic procedure C. The product **19a** was obtained as a white solid (0.426 g, 87%); mp 221.4 °C; ^1^H NMR (400 MHz, DMSO-*d*_6_) δ 0.69 (s, 3H), 0.76 (d, *J* = 6.3 Hz, 3H), 0.92 (s, 3H), 1.03–3.17 (m, CH, CH_2_, steroid), 3.57 (s, 3H, OCH_3_), 3.70–4.03 (m, 6H, 3 × -CO-CH_2_-NH_3_), 4.46 (td, *J* = 8.0, 5.6 Hz, 1H, -CO-CH-NH), 4.59 (tq, *J* = 9.0, 4.3 Hz, 1H, OCH), 4.89–4.96 (m, 1H, OCH), 5.13 (t, *J* = 2.9 Hz, 1H, OCH), 6.98 (t, *J* = 7.4 Hz, 1H, ArH), 7.07 (t, *J* = 7.5 Hz, 1H, ArH), 7.16 (d, *J* = 2.4 Hz, 1H, ArH), 7.35 (d, *J* = 8.1 Hz, 1H, ArH), 7.48 (d, *J* = 7.9 Hz, 1H, ArH), 8.27 (d, *J* = 7.4 Hz, 1H, NH), 8.44–8.70 (m, *J* = 7.5, 7.1 Hz, 9H, 3 × NH_3_), 10.97 (d, *J* = 2.4 Hz, 1H, NH-indole); ^13^C NMR (100 MHz, DMSO-*d*_6_) δ 124.1, 121.4, 118.9, 118.4, 111.9, 77.3, 76.0, 73.2, 72.7, 72.7, 69.7, 66.8, 66.8, 60.7, 52.2, 47.4, 43.0, 40.6, 40.4, 40.3, 40.2, 40.0, 39.8, 37.2, 34.8, 28.6, 27.5, 22.5, 17.7, 12.2; *ν*_max_ 3194, 2936, 2859, 2616, 2342, 1734, 1640, 1597, 1508, 1435, 1368, 1232, 1117, 1039, 742 cm^−1^; HRMS (+ESI): Found *m/z* 390.7312, [M]^3+^, C_42_H_63_N_5_O_9_ required 390.7307.

#### 3.1.17. 4,4′,4″-(((3R,5S,7R,8R,9S,10S,12S,13R,14S,17R)-17-((R)-5-(((S)-3-(1H-Indol-3-yl)-1-methoxy-1-oxopropan-2-yl)amino)-5-oxopentan-2-yl)-10,13-dimethylhexadecahydro-1H-cyclopenta[α]phenanthrene-3,7,12-triyl)tris(oxy))tris(4-oxobutan-1-aminium) chloride (**19b**)

The title compound was synthesised from **13b** (0.138 g, 0.551 mmol) following general synthetic procedure C. The product **19b** was obtained as a white solid (0.094 g, 81%); mp 212.2 °C; ^1^H NMR (400 MHz, DMSO-*d*_6_) δ 0.64–0.69 (m, 3H, CH_3_), 0.73 (d, *J* = 6.2 Hz, 3H, CH_3_), 0.88 (d, *J* = 15.8 Hz, 3H, CH_3_), 1.01– 2.64 (m, CH, CH_2_, steroid), 2.78–2.91 (m, 6H, 3 × CH_2_-NH_3_), 3.02–3.15 (m, 2H, CO-CH-CH_2_), 3.57 (s, 3H, OCH_3_), 4.48 (dtd, *J* = 15.8, 9.0, 8.1, 4.7 Hz, 2H, OCH, CO-CH-NH), 4.76–4.86 (m, 1H, OCH), 4.99 (dt, *J* = 7.0, 2.9 Hz, 1H, OCH), 6.98 (t, *J* = 7.4 Hz, 1H, ArH), 7.06 (t, *J* = 7.5 Hz, 1H, ArH), 7.17 (q, *J* = 2.6 Hz, 1H, ArH), 7.34 (d, *J* = 8.0 Hz, 1H, ArH), 7.50 (dd, *J* = 17.0, 7.8 Hz, 1H, NH), 8.12–8.31 (m, 9H, NH_3_), 10.90–10.98 (m, 1H, NH-indole); ^13^C NMR (100 MHz, DMSO-*d*_6_) δ 124.2, 121.4, 118.8, 118.4, 111.9, 75.2, 73.8, 71.0, 66.8, 66.8, 53.6, 52.2, 47.3, 43.5, 40.6, 40.5, 40.3, 40.0, 38.7, 38.6, 37.3, 34.8, 34.5, 32.2, 31.7, 31.6, 31.4, 31.2, 31.0, 28.7, 27.5, 27.1, 26.9, 25.5, 23.2, 23.0, 22.9, 22.8, 22.6, 17.9, 12.3; *ν*_max_ 2932, 2071, 1934, 1719, 1639, 1510, 1438, 1379, 1191, 1113, 1057, 1005, 749 cm^−1^; HRMS (+ESI): Found *m/z* 288.8543, [M]^3+^, C_48_H_76_N_5_O_9_ required 288.8542.

#### 3.1.18. (4S,4′S,4″S)-5,5′,5″-(((3R,5S,7R,8R,9S,10S,12S,13R,14S,17R)-17-((R)-5-(((S)-3-(1H-Indol-3-yl)-1-methoxy-1-oxopropan-2-yl)amino)-5-oxopentan-2-yl)-10,13-dimethylhexadecahydro-1H-cyclopenta[α]phenanthrene-3,7,12-triyl)tris(oxy))tris(5-oxopentane-1,4-diaminium) chloride (**19c**)

The title compound was synthesised from **13c** (0.200 g, 0.129 mmol) following general synthetic procedure C. The product **19c** was obtained as a white solid (0.113 g, 75%); mp 241.6 °C; ^1^H NMR (600 MHz, DMSO-*d*_6_) δ 0.70 (d, *J* = 1.9 Hz, 3H, -CH_3_), 0.75 (dd, *J* = 16.3, 6.6 Hz, 3H, CH_3_), 0.93 (d, *J* = 7.8 Hz, 3H, CH_3_), 1.02–2.41 (m, CH, CH_2_, steroid), 2.88 (d, *J* = 32.7 Hz, 4H, 2 × -CH_2_-NH_3_), 3.00–3.18 (m, 4H, -CH_2_-NH_3,_ -CO-CH-CH_2_), 3.57 (d, *J* = 1.5 Hz, 3H, -OCH_3_), 4.15 (d, *J* = 5.5 Hz, 1H, -CO-CH-NH_3_), 4.32 (s, 1H, -CO-CH-NH_3_), 4.45 (m, *J* = 7.5, 5.9, 3.5 Hz, 2H, -OCH, -CO-CH-NH_3_), 4.59 (t, *J* = 5.6 Hz, 1H, -CO-CH-NH), 5.05 (dq, *J* = 20.3, 3.0 Hz, 1H, OCH), 5.15 (q, *J* = 2.9 Hz, 1H, OCH), 6.98 (ddd, *J* = 8.0, 7.0, 1.1 Hz, 1H, ArH), 7.07 (ddd, *J* = 8.1, 6.9, 1.2 Hz, 1H, ArH), 7.17 (q, *J* = 4.8 Hz, 1H, ArH), 7.34–7.36 (m, 1H, ArH), 7.48 (d, *J* = 7.9 Hz, 1H, ArH), 8.27–8.41 (m, 10H, -NH, 3 × NH_3_), 8.69–8.87 (m, 9H, 3 × NH_3_), 10.93–10.99 (m, 1H, NH-indole); ^13^C NMR (150 MHz, DMSO-*d*_6_) δ 173.3, 173.1, 169.3, 169.2, 169.1, 167.7, 136.6, 127.5, 124.2, 121.4, 118.8, 118.4, 112.0, 110.0, 77.6, 76.7, 73.0, 53.6, 52.2, 51.9, 51.9, 51.8, 49.3, 47.4, 45.3, 45.3, 45.2, 42.6, 41.3, 38.8, 38.6, 38.4, 37.6, 35.0, 34.7, 32.6, 31.6, 31.5, 28.4, 27.8, 27.4, 27.1, 23.5, 22.9, 22.8, 22.4, 22.2, 20.7, 17.7, 12.3, 12.2; *ν*_max_ 3373, 2863, 2079, 1948, 1731, 1599, 1509, 1457, 1365, 1226, 1128, 1004, 894 cm^−1^; HRMS (+ESI): Found *m/z* 317.8809, [M]^6+^, C_51_H_85_N_8_O_9_ required 317.8808.

#### 3.1.19. (5S,5′S,5″S)-6,6′,6″-(((3R,5S,7R,8R,9S,10S,12S,13R,14S,17R)-17-((R)-5-(((S)-3-(1H-Indol-3-yl)-1-methoxy-1-oxopropan-2-yl)amino)-5-oxopentan-2-yl)-10,13-dimethylhexadecahydro-1H-cyclopenta[α]phenanthrene-3,7,12-triyl)tris(oxy))tris(6-oxohexane-1,5-diaminium) chloride (**19d**)

The title compound was synthesised from **13d** (0.282 g, 0.177 mmol) following **general synthetic procedure C**. The product **19d** was obtained as a white solid (0.185 g, 86%); mp 243.3 °C; ^1^H NMR (600 MHz, DMSO-*d*_6_) δ 0.70 (d, *J* = 3.0 Hz, 3H, CH_3_), 0.73–0.79 (m, 3H, CH_3_), 0.92 (dd, *J* = 6.6, 2.6 Hz, 3H, CH_3_), 1.05–2.26 (m, CH, CH_2_, steroid), 2.77–2.84 (m, 6H, 3 × -CH_2_-NH_3_), 3.01–3.15 (m, 2H, -CO-CH-CH_2_), 3.38 (d, *J* = 7.0 Hz, 3H, 3 × -CO-CH-NH_3_), 3.57 (s, 3H, OCH_3_), 3.74–5.19 (m, 4H, -OCH, -CO-CH-NH), 6.95–7.01 (m, 1H, ArH), 7.06 (ddd, *J* = 8.1, 6.9, 1.1 Hz, 1H, ArH), 7.17 (q, *J* = 3.5, 2.9 Hz, 1H, ArH), 7.35 (d, *J* = 8.1 Hz, 1H, ArH), 7.47 (d, *J* = 7.9 Hz, 1H, ArH), 8.04–8.14 (m, 3H, NH_3_), 8.19–8.25 (m, 6H, 2 × NH_3_), 8.33 (dt, *J* = 7.9, 3.2 Hz, 1H, NH), 8.65–8.90 (m, 9H, 3 × NH_3_), 10.97 (d, *J* = 2.2 Hz, 1H, NH-indole). ^13^C NMR (150 MHz, DMSO-*d*_6_) δ 173.4, 173.3, 173.1, 172.5, 169.3, 136.6, 127.5, 124.2, 121.4, 118.8, 118.4, 112.0, 110.0, 77.6, 76.7, 73.0, 65,4, 53.7, 52.2, 45.3, 45.3, 45.2, 42.8, 38.8, 38.8, 38.6, 38.4, 37.5, 35.0, 34.8, 32.6, 34.5, 34.3, 32.7, 31.7, 31.5, 31.1, 30.8, 29.8, 29.8, 29.3, 28.3, 27.6, 27.4, 26.7, 26.5, 25.6, 22.4, 22.4, 21.7, 17.7, 15.6, 12.3, 12.2; *ν*_max_ 3377, 2920, 2866, 2045, 1732, 1602, 1509, 1456, 1364, 1224, 1129, 1068, 994, 893 cm^−1^; HRMS (+ESI): Found *m/z* 331.8966, [M]^6+^, C_54_H_91_N_8_O_9_ required 331.8964.

#### 3.1.20. (4S,4′S)-5,5′-(((3R,5S,7R,8R,9S,10S,13R,14S,17R)-17-((R)-5-(((S)-1-Methoxy-1-oxo-3-phenylpropan-2-yl)amino)-5-oxopentan-2-yl)-10,13-dimethylhexadecahydro-1H-cyclopenta[α]phenanthrene-3,7-diyl)bis(oxy))bis(5-oxopentane-1,4-diaminium) chloride (**20**)

The title compound was synthesised from **14** (0.783 g, 0.662 mmol) following general procedure C. The product **20** was obtained as a white solid (0.532 g, 87%); mp 236.6 °C; ^1^H NMR (400 MHz, DMSO-*d*_6_) δ 0.60 (s, 3H, CH_3_), 0.85 (d, *J* = 6.3 Hz, 3H, CH_3_), 0.92 (d, *J* = 4.2 Hz, 3H, CH_3_), 1.05–2.92 (m, CH, CH_2_, steroid), 2.96–3.06 (m, 2H, -CO-CH-CH_2_), 3.58 (d, *J* = 1.0 Hz, 3H, OCH_3_), 3.89–4.19 (m, 2H, CH, CH-NH_3_), 4.36–4.70 (m, 3H, 2 × OCH, -CO-CH-CH_2_), 4.87 (d, *J* = 3.2 Hz, 1H, CH), 7.21 (d, *J* = 2.0 Hz, 1H, ArH), 7.23 (d, *J* = 2.0 Hz, 2H, ArH), 7.26 (s, 1H, NH), 7.27 (d, *J* = 1.4 Hz, 1H, ArH), 7.30 (t, *J* = 3.2 Hz, 1H, ArH), 8.26–8.38 (m, 6H, NH_3_), 8.81 (d, *J* = 35.9 Hz, 6H, NH_3_); ^13^C NMR (100 MHz, DMSO-*d*_6_) δ 173.2, 173.2, 172.7, 171.8, 169.4, 169.3, 169.1, 168.8, 137.9, 137.4, 129.6, 129.5, 128.8, 128.7, 127.2, 127.0, 76.5, 55.9, 54.7, 54.1, 52.6, 52.2, 52.0, 51.2, 49.8, 42.8, 42.8, 38.7, 38.5, 38.1, 37.7, 37.1, 36.8, 35.4, 34.9, 34.8, 34.4, 33.4, 23.2, 22.6, 22.5, 18.8, 12.0, 12.0; IR (ATR): *ν*_max_ 3374, 2929, 2865, 2061, 1733, 1639, 1510, 1449, 1366, 1223, 1138, 1065, 997; HRMS (+ESI): Found *m/z* 391.7755, [M]^4+^, C_44_H_73_N_5_O_7_ required 391.7745.

#### 3.1.21. (4S,4′S)-5,5′-(((3R,5S,7R,8R,9S,10S,13R,14S,17R)-17-((R)-5-(Benzylamino)-5-oxopentan-2-yl)-10,13-dimethylhexadecahydro-1H-cyclopenta[a]phenanthrene-3,7-diyl)bis(oxy))bis(5-oxopentane-1,4-diaminium) chloride (**21**)

The title compound was synthesised from **15** (0.520 g, 0.468 mmol) following general procedure C. The product **21** was obtained as a white solid (0.374 g, 93%); mp 208.3 °C; ^1^H NMR (400 MHz, DMSO-*d*_6_) δ 0.61 (s, 3H, CH_3_), 0.88–0.94 (m, 6H, 2 × CH_3_), 1.02–2.23 (m, CH, CH_2_, steroid), 2.82 (dq, *J* = 12.8, 6.4 Hz, 4H, 2 × CH_2-_NH_3_), 3.95–4.31 (m, 5H, OCH, 2 × CH-NH_3_, NH-CH_2_), 4.53–4.71 (m, 1H, OCH), 7.21–7.32 (m, 5H, ArH), 8.21–8.34 (m, 6H, NH_3_), 8.42 (dt, *J* = 14.5, 6.0 Hz, 1H, NH), 8.78 (dd, *J* = 26.3, 12.0 Hz, 6H, NH_3_); IR (ATR): *ν*_max_ 3369, 2927, 2862, 1942, 1733, 1509, 1601, 1509, 1450, 1378, 1226, 1138, 1065, 962, 916; HRMS (+ESI): Found *m/z* 355.7646, [M]^4+^, C_41_H_69_N_5_O_5_ required 355.7644.

#### 3.1.22. ((4-(((3R,5R,8R,9S,10S,13R,14S,17R)-17-((R)-5-(((S)-3-(1H-Indol-3-yl)-1-methoxy-1-oxopropan-2-yl)amino)-5-oxopentan-2-yl)-10,13-dimethylhexadecahydro-1H-cyclopenta[α]phenanthren-3-yl)oxy)-4-oxobutyl)amino)(amino)methaniminium trifluoroacetate (**23**)

The title compound was synthesised from **16b** (0.213 g, 0.305 mmol), *N*,*N*′-di-Boc-1*H*-pyrazole-1-carboxamidine (0.125 g, 0.396 mmol) and triethylamine (1 mL, 0.762 mmol) following general synthetic procedure D. The product **23** was obtained as a white solid (0.105 g, 53%); mp 247.9 °C; ^1^H NMR (400 MHz, DMSO-*d*_6_) δ 0.60 (s, 3H, CH_3_), 0.86 (d, *J* = 6.3 Hz, 3H, CH_3_), 0.91 (s, 3H, CH_3_), 1.04–2.55 (m, CH, CH_2,_ steroid), 3.02–3.12 (m, 2H, -CO-CH-CH_2_), 3.57 (s, 3H, OCH_3_), 4.48 (q, *J* = 7.2 Hz, 1H, -CO-CH-NH), 4.56–4.73 (m, 1H, OCH), 6.81–7.72 (m, 10H, ArH, NH_2_, NH), 8.22 (d, *J* = 7.6 Hz, 1H, NH), 10.87 (s, 1H, NH-indole); ^13^C NMR (75 MHz, DMSO-*d*_6_) δ 173.2, 173.1, 172.3, 170.9, 157.3, 136.6, 127.6, 124.1, 121.4, 118.8, 118.5, 111.9, 110.1, 56.4, 56.1, 53.6, 52.2, 42.7, 41.7, 40.8, 40.5, 40.3, 40.0, 39.7, 39.1, 35.8, 35.4, 35.0, 34.7, 33.9, 32.5, 31.3, 27.5, 27.1, 26.4, 24.5, 24.3, 23.5, 20.9, 18.8, 12.3; IR (ATR): *ν*_max_ 3275, 3184, 2930, 2863, 2320, 1654, 1533, 1439, 1357, 1175, 1132, 800 cm^−1^; HRMS (+ESI): Found *m/z* 704.4746, [M]^+^, C_41_H_61_N_5_O_5_ required 704.4746.

#### 3.1.23. (S)-6-((2-(((3R,5R,8R,9S,10S,13R,14S,17R)-17-((R)-5-(((S)-3-(1H-Indol-3-yl)-1-methoxy-1-oxopropan-2-yl)amino)-5-oxopentan-2-yl)-10,13-dimethylhexadecahydro-1H-cyclopenta[α]phenanthren-3-yl)oxy)-2-oxoethyl)amino)-6-oxohexane-1,5-diaminium chloride (**25**)

The title compound was synthesised from **16a** (0.150 g, 0.224 mmol), *N*_ε_-Z-*N*_α_-Boc-*L*-lysine hydroxysuccinimide ester (0.119 g, 0.269 mmol) and DIPEA (0.116 g, 0.895 mmol) following general synthetic procedure E. The product **25** was obtained as a white solid (0.027 g, 15%); mp 247.9 °C; ^1^H NMR (400 MHz, DMSO-*d*_6_) δ 0.60 (s, 3H, CH_3_), 0.86 (d, *J* = 6.4 Hz, 3H, CH_3_), 0.91 (s, 3H, CH_3_), 1.01–2.15 (m, steroid), 2.75 (q, *J* = 8.3, 7.2 Hz, 2H, -CH2-NH3), 3.02–3.13 (m, 2H, -CO-CH-CH_2_), 3.57 (s, 3H, -OCH_3_), 3.82–3.90 (m, 2H, -CH_2-_NH), 3.96 (dd, *J* = 17.5, 5.9 Hz, 1H, -CH-NH_3_), 4.48 (td, *J* = 8.1, 5.8 Hz, 1H, -CO-CH-NH), 4.62–4.72 (m, 1H, -OCH), 6.98 (t, *J* = 7.4 Hz, 1H, ArH), 7.07 (t, *J* = 7.5 Hz, 1H, ArH), 7.14 (d, *J* = 2.4 Hz, 1H, ArH), 7.34 (d, *J* = 8.1 Hz, 1H, ArH), 7.49 (d, *J* = 7.9 Hz, 1H, ArH), 8.05 (s, 3H, NH_3_), 8.25 (d, *J* = 7.6 Hz, 1H, NH), 8.35 (d, *J* = 5.3 Hz, 3H, NH_3_), 9.05 (t, *J* = 5.8 Hz, 1H, NH), 10.90 (d, *J* = 2.3 Hz, 1H, NH-indole); ^13^C NMR (75 MHz, DMSO-*d*_6_) δ 173.2, 173.1, 169.6, 169.3, 136.6, 127.5, 124.1, 121.4, 118.8, 118.5, 111.9, 110.1, 75.1, 66.8, 66.8, 65.4, 56.4, 56.1, 53.6, 52.2, 52.1, 42.7, 41.6, 40.8, 40.5, 40.3, 40.0, 39.7, 39.4, 39.1, 38.7, 35.8, 35.4, 34.9, 34.6, 30.8, 27.5, 26.7, 23.5, 21.3, 20.9, 18.7, 15.6, 12.3; IR (ATR): *ν*_max_ 3229, 3016, 2926, 2863, 1733, 1651, 1509, 1439, 1207, 1097, 1009, 976 cm^−1^; HRMS (+ESI): Found *m/z* 762.5160, [M]^2+^, C_44_H_67_N_5_O_6_ required 762.5164.

#### 3.1.24. 1,1′,1″-(((((3R,5S,7R,8R,9S,10S,12S,13R,14S,17R)-17-((R)-5-(((S)-3-(1H-Indol-3-yl)-1-methoxy-1-oxopropan-2-yl)amino)-5-oxopentan-2-yl)-10,13-dimethylhexadecahydro-1H-cyclopenta[α]phenanthrene-3,7,12-triyl)tris(oxy))tris(2-oxoethane-2,1-diyl))tris(azanediyl))tris(3-methyl-1-oxobutan-2-aminium) chloride (**27**)

The title compound was synthesised from **19a** (0.250 g, 0.281 mmol), Boc-Val-OH (0.371 g, 1.687 mmol), EDC.HCl (0.323 g, 1.687 mmol), 1-hydroxybenzotriazole (HOBt) hydrate (0.153 g, 0.843 mmol) and DIPEA (0.25 mL, 1.41 mmol) following general procedure F. The product **27** was obtained as a white solid (0.029 g, 10%); mp 242.9 °C; ^1^H NMR (600 MHz, DMSO-*d*_6_) δ 0.69 (s, 3H, CH_3_), 0.72–0.76 (d, 3H, CH_3_), 0.91 (s, 3H, CH_3_), 1.00 (d, *J* = 8.2 Hz, 18H, 3 × CH_3_-valine side chain), 1.04–3.57 (m, CH, CH_2,_ steroid), 3.12 (d, 2H, -CO-CH-CH_2_), 3.57 (s, 3H, OCH_3_), 3.69 (s, 3H, 3 × -CO-CH-NH_3_), 3.83 (s, 2H, -NH-CH_2_-CO), 3.99–4.07 (m, 4H, 2 × -NH-CH_2_-CO), 4.45–4.49 (m, 1H, -CO-CH-NH), 4.54 (s, 1H, -OCH), 4.85 (s, 1H, -OCH), 5.07 (s, 1H, -OCH), 6.96–7.01 (m, 1H, ArH), 7.04–7.10 (m, 1H, ArH), 7.15 (s, 1H, ArH), 7.35 (d, *J* = 8.0 Hz, 1H, ArH), 7.48 (d, *J* = 7.9 Hz, 1H, ArH), 8.24–8.33 (m, 10H, 3 × NH_3_, NH), 9.01 (s, 1H, NH), 9.03–9.07 (m, 1H, NH), 9.10–9.14 (m, 1H, NH), 10.93 (s, 1H, NH-indole); ^13^C NMR (150 MHz, DMSO-*d*_6_) δ 173.2, 173.1, 169.2, 169.2, 168.9, 168.8, 136.6, 127.5, 124.1, 121.4, 118.9, 118.4, 112.0, 110.0, 76.1, 74.9, 72.0, 65.4, 57.7, 57.6, 53.7, 52.2, 47.5, 45.2, 44.8, 43.6, 41.4, 41.2, 37.3, 34.8, 34.7, 34.6, 34.5, 34.4, 32.6, 31.6, 31.2, 30.3, 30.3, 30.2, 29.8, 28.9, 28.1, 27.5, 27.4, 26.8, 25.8, 22.7, 22.6, 22.5, 18.6, 18.4, 18.2, 17.9, 15.7, 12.5, 12.4; IR (ATR): ν_max_ 3223, 3052, 2936, 2343, 2095, 1730, 1674, 1509, 1378, 1196, 1097, 1004 cm^−1^; HRMS (+ESI): Found *m/z* 359.8915, [M]^3+^, C_57_H_88_N_8_O_12_ required 359.8913.

### 3.2. Minimal Inhibitory Concentration (MIC) Assay

The antimicrobial activities of the synthesised compounds were investigated by determining their minimum inhibitory concentration (MIC) according to a previously published protocol [22]. The bacterial strains used were *S. aureus* 38 (isolated from a human corneal ulcer), *S. aureus* 111 (isolated from a corneal ulcer; resistant to oxacillin, ciprofloxacin, ceftazidime, chloramphenicol, azithromycin and polymyxin B) [23], *S. aureus* 112 (isolated from a corneal ulcer; resistant to oxacillin, ciprofloxacin, ceftazidime, chloram-phenicol, azithromycin and polymyxin B) [23], *E. coli* K12 (ATCC 10798; isolated from faeces) and *P. aeruginosa* PAO1 (isolated from a wound). A single colony of bacteria was cultured overnight in trypticase soy broth (TSB) at 37 °C. Following incubation, the resulting bacterial culture was collected by centrifugation and re-suspended in TSB twice. The optical density (OD) of the resulting culture was adjusted to OD_660_ = 0.1 in TSB (equivalent to 10^8^ colony forming units (CFU)/mL bacteria) and was further diluted to 10^5^ CFU/mL in TSB. Then, 100 μL of the bacterial solution was added to wells of a 96-well plate containing 100 μL serially diluted compound, with final concentration ranging from 1 to 250 μM. The plates were then incubated at 37 °C for 24 h and the data were recorded by measuring the OD value at 660 nm using a FLUOstar Omega microplate reader (BMG Labtech). The MIC value of each compound was determined as the lowest concentration that completely inhibited the growth of bacteria. Each experiment was performed in triplicate and was repeated in two independent experiments.

### 3.3. Tethered Bilayer Lipid Membrane Assay

Tethered bilayer lipid membranes (tBLMs), in association with AC electrical impedance spectroscopy techniques, were employed to measure changes in lipid bilayer conductance and capacitance in the presence of the test compound [30,31]. Using a previously described solvent exchange technique, sparsely tethered tBLMs were prepared using a zwitterionic 1-palmitoyl- 2-oleoyl-glycero-3-phosphocholine (POPC) or a mixture of 30% palmitoyl-oleoyl-phosphatidylglycerol (POPG) lipids with 70% POPC in order to create a negatively charged tBLM. Briefly, pre-prepared tethered benzyl-disulfide (tetra-ethyleneglycol) *n* = 2 C20-phytanyl tethers: benzyl-disulfide-tetra-ethyleneglycol-OH spacers in the ratio of 1:10 were coated onto a gold patterned polycarbonate slide (SDx Tethered Membranes Pty Ltd., Sydney, Australia). Using a specialised cartridge chamber, a 3-mM solution of a standard mobile lipid phase POPC (Avanti Lipids, Alabaster, AL, USA) was added to the tethering chemistries. All lipids were dissolved in 100% ethanol. To create negatively charged membranes, similar to those present in bacterial species, the same mobile lipid phase was instead supplemented with 30% POPG (Avanti Lipids, Alabaster, AL, USA). Lipids were left for 2 min to associate with the tethering chemistries before being washed with 3 × 400 mL of phosphate buffered saline (PBS). AC electrical impedance spectrometry (EIS) was used monitor any changes in membrane conduction and capacitance as a result of adding the tested compound in increasing concentrations. EIS measures employed a 50-mV peak-to-peak AC excitation from 0.1 to 2000 Hz with four steps per decade and were recorded using a TethaPodTM operated with TethaQuickTM software (SDx Tethered Membranes Pty Ltd., Sydney, Australia). The data were fitted to an equivalent circuit using a proprietary adaptation of a Lev Mar fitting routine. This circuit consists of a Constant Phase Element (CPE), to represent the imperfect capacitance of the gold tethering electrode, in series with a Resistor/Capacitor network to represent the lipid bilayer membrane [30].

### 3.4. Cytoplasmic Membrane Depolarisation

The method was adopted from Wu et al. [34] with slight modification. Bacterial cytoplasmic membrane permeability was determined using membrane potential sensitive dye diSC3-5 (3,3′-dipropylthiadicarbocyanine iodide), which penetrates inside bacterial cells depending on the membrane potential gradient of the cytoplasmic membrane. Bacteria were grown in Mueller Hinton Broth (MHB) to mid-log phase by incubating with shaking at 37 °C for 18–24 h. Following incubation, bacteria were washed with 5 mM HEPES containing 20 mM glucose pH 7.2 and resuspended in the same buffer to an OD_600_ of 0.05–0.06, which gave 1 × 10^7^ CFU/mL. The dye diSC3-5 was added at 4 μM to the bacterial suspension. The suspensions were incubated at room temperature for 1 h in darkness for maximum dye take-up by the bacterial cells. Then, 100 mM KCl was added to balance the K+ outside and inside the bacterial cell to prevent further uptake or outflow of the dye. Next, 100 μL of bacterial suspension was added into a 96-well microtiter plate with equal volume of antimicrobial compounds. DMSO (20%) was set as a positive control while dye and only bacterial cells were set as negative control. Fluorescence was measured with a luminescence spectrophotometer at 3 min intervals at an excitation wavelength of 621 m and an emission wavelength of 670 nm.

### 3.5. Cell Viability Count

The number of viable cells was confirmed by serially diluting aliquots of bacteria in D/E neutralizing broth (Remel, Lenexa, KS, USA) and plating onto Tryptic Soy Agar (Oxoid, Basingstoke, UK) containing phosphatidylcholine (0.7 g/L) and Tween 80 (5 mL/L). The plates were incubated at 37 °C overnight and numbers of live bacteria were enumerated and expressed as CFU/mL. The experiment was performed in triplicate.

### 3.6. Toxicity Assay

The cell viability of measurement of the compounds was performed using an MTT (3-(4,5-dimethylthiazol-2-yl)-2,5-diphenyltetrazolium bromide) (Sigma Aldrich, St Louis, MO, USA) colorimetric assay with normal human lung fibroblasts MRC-5 cell. The cells were cultured in minimal essential medium (MEM, Invitrogen) and 10% FBS (Foetal Bovine Serum). The cell line was maintained at 37 °C in 5% CO_2_ as an adherent monolayer and was passaged upon reaching confluence by standard cell culture techniques. MRC-5 cells were seeded at 5000 cells/well in 96-well plates to ensure full confluence (quiescence) and left overnight to adhere to the plate wells. The adherent cells were treated with 1–500 μM of compounds by dissolving in DMSO and serially diluting with media. The final concentration of DMSO was 0.5% (*v*/*v*). After 72 h drug incubation, 20 mL stock MTT solution (5 mg/mL) was added and the cells were incubated for another 3.5–4 h. The MTT solution containing media was carefully aspirated without displacing the purple crystals from the bottom, and 80 mL acid-isopropanol was added to all wells and mixed slowly by shaking with orbital shaker to dissolve the dark blue crystals. The metabolic activity was detected by spectrophotometric analysis by assessing the read absorbance (590/620 nm) on EnSight plate reader (PerkinElmer, Waltham, MA, USA), and cell viability was expressed as a percentage of untreated control cells. The determination of IC_50_ values was performed using GraphPad Prism (GraphPad, San Diego, CA, USA). Each experiment was performed in triplicate.

## 4. Conclusions

In this study, amphiphilicity of novel small molecular AMP mimics was conferred by functionalising the four cholic acid derivatives with the addition of a tryptophan moiety and different cationic groups at the carboxyl terminal and the hydroxyl ends, respectively. The difference in the number of the hydroxyl groups present on the starting cholic acid scaffold allowed the preparation of mono-, di- and tri-substituted analogues with positively charged amino acids. The significance of the hydrophobic tryptophan moiety was further investigated by its replacement with two related aromatic systems. Overall, 18 novel SMAMPs were synthesised, and their antimicrobial activities were evaluated against both Gram-positive *S. aureus* and Gram-negative *E. coli*. In vitro biological studies identified that a minimal positive net charge of +2 was essential for observable antimicrobial activity. The presence of the hydrophobic tryptophan moiety enhanced the antibacterial potency against Gram-positive *S. aureus*, but was found to be non-essential for activity against Gram-negative *E. coli*. Notably, all di-substituted analogues possessed high antimicrobial activity against both Gram-positive *S. aureus* and Gram-negative *E. coli,* with analogues **17c** and **17d** containing lysine and ornithine residues, respectively, showing the highest antibacterial potency with MIC values of 4 μM. Furthermore, analogue **17d** showed a moderate selectivity ratio against *S. aureus* over mammalian cells of 8.32. Glycine-valine dipeptide derivative **27** exhibited the highest antibacterial activity against Gram-negative *E. coli*, with an MIC of 8 μM. Furthermore, these compounds were identified as able to act via the disruption of bacterial cell membrane, similar to most AMPs, as evidenced by the observations made in negatively charged tBLMs and the dye release assay studies.

## Figures and Tables

**Table 1:** Minimum inhibitory concentration of compounds against *S. aureus* and *E. coli*. (ND = Not determined).

Compound				MIC (μM)				
	Scaffold	Cationic Group	Carboxyl Terminal Moiety	*S. Aureus*	*S. Aureus*	*S. Aureus*	*E. Coli*	*P. Aeruginosa* (PAO1)
				(SA38)	*(SA111)*	*(SA112)*	*(K12)*	
16a	LICA	Gly	Trp OMe	>125	ND	ND	>125	>125
16b	LICA	GABA	Trp OMe	>125	ND	ND	>125	>125
16c	LICA	Orn	Trp OMe	8	8	4	>125	>125
16d	LICA	Lys	Trp OMe	4	8	8	>125	>125
17a	CHCA	Gly	Trp OMe	8	8	8	16	>125
17b	CHCA	GABA	Trp OMe	16	ND	ND	4	16
17c	CHCA	Orn	Trp OMe	4	4	4	16	8
17d	CHCA	Lys	Trp OMe	4	4	4	16	8
18a	DCA	Gly	Trp OMe	8	8	8	16	>125
19a	CA	Gly	Trp OMe	31	ND	ND	31	>125
19b	CA	GABA	Trp OMe	16	ND	ND	16	31
19c	CA	Orn	Trp OMe	16	ND	ND	4	16
19d	CA	Lys	Trp OMe	16	ND	ND	31	16
20	CHCA	Orn	Phe OMe	8	8	8	16	31
21	CHCA	Orn	NHBn	8	8	8	16	31
23	LICA	Guanidine–GABA	Trp OMe	16	ND	ND	>125	>125
25	LICA	Gly–Lys	Trp OMe	8	4	4	>125	>125
27	CA	Gly–Val	Trp OMe	16	ND	ND	8	63

**Table 2:** IC_50_ value of compounds against MRC-5 normal human lung fibroblasts and their therapeutic indices with respect to *S. aureus*.

Compound	IC_50_ (µM)	MIC against *S. Aureus* (µM)	Therapeutic Window
16d	4.79	4	1.24
17d	33.3	4	8.32

## Data Availability

Data is contained within the article and Supplementary Materials.

## Supplementary Materials

The following supporting information can be downloaded at https://www.mdpi.com/article/10.3390/ijms23094623/s1.
