# Supplementary material for: Cholic Acid-Based Antimicrobial Peptide Mimics as Antibacterial Agents
Source: Int J Mol Sci. 2022 Apr 21;23(9):4623. doi: 10.3390/ijms23094623 (PMC9101178; doi:10.3390/ijms23094623)

Supporting information

# Cholic acid-based antimicrobial peptide mimics as antibacterial agents

**Jie Wu [1,†], Tsz Tin Yu [1,†], Rajesh Kuppusamy [1,2], Md. Musfizur Hassan [1], Amani Alghalayini [3], Charles G. Cranfield [3], Mark D. P. Willcox [2], David StC. Black [1,*] and Naresh Kumar [1,*]**

[1] School of Chemistry, The University of New South Wales, Sydney, NSW 2052, Australia
[2] School of Optometry and Vision Science, University of New South Wales, Sydney, NSW 2052, Australia
[3] School of Life Sciences, University of Technology Sydney, PO Box 123, Ultimo 2007, Australia
* Correspondence: d.black@unsw.edu.au (D.StC.B.); n.kumar@unsw.edu.au (N.K.)
† Co-first author

## Contents

$^1$H NMR and $^{13}$C NMR spectra of synthesised compounds ....... 5
Acquisition parameters for $^1$H NMR and $^{13}$C NMR spectra ....... 5
2-(((3*R*,5*R*,8*R*,9*S*,10*S*,13*R*,14*S*,17*R*)-17-((*R*)-5-(((*S*)-3-(1*H*-Indol-3-yl)-1-methoxy-1-oxopropan-2-yl)amino)-5-oxopentan-2-yl)-10,13-dimethylhexadecahydro-1*H*-cyclopenta[*a*]phenanthren-3-yl)oxy)-2-oxoethan-1-aminium (16a) ....... 6
4-(((3*R*,5*R*,8*R*,9*S*,10*S*,13*R*,14*S*,17*R*)-17-((*R*)-5-(((*S*)-3-(1*H*-Indol-3-yl)-1-methoxy-1-oxopropan-2-yl)amino)-5-oxopentan-2-yl)-10,13-dimethylhexadecahydro-1*H*-cyclopenta[*a*]phenanthren-3-yl)oxy)-4-oxobutan-1-aminium (16b) ....... 7
(*S*)-5-(((3*R*,5*R*,8*R*,9*S*,10*S*,13*R*,14*S*,17*R*)-17-((*R*)-5-(((*S*)-3-(1*H*-Indol-3-yl)-1-methoxy-1-oxopropan-2-yl)amino)-5-oxopentan-2-yl)-10,13-dimethylhexadecahydro-1*H*-cyclopenta[*a*]phenanthren-3-yl)oxy)-5-oxopentane-1,4-diaminium (16c) ....... 8
(*S*)-6-(((3*R*,5*R*,8*R*,9*S*,10*S*,13*R*,14*S*,17*R*)-17-((*R*)-5-(((*S*)-3-(1*H*-Indol-3-yl)-1-methoxy-1-oxopropan-2-yl)amino)-5-oxopentan-2-yl)-10,13-dimethylhexadecahydro-1*H*-cyclopenta[*a*]phenanthren-3-yl)oxy)-6-oxohexane-1,5-diaminium (16d) ....... 9
2,2'-(((3*R*,5*S*,7*R*,8*R*,9*S*,10*S*,13*R*,14*S*,17*R*)-17-((*R*)-5-(((*S*)-3-(1*H*-Indol-3-yl)-1-methoxy-1-oxopropan-2-yl)amino)-5-oxopentan-2-yl)-10,13-dimethylhexadecahydro-1*H*-cyclopenta[*a*]phenanthrene-3,7-diyl)bis(oxy))bis(2-oxoethan-1-aminium) (17a) ....... 10
4,4'-(((3*R*,5*S*,7*R*,8*R*,9*S*,10*S*,13*R*,14*S*,17*R*)-17-((*R*)-5-(((*S*)-3-(1*H*-Indol-3-yl)-1-methoxy-1-oxopropan-2-yl)amino)-5-oxopentan-2-yl)-10,13-dimethylhexadecahydro-1*H*-cyclopenta[*a*]phenanthrene-3,7-diyl)bis(oxy))bis(4-oxobutan-1-aminium) (17b) ....... 11
(4*S*,4'*S*)-5,5'-(((3*R*,5*S*,7*R*,8*R*,9*S*,10*S*,13*R*,14*S*,17*R*)-17-((*R*)-5-(((*S*)-3-(1*H*-Indol-3-yl)-1-methoxy-1-oxopropan-2-yl)amino)-5-oxopentan-2-yl)-10,13-dimethylhexadecahydro-1*H*-cyclopenta[*a*]phenanthrene-3,7-diyl)bis(oxy))bis(5-oxopentane-1,4-diaminium) (17c) ....... 12
(5*S*,5'*S*)-6,6'-(((3*R*,5*S*,7*R*,8*R*,9*S*,10*S*,13*R*,14*S*,17*R*)-17-((*R*)-5-(((*S*)-3-(1*H*-Indol-3-yl)-1-methoxy-1-oxopropan-2-yl)amino)-5-oxopentan-2-yl)-10,13-dimethylhexadecahydro-1*H*-cyclopenta[*a*]phenanthrene-3,7-diyl)bis(oxy))bis(6-oxohexane-1,5-diaminium) (17d) ....... 13

2,2'-(((3*R*,5*R*,8*R*,9*S*,10*S*,12*S*,13*R*,14*S*,17*R*)-17-((*R*)-5-(((*S*)-3-(1*H*-Indol-3-yl)-1-methoxy-1-oxopropan-2-yl)amino)-5-oxopentan-2-yl)-10,13-dimethylhexadecahydro-1*H*-cyclopenta[*a*]phenanthrene-3,12-diyl)bis(oxy))bis(2-oxoethan-1-aminium) (18a) ....................... 14
2,2'-(((3*R*,5*S*,7*R*,8*R*,9*S*,10*S*,12*S*,13*R*,14*S*,17*R*)-17-((*R*)-5-(((*S*)-3-(1*H*-Indol-3-yl)-1-methoxy-1-oxopropan-2-yl)amino)-5-oxopentan-2-yl)-3-(2-aminoacetoxy)-10,13-dimethylhexadecahydro-1*H*-cyclopenta[*a*]phenanthrene-7,12-diyl)bis(oxy))bis(2-oxoethan-1-aminium) (19a) ................. 15
4,4',4''-(((3*R*,5*S*,7*R*,8*R*,9*S*,10*S*,12*S*,13*R*,14*S*,17*R*)-17-((*R*)-5-(((*S*)-3-(1*H*-Indol-3-yl)-1-methoxy-1-oxopropan-2-yl)amino)-5-oxopentan-2-yl)-10,13-dimethylhexadecahydro-1*H*-cyclopenta[*a*]phenanthrene-3,7,12-triyl)tris(oxy))tris(4-oxobutan-1-aminium) (19b) .................. 16
(4*S*,4'*S*,4''*S*)-5,5',5''-(((3*R*,5*S*,7*R*,8*R*,9*S*,10*S*,12*S*,13*R*,14*S*,17*R*)-17-((*R*)-5-(((*S*)-3-(1*H*-Indol-3-yl)-1-methoxy-1-oxopropan-2-yl)amino)-5-oxopentan-2-yl)-10,13-dimethylhexadecahydro-1*H*-cyclopenta[*a*]phenanthrene-3,7,12-triyl)tris(oxy))tris(5-oxopentane-1,4-diaminium) (19c) ......... 17
(5*S*,5'*S*,5''*S*)-6,6',6''-(((3*R*,5*S*,7*R*,8*R*,9*S*,10*S*,12*S*,13*R*,14*S*,17*R*)-17-((*R*)-5-(((*S*)-3-(1*H*-Indol-3-yl)-1-methoxy-1-oxopropan-2-yl)amino)-5-oxopentan-2-yl)-10,13-dimethylhexadecahydro-1*H*-cyclopenta[*a*]phenanthrene-3,7,12-triyl)tris(oxy))tris(6-oxohexane-1,5-diaminium) (19d) .......... 18
(4*S*,4'*S*)-5,5'-(((3*R*,5*S*,7*R*,8*R*,9*S*,10*S*,13*R*,14*S*,17*R*)-17-((*R*)-5-(((*S*)-1-Methoxy-1-oxo-3-phenylpropan-2-yl)amino)-5-oxopentan-2-yl)-10,13-dimethylhexadecahydro-1*H*-cyclopenta[*a*]phenanthrene-3,7-diyl)bis(oxy))bis(5-oxopentane-1,4-diaminium) (20) ................. 19
(4*S*,4'*S*)-5,5'-(((3*R*,5*S*,7*R*,8*R*,9*S*,10*S*,13*R*,14*S*,17*R*)-17-((*R*)-5-(Benzylamino)-5-oxopentan-2-yl)-10,13-dimethylhexadecahydro-1*H*-cyclopenta[*a*]phenanthrene-3,7-diyl)bis(oxy))bis(5-oxopentane-1,4-diaminium) (21) ........................................................................................ 19
((4-(((3*R*,5*R*,8*R*,9*S*,10*S*,13*R*,14*S*,17*R*)-17-((*R*)-5-(((*S*)-3-(1*H*-Indol-3-yl)-1-methoxy-1-oxopropan-2-yl)amino)-5-oxopentan-2-yl)-10,13-dimethylhexadecahydro-1*H*-cyclopenta[*a*]phenanthren-3-yl)oxy)-4-oxobutyl)amino)(amino)methaniminium (23)............................................................ 20
Methyl ((*R*)-4-((3*R*,5*R*,8*R*,9*S*,10*S*,13*R*,14*S*,17*R*)-3-((L-lysylglycyl)oxy)-10,13-dimethylhexadecahydro-1*H*-cyclopenta[*a*]phenanthren-17-yl)pentanoyl)-L-tryptophanate (25) .. 21
1,1',1''-(((((3*R*,5*S*,7*R*,8*R*,9*S*,10*S*,12*S*,13*R*,14*S*,17*R*)-17-((*R*)-5-(((*S*)-3-(1*H*-Indol-3-yl)-1-methoxy-1-oxopropan-2-yl)amino)-5-oxopentan-2-yl)-10,13-dimethylhexadecahydro-1*H*-cyclopenta[*a*]phenanthrene-3,7,12-triyl)tris(oxy))tris(2-oxoethane-2,1-diyl))tris(azanediyl))tris(3-methyl-1-oxobutan-2-aminium) (27) ..................................................................................... 22
High resolution mass spectra (HRMS) of synthesised compounds ...................................................... 23
2-(((3*R*,5*R*,8*R*,9*S*,10*S*,13*R*,14*S*,17*R*)-17-((*R*)-5-(((*S*)-3-(1*H*-Indol-3-yl)-1-methoxy-1-oxopropan-2-yl)amino)-5-oxopentan-2-yl)-10,13-dimethylhexadecahydro-1*H*-cyclopenta[*a*]phenanthren-3-yl)oxy)-2-oxoethan-1-aminium (16a) .......................................................................................... 23
4-(((3*R*,5*R*,8*R*,9*S*,10*S*,13*R*,14*S*,17*R*)-17-((*R*)-5-(((*S*)-3-(1*H*-Indol-3-yl)-1-methoxy-1-oxopropan-2-yl)amino)-5-oxopentan-2-yl)-10,13-dimethylhexadecahydro-1*H*-cyclopenta[*a*]phenanthren-3-yl)oxy)-4-oxobutan-1-aminium (16b)........................................................................................ 25
(*S*)-5-(((3*R*,5*R*,8*R*,9*S*,10*S*,13*R*,14*S*,17*R*)-17-((*R*)-5-(((*S*)-3-(1*H*-Indol-3-yl)-1-methoxy-1-oxopropan-2-yl)amino)-5-oxopentan-2-yl)-10,13-dimethylhexadecahydro-1*H*-cyclopenta[*a*]phenanthren-3-yl)oxy)-5-oxopentane-1,4-diaminium (16c) .................................... 27

(*S*)-6-(((3*R*,5*R*,8*R*,9*S*,10*S*,13*R*,14*S*,17*R*)-17-((*R*)-5-(((*S*)-3-(1*H*-Indol-3-yl)-1-methoxy-1-oxopropan-2-yl)amino)-5-oxopentan-2-yl)-10,13-dimethylhexadecahydro-1*H*-cyclopenta[*a*]phenanthren-3-yl)oxy)-6-oxohexane-1,5-diaminium (16d) .................................... 29
2,2'-(((3*R*,5*S*,7*R*,8*R*,9*S*,10*S*,13*R*,14*S*,17*R*)-17-((*R*)-5-(((*S*)-3-(1*H*-Indol-3-yl)-1-methoxy-1-oxopropan-2-yl)amino)-5-oxopentan-2-yl)-10,13-dimethylhexadecahydro-1*H*-cyclopenta[*a*]phenanthrene-3,7-diyl)bis(oxy))bis(2-oxoethan-1-aminium) (17a) ........................ 31
4,4'-(((3*R*,5*S*,7*R*,8*R*,9*S*,10*S*,13*R*,14*S*,17*R*)-17-((*R*)-5-(((*S*)-3-(1*H*-Indol-3-yl)-1-methoxy-1-oxopropan-2-yl)amino)-5-oxopentan-2-yl)-10,13-dimethylhexadecahydro-1*H*-cyclopenta[*a*]phenanthrene-3,7-diyl)bis(oxy))bis(4-oxobutan-1-aminium) (17b) ........................ 33
(4*S*,4'*S*)-5,5'-(((3*R*,5*S*,7*R*,8*R*,9*S*,10*S*,13*R*,14*S*,17*R*)-17-((*R*)-5-(((*S*)-3-(1*H*-Indol-3-yl)-1-methoxy-1-oxopropan-2-yl)amino)-5-oxopentan-2-yl)-10,13-dimethylhexadecahydro-1*H*-cyclopenta[*a*]phenanthrene-3,7-diyl)bis(oxy))bis(5-oxopentane-1,4-diaminium) (17c) ............... 35
(5*S*,5'*S*)-6,6'-(((3*R*,5*S*,7*R*,8*R*,9*S*,10*S*,13*R*,14*S*,17*R*)-17-((*R*)-5-(((*S*)-3-(1*H*-Indol-3-yl)-1-methoxy-1-oxopropan-2-yl)amino)-5-oxopentan-2-yl)-10,13-dimethylhexadecahydro-1*H*-cyclopenta[*a*]phenanthrene-3,7-diyl)bis(oxy))bis(6-oxohexane-1,5-diaminium) (17d) ............... 37
2,2'-(((3*R*,5*R*,8*R*,9*S*,10*S*,12*S*,13*R*,14*S*,17*R*)-17-((*R*)-5-(((*S*)-3-(1*H*-Indol-3-yl)-1-methoxy-1-oxopropan-2-yl)amino)-5-oxopentan-2-yl)-10,13-dimethylhexadecahydro-1*H*-cyclopenta[*a*]phenanthrene-3,12-diyl)bis(oxy))bis(2-oxoethan-1-aminium) (18a) ...................... 39
2,2'-(((3*R*,5*S*,7*R*,8*R*,9*S*,10*S*,12*S*,13*R*,14*S*,17*R*)-17-((*R*)-5-(((*S*)-3-(1*H*-Indol-3-yl)-1-methoxy-1-oxopropan-2-yl)amino)-5-oxopentan-2-yl)-3-(2-aminoacetoxy)-10,13-dimethylhexadecahydro-1*H*-cyclopenta[*a*]phenanthrene-7,12-diyl)bis(oxy))bis(2-oxoethan-1-aminium) (19a) ................ 41
4,4',4''-(((3*R*,5*S*,7*R*,8*R*,9*S*,10*S*,12*S*,13*R*,14*S*,17*R*)-17-((*R*)-5-(((*S*)-3-(1*H*-Indol-3-yl)-1-methoxy-1-oxopropan-2-yl)amino)-5-oxopentan-2-yl)-10,13-dimethylhexadecahydro-1*H*-cyclopenta[*a*]phenanthrene-3,7,12-triyl)tris(oxy))tris(4-oxobutan-1-aminium) (19b) .................. 43
(4*S*,4'*S*,4''*S*)-5,5',5''-(((3*R*,5*S*,7*R*,8*R*,9*S*,10*S*,12*S*,13*R*,14*S*,17*R*)-17-((*R*)-5-(((*S*)-3-(1*H*-Indol-3-yl)-1-methoxy-1-oxopropan-2-yl)amino)-5-oxopentan-2-yl)-10,13-dimethylhexadecahydro-1*H*-cyclopenta[*a*]phenanthrene-3,7,12-triyl)tris(oxy))tris(5-oxopentane-1,4-diaminium) (19c) ......... 45
(5*S*,5'*S*,5''*S*)-6,6',6''-(((3*R*,5*S*,7*R*,8*R*,9*S*,10*S*,12*S*,13*R*,14*S*,17*R*)-17-((*R*)-5-(((*S*)-3-(1*H*-Indol-3-yl)-1-methoxy-1-oxopropan-2-yl)amino)-5-oxopentan-2-yl)-10,13-dimethylhexadecahydro-1*H*-cyclopenta[*a*]phenanthrene-3,7,12-triyl)tris(oxy))tris(6-oxohexane-1,5-diaminium) (19d) .......... 47
(4*S*,4'*S*)-5,5'-(((3*R*,5*S*,7*R*,8*R*,9*S*,10*S*,13*R*,14*S*,17*R*)-17-((*R*)-5-(((*S*)-1-Methoxy-1-oxo-3-phenylpropan-2-yl)amino)-5-oxopentan-2-yl)-10,13-dimethylhexadecahydro-1*H*-cyclopenta[*a*]phenanthrene-3,7-diyl)bis(oxy))bis(5-oxopentane-1,4-diaminium) (20) ................ 49
(4*S*,4'*S*)-5,5'-(((3*R*,5*S*,7*R*,8*R*,9*S*,10*S*,13*R*,14*S*,17*R*)-17-((*R*)-5-(Benzylamino)-5-oxopentan-2-yl)-10,13-dimethylhexadecahydro-1*H*-cyclopenta[*a*]phenanthrene-3,7-diyl)bis(oxy))bis(5-oxopentane-1,4-diaminium) (21) ........................................................................................ 51
((4-(((3*R*,5*R*,8*R*,9*S*,10*S*,13*R*,14*S*,17*R*)-17-((*R*)-5-(((*S*)-3-(1*H*-Indol-3-yl)-1-methoxy-1-oxopropan-2-yl)amino)-5-oxopentan-2-yl)-10,13-dimethylhexadecahydro-1*H*-cyclopenta[*a*]phenanthren-3-yl)oxy)-4-oxobutyl)amino)(amino)methaniminium (23) ............................................................. 53

Methyl ((*R*)-4-((3*R*,5*R*,8*R*,9*S*,10*S*,13*R*,14*S*,17*R*)-3-((L-lysylglycyl)oxy)-10,13-dimethylhexadecahydro-1*H*-cyclopenta[*a*]phenanthren-17-yl)pentanoyl)-L-tryptophanate (25)..55
1,1',1''-(((((3*R*,5*S*,7*R*,8*R*,9*S*,10*S*,12*S*,13*R*,14*S*,17*R*)-17-((*R*)-5-(((*S*)-3-(1*H*-Indol-3-yl)-1-methoxy-1-oxopropan-2-yl)amino)-5-oxopentan-2-yl)-10,13-dimethylhexadecahydro-1*H*-cyclopenta[*a*]phenanthrene-3,7,12-triyl)tris(oxy))tris(2-oxoethane-2,1-diyl))tris(azanediyl))tris(3-methyl-1-oxobutan-2-aminium) (27) ..........57

# $^{1}$H NMR and $^{13}$C NMR spectra of synthesised compounds

Acquisition parameters for $^{1}$H NMR and $^{13}$C NMR spectra

$^{1}$H NMR:

Standard Bruker pulse program "zg" was used and the delay (D1) was set to 5 s.

| | | |
|---|---|---|
| **Experiment** | | |
| PULPROG | zg | Current pulse program |
| AQ_mod | DQD | Acquisition mode |
| TD | 32768 | Size of fid |
| DS | 0 | Number of dummy scans |
| NS | 8 | Number of scans |
| TD0 | 1 | Loop count for 'td0' |
| **Width** | | |
| SW [ppm] | 15.9778 | Spectral width |
| SWH [Hz] | 6393.862 | Spectral width |
| AQ [sec] | 2.5624576 | Acquisition time |
| FIDRES [Hz] | 0.390250 | Fid resolution |
| FW [Hz] | 4032000.000 | Filter width |
| **Nucleus 1** | | |
| NUC1 | 1H | Observe nucleus |
| O1 [Hz] | 2601.10 | Transmitter frequency offset |
| O1P [ppm] | 6.500 | Transmitter frequency offset |
| SFO1 [MHz] | 400.1726011 | Transmitter frequency |
| BF1 [MHz] | 400.1700000 | Basic transmitter frequency |

$^{13}$C{$^{1}$H} NMR:

Standard Bruker pulse program "zgpg30" was used and the delay (D1) was set to 1 s.

| | | |
|---|---|---|
| **Experiment** | | |
| PULPROG | zgpg30 | Current pulse program |
| AQ_mod | DQD | Acquisition mode |
| TD | 65536 | Size of fid |
| DS | 2 | Number of dummy scans |
| NS | 256 | Number of scans |
| TD0 | 1 | Loop count for 'td0' |
| **Width** | | |
| SW [ppm] | 238.8728 | Spectral width |
| SWH [Hz] | 24038.461 | Spectral width |
| AQ [sec] | 1.3631488 | Acquisition time |
| FIDRES [Hz] | 0.733596 | Fid resolution |
| FW [Hz] | 4032000.000 | Filter width |
| **Nucleus 1** | | |
| NUC1 | 13C | Observe nucleus |
| O1 [Hz] | 10061.66 | Transmitter frequency offset |
| O1P [ppm] | 99.994 | Transmitter frequency offset |
| SFO1 [MHz] | 100.6328882 | Transmitter frequency |
| BF1 [MHz] | 100.6228265 | Basic transmitter frequency |
| **Nucleus 2** | | |
| NUC2 | 1H | 2nd nucleus |
| O2 [Hz] | 1600.68 | Frequency offset of 2nd nucleus |
| O2P [ppm] | 4.000 | Frequency offset of 2nd nucleus |
| SFO2 [MHz] | 400.1716007 | Frequency of 2nd nucleus |
| BF2 [MHz] | 400.1700000 | Basic frequency of 2nd nucleus |

2-(((3*R*,5*R*,8*R*,9*S*,10*S*,13*R*,14*S*,17*R*)-17-((*R*)-5-(((*S*)-3-(1*H*-Indol-3-yl)-1-methoxy-1-oxopropan-2-yl)amino)-5-oxopentan-2-yl)-10,13-dimethylhexadecahydro-1*H*-cyclopenta[*a*]phenanthren-3-yl)oxy)-2-oxoethan-1-aminium **(16a)**

$^1$H NMR (400 MHz, DMSO-$d_6$):

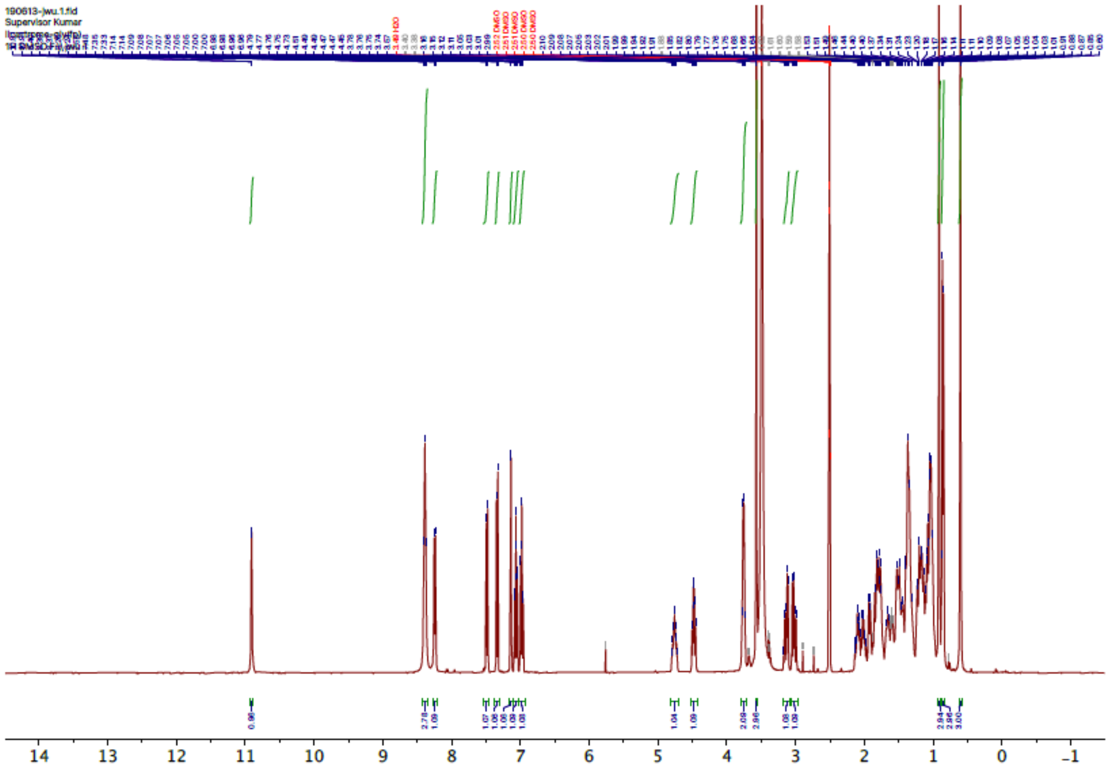

$^{13}$C NMR (100 MHz, DMSO-$d_6$):

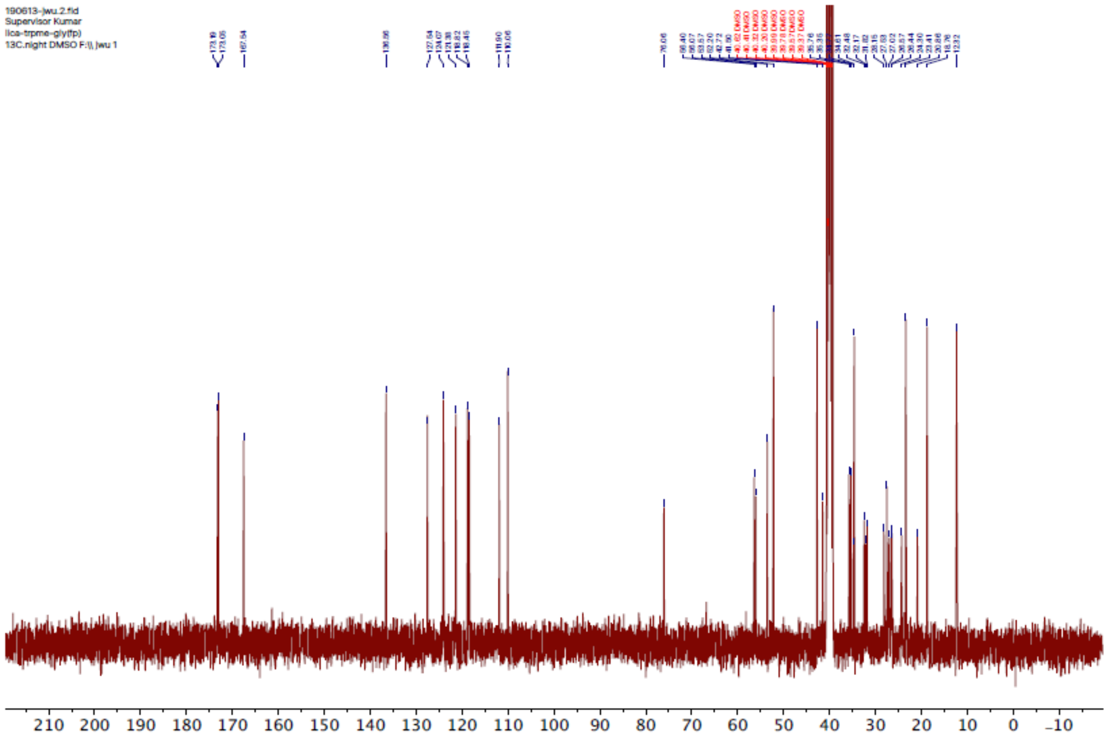

4-(((3*R*,5*R*,8*R*,9*S*,10*S*,13*R*,14*S*,17*R*)-17-((*R*)-5-(((*S*)-3-(1*H*-Indol-3-yl)-1-methoxy-1-oxopropan-2-yl)amino)-5-oxopentan-2-yl)-10,13-dimethylhexadecahydro-1*H*-cyclopenta[*a*]phenanthren-3-yl)oxy)-4-oxobutan-1-aminium **(16b)**

$^{1}$H NMR (400 MHz, DMSO-$d_6$):

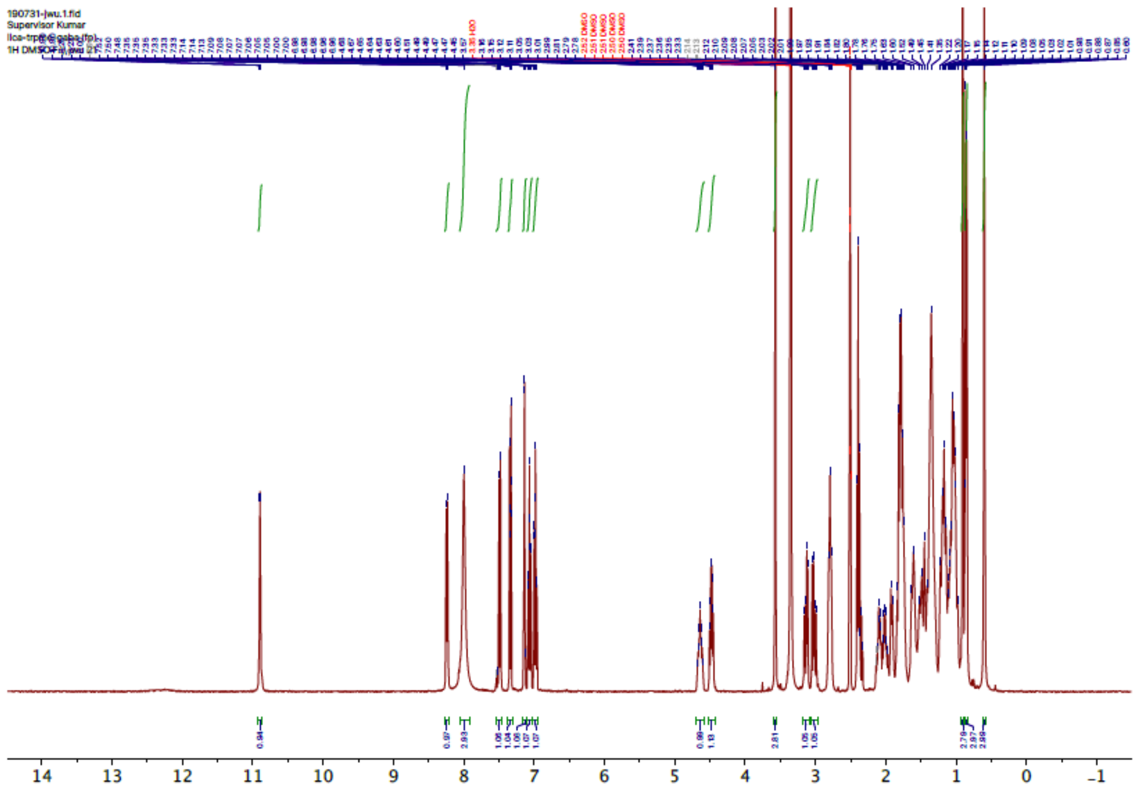

$^{13}$C NMR (100 MHz, DMSO-$d_6$):

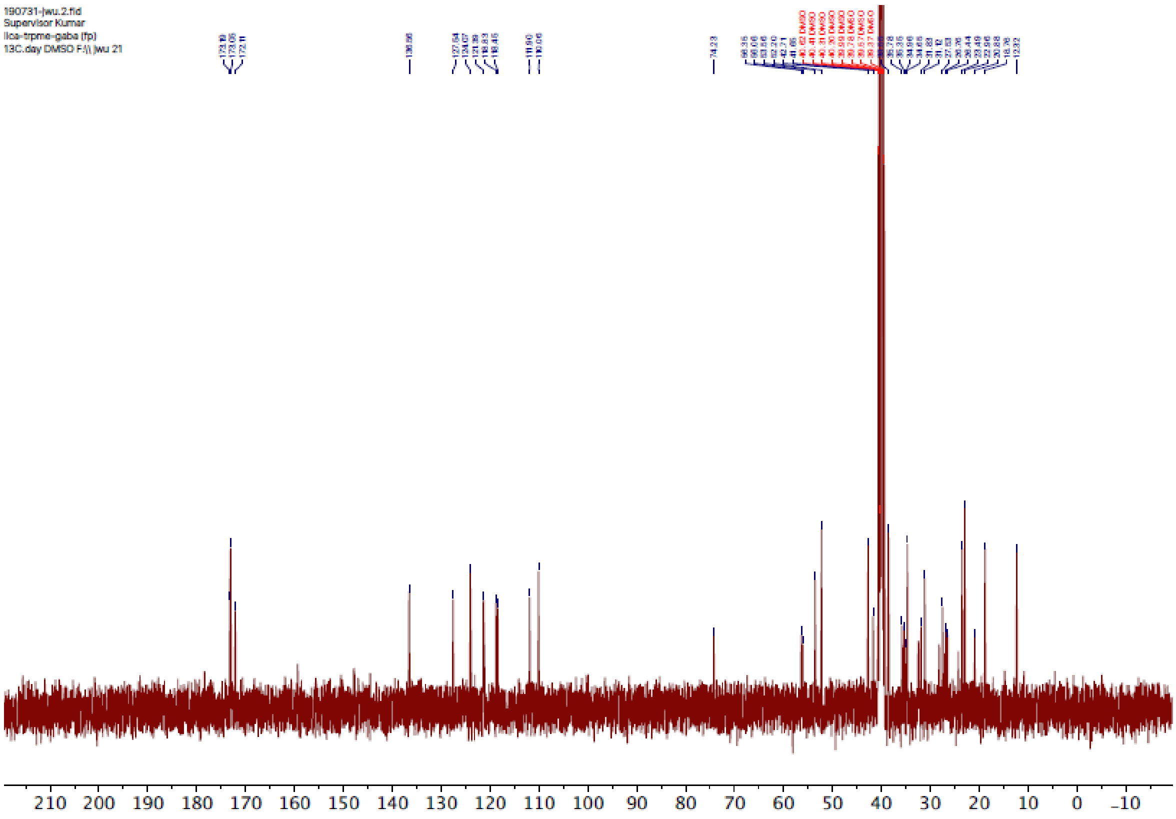

(*S*)-5-(((3*R*,5*R*,8*R*,9*S*,10*S*,13*R*,14*S*,17*R*)-17-((*R*)-5-(((*S*)-3-(1*H*-Indol-3-yl)-1-methoxy-1-oxopropan-2-yl)amino)-5-oxopentan-2-yl)-10,13-dimethylhexadecahydro-1*H*-cyclopenta[*a*]phenanthren-3-yl)oxy)-5-oxopentane-1,4-diaminium **(16c)**

$^{1}$H NMR (400 MHz, DMSO-$d_6$):

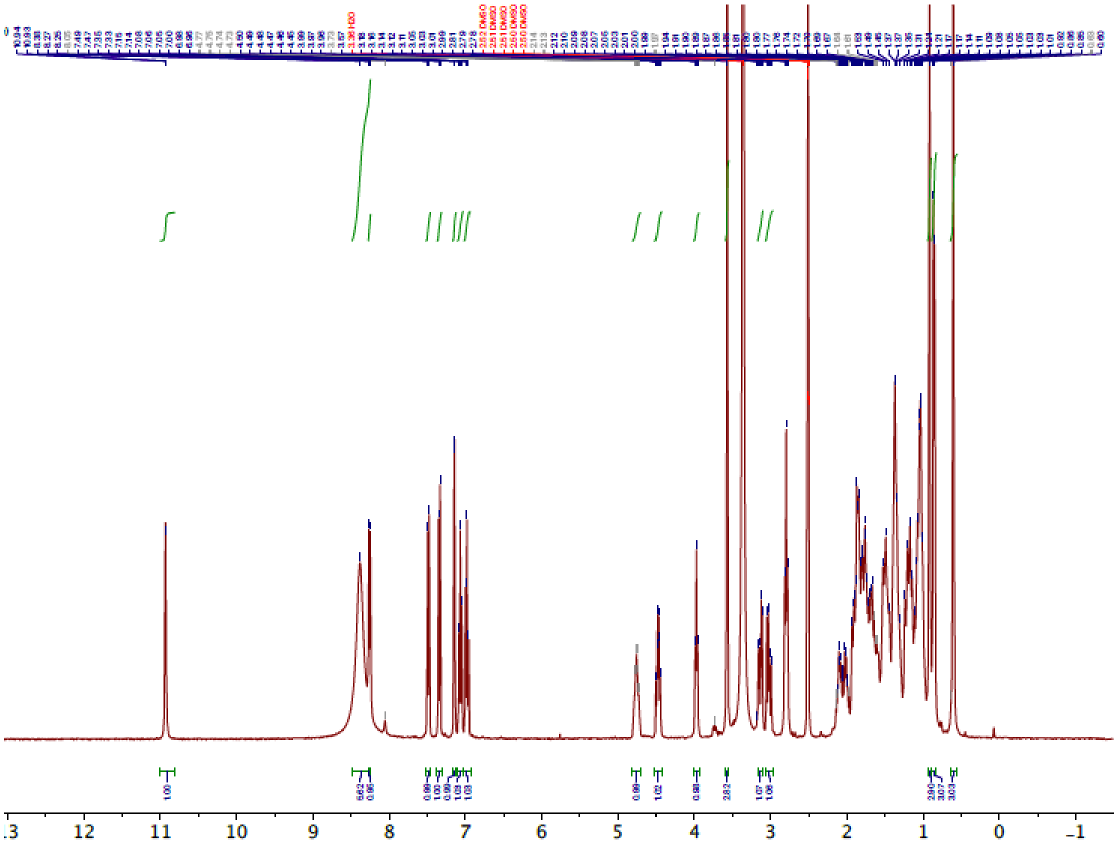


$^{13}$C NMR (100 MHz, DMSO-$d_6$):

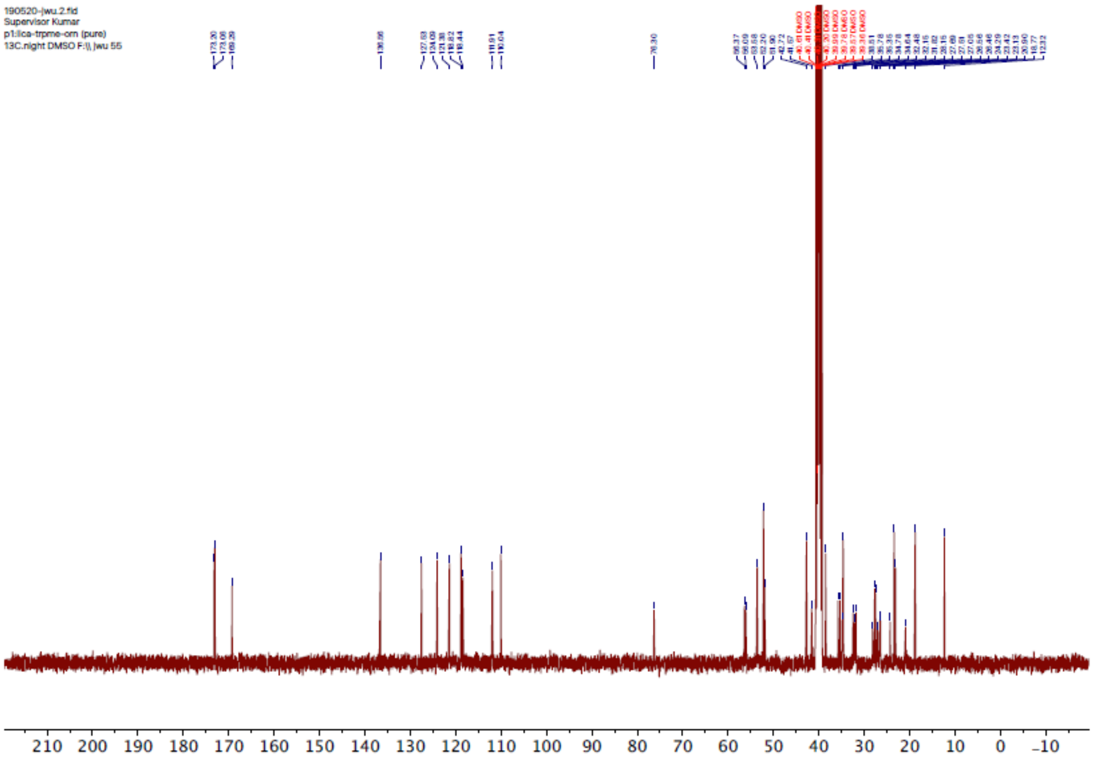

($S$)-6-(((3$R$,5$R$,8$R$,9$S$,10$S$,13$R$,14$S$,17$R$)-17-(($R$)-5-((($S$)-3-(1$H$-Indol-3-yl)-1-methoxy-1-oxopropan-2-yl)amino)-5-oxopentan-2-yl)-10,13-dimethylhexadecahydro-1$H$-cyclopenta[$a$]phenanthren-3-yl)oxy)-6-oxohexane-1,5-diaminium **(16d)**

$^{1}$H NMR (400 MHz, DMSO-$d_6$):

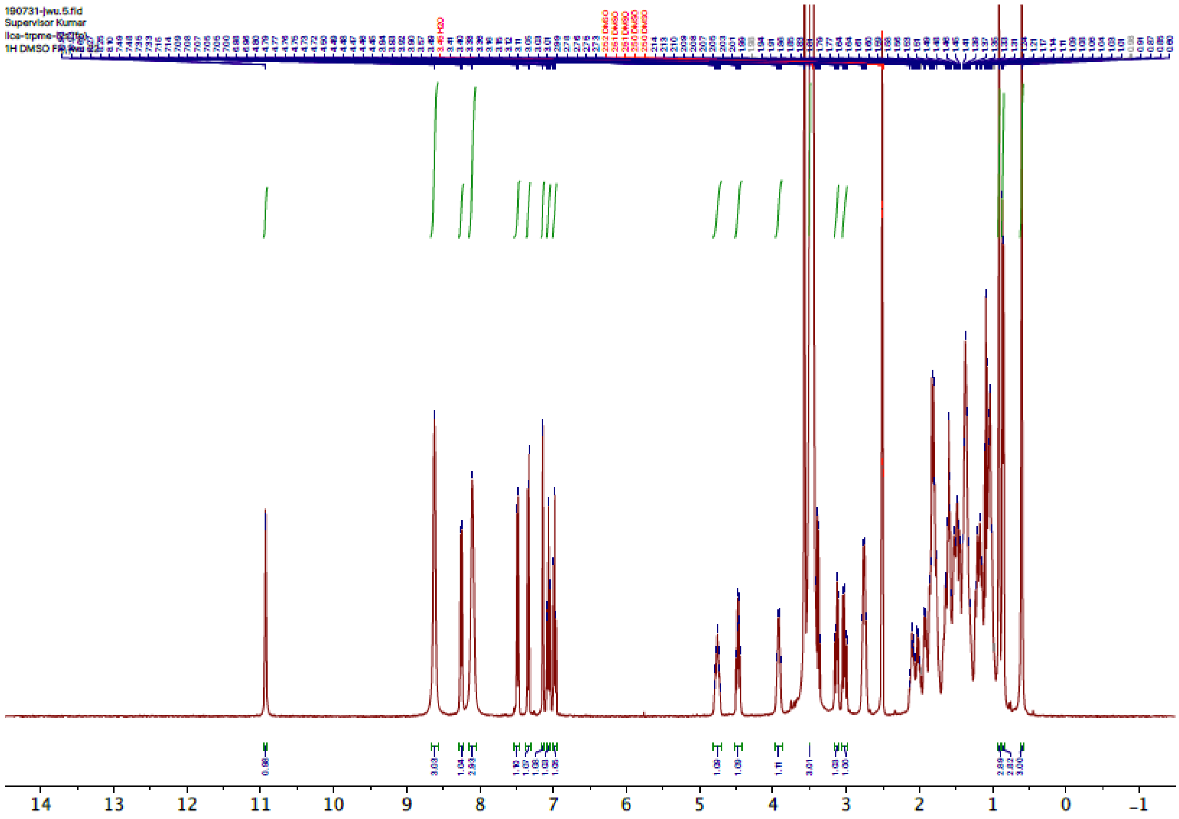

$^{13}$C NMR (75 MHz, DMSO-$d_6$):

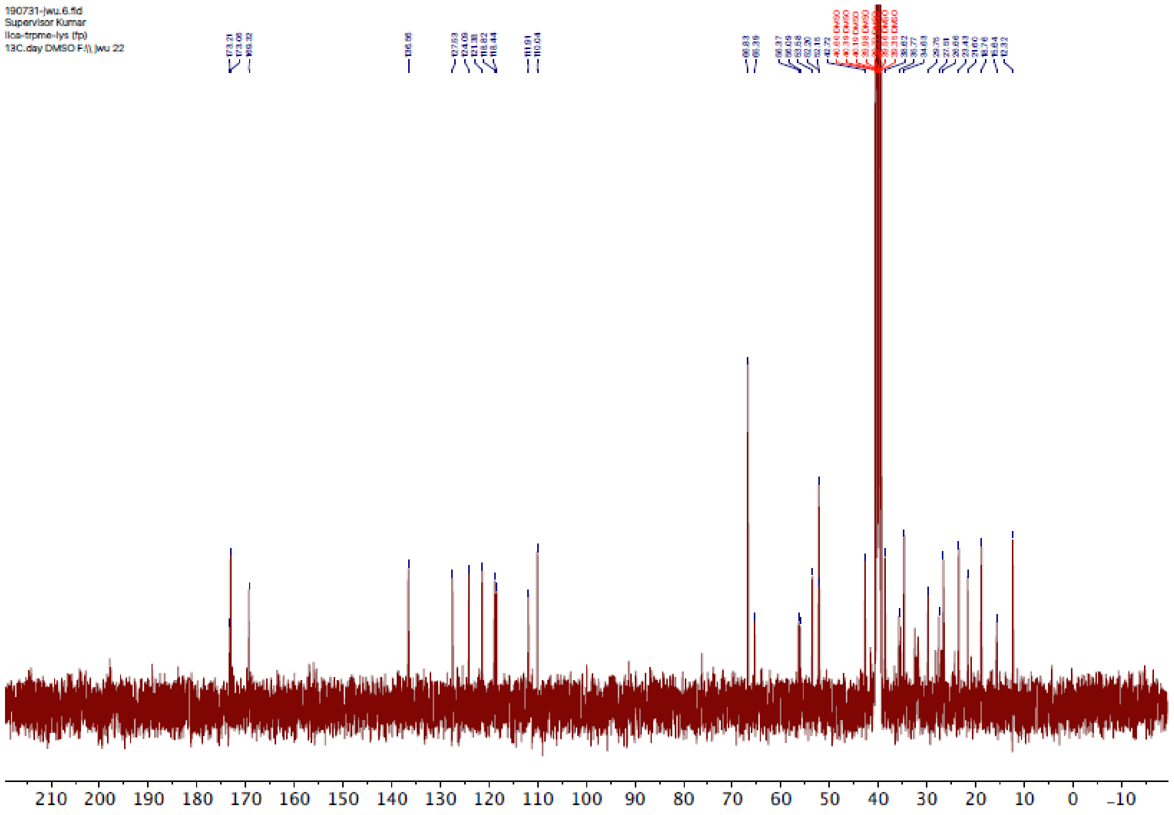

2,2'-(((3*R*,5*S*,7*R*,8*R*,9*S*,10*S*,13*R*,14*S*,17*R*)-17-((*R*)-5-(((*S*)-3-(1*H*-Indol-3-yl)-1-methoxy-1-oxopropan-2-yl)amino)-5-oxopentan-2-yl)-10,13-dimethylhexadecahydro-1*H*-cyclopenta[*a*]phenanthrene-3,7-diyl)bis(oxy))bis(2-oxoethan-1-aminium) **(17a)**

$^1$H NMR (400 MHz, DMSO-$d_6$):

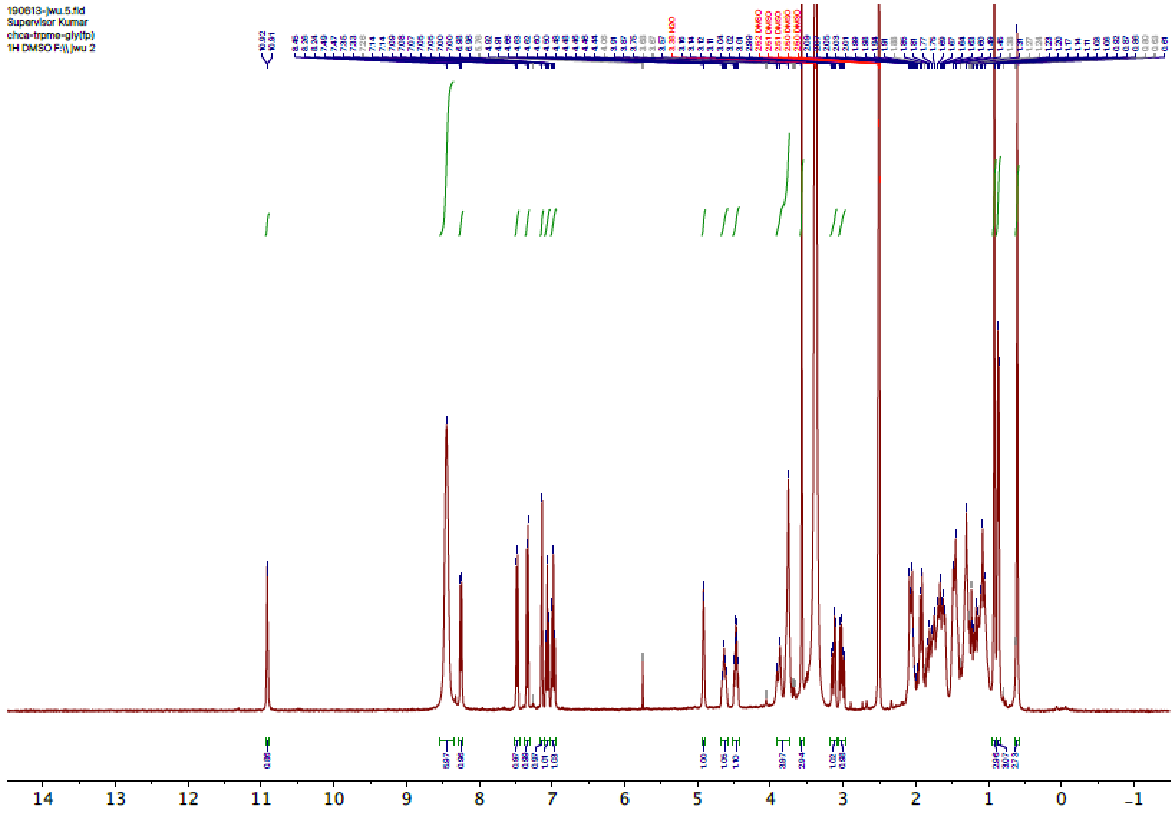

$^{13}$C NMR (100 MHz, DMSO-$d_6$):

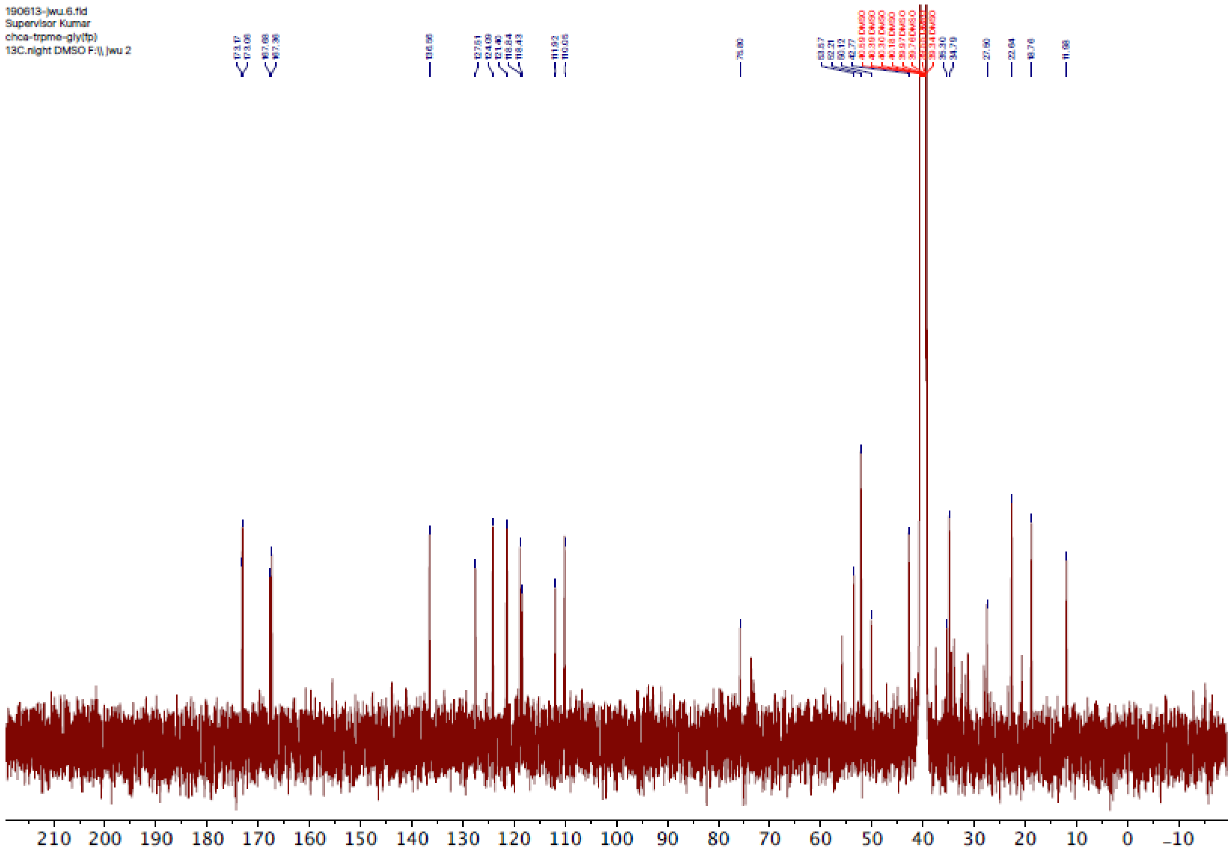

4,4'-(((3*R*,5*S*,7*R*,8*R*,9*S*,10*S*,13*R*,14*S*,17*R*)-17-((*R*)-5-(((*S*)-3-(1*H*-Indol-3-yl)-1-methoxy-1-oxopropan-2-yl)amino)-5-oxopentan-2-yl)-10,13-dimethylhexadecahydro-1*H*-cyclopenta[*a*]phenanthrene-3,7-diyl)bis(oxy))bis(4-oxobutan-1-aminium) **(17b)**

$^{1}$H NMR (400 MHz, DMSO-$d_6$):

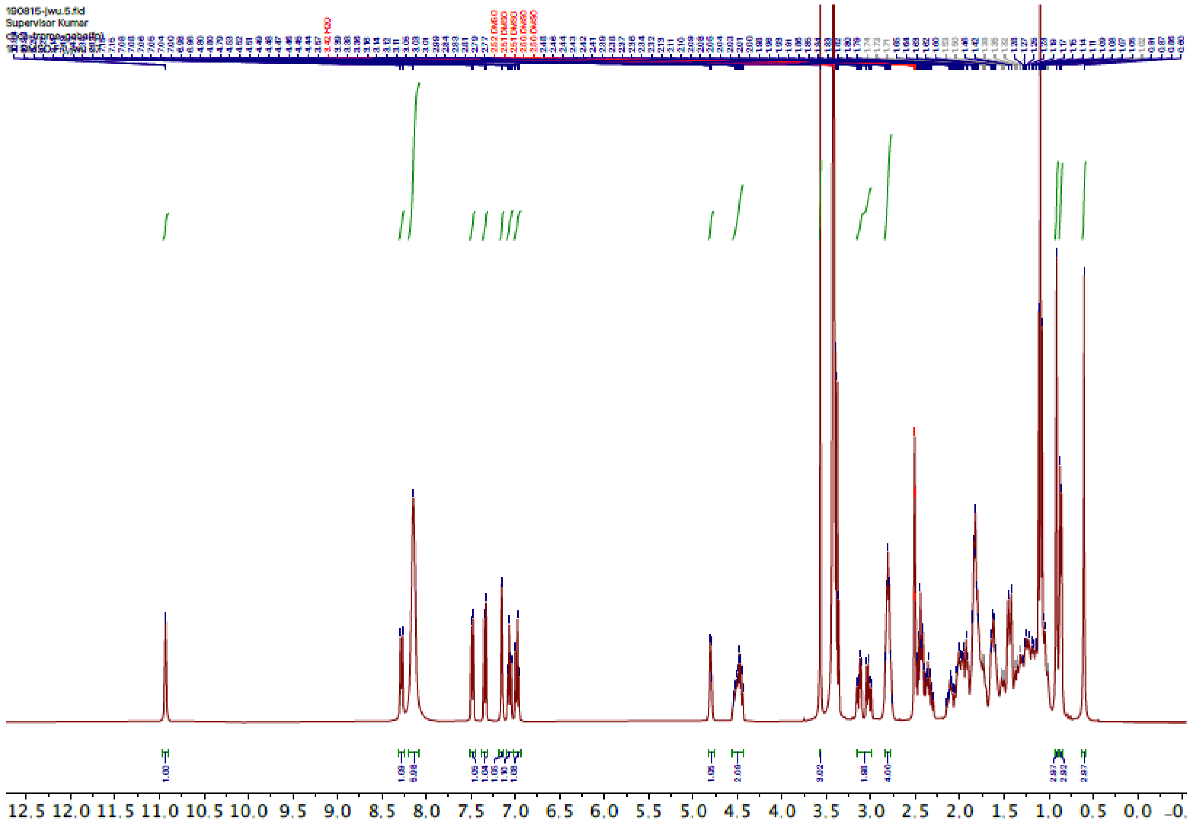

$^{13}$C NMR (100 MHz, DMSO-$d_6$):

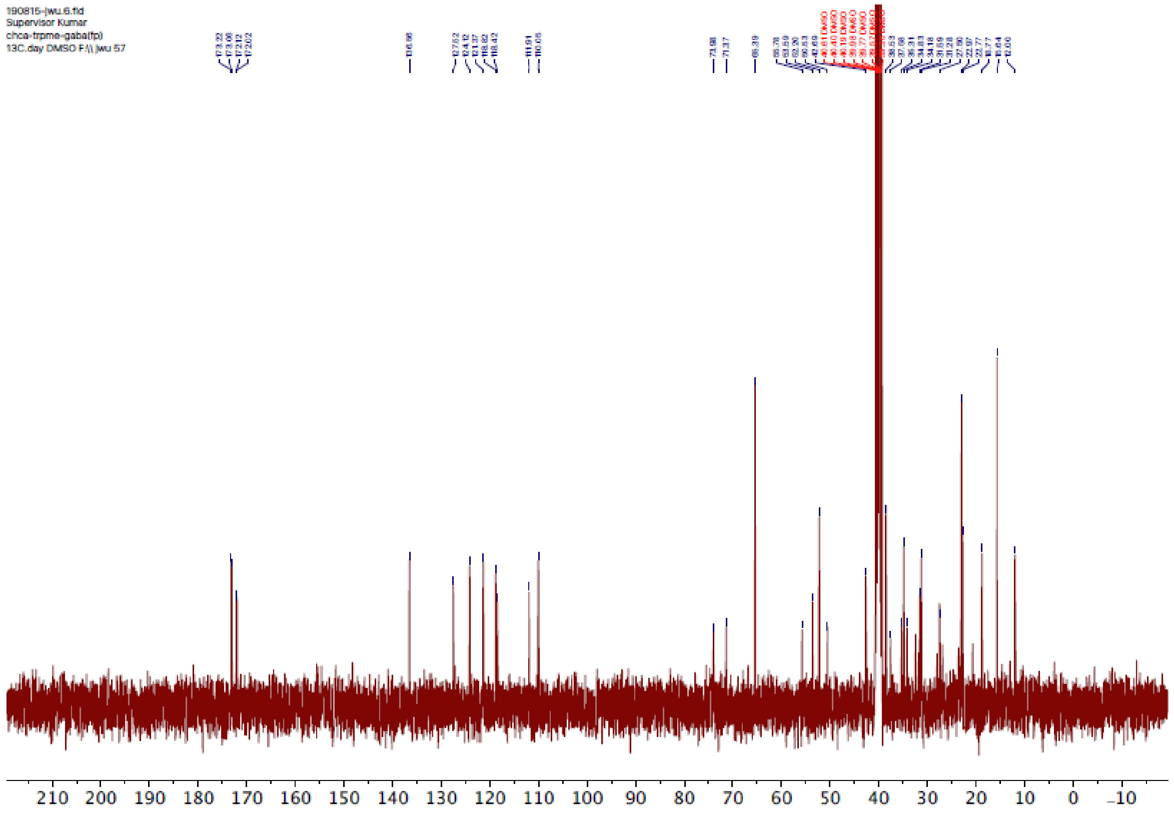

(4*S*,4'*S*)-5,5'-(((3*R*,5*S*,7*R*,8*R*,9*S*,10*S*,13*R*,14*S*,17*R*)-17-((*R*)-5-(((*S*)-3-(1*H*-Indol-3-yl)-1-methoxy-1-oxopropan-2-yl)amino)-5-oxopentan-2-yl)-10,13-dimethylhexadecahydro-1*H*-cyclopenta[*a*]phenanthrene-3,7-diyl)bis(oxy))bis(5-oxopentane-1,4-diaminium) **(17c)**

$^1$H NMR (400 MHz, DMSO-$d_6$):

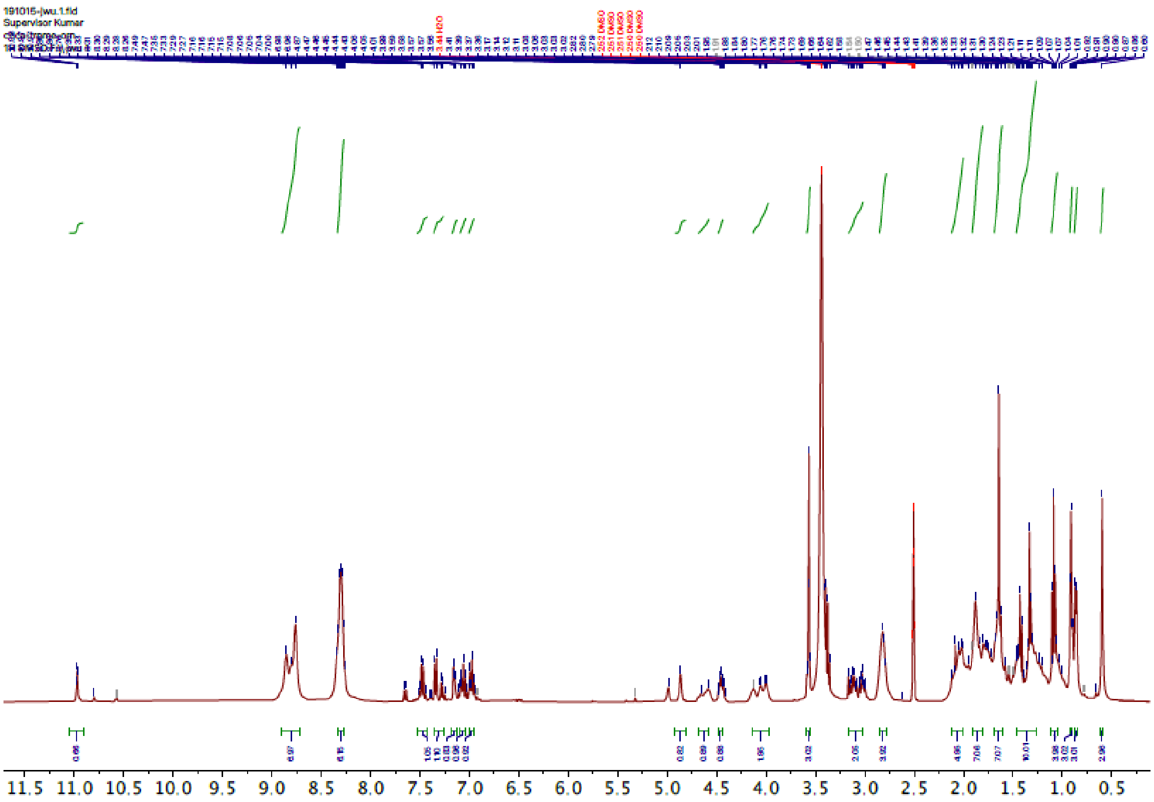


$^{13}$C NMR (100 MHz, DMSO-$d_6$):

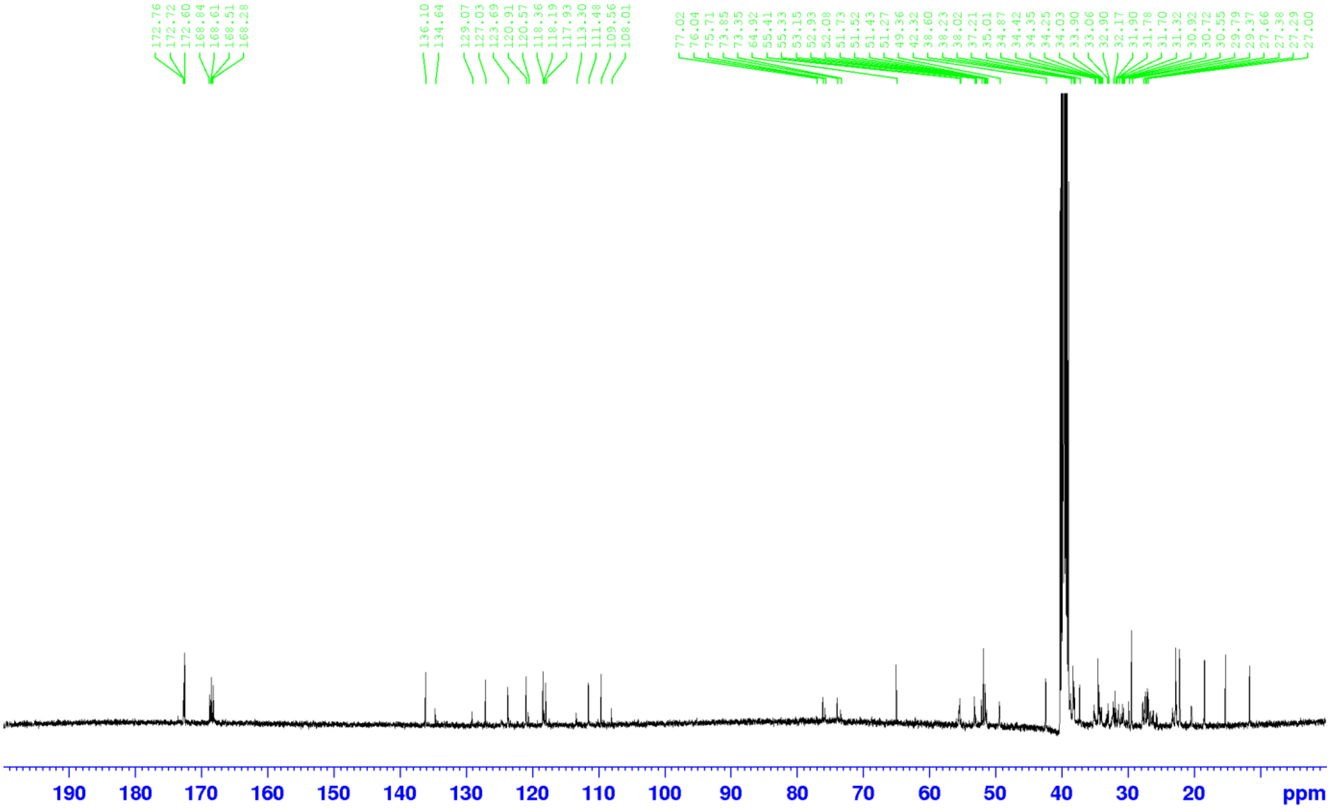

(5*S*,5'*S*)-6,6'-(((3*R*,5*S*,7*R*,8*R*,9*S*,10*S*,13*R*,14*S*,17*R*)-17-((*R*)-5-(((*S*)-3-(1*H*-Indol-3-yl)-1-methoxy-1-oxopropan-2-yl)amino)-5-oxopentan-2-yl)-10,13-dimethylhexadecahydro-1*H*-cyclopenta[*a*]phenanthrene-3,7-diyl)bis(oxy))bis(6-oxohexane-1,5-diaminium) **(17d)**

$^{1}$H NMR (600 MHz, DMSO-$d_6$):

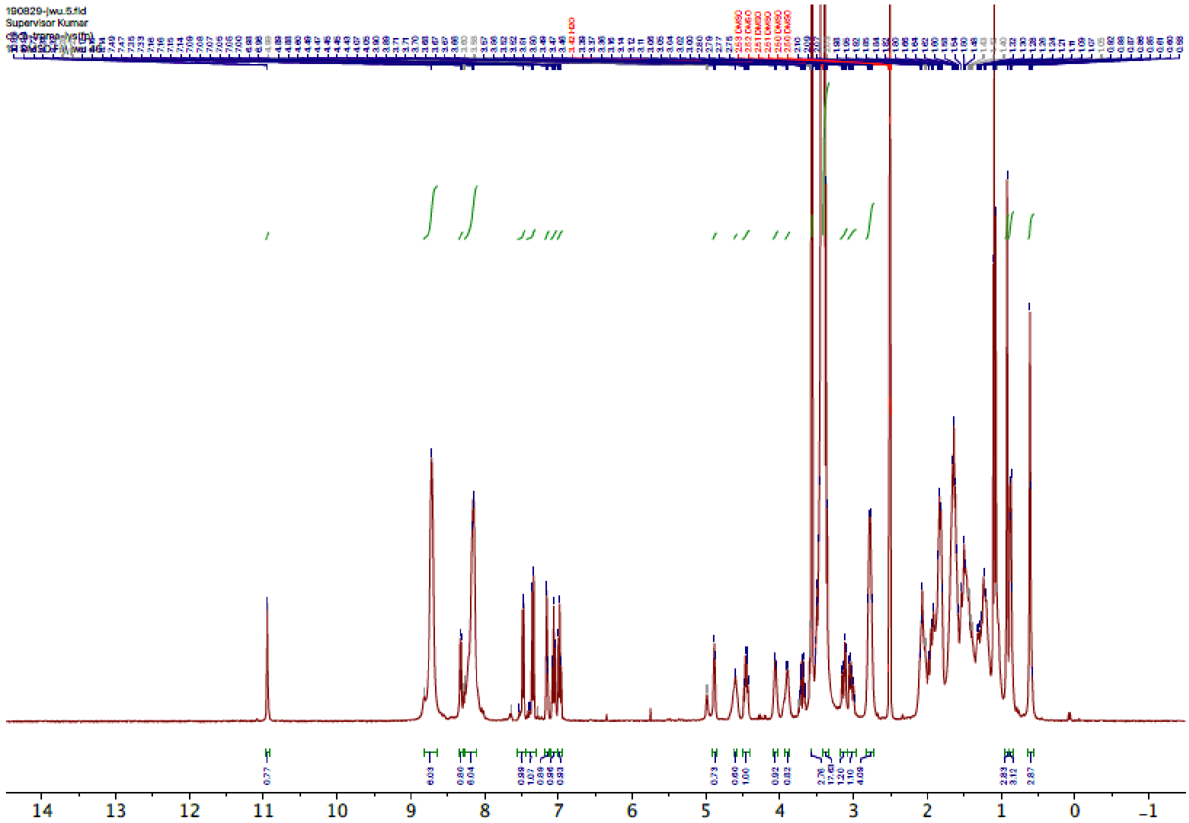

$^{13}$C NMR (150 MHz, DMSO-$d_6$):

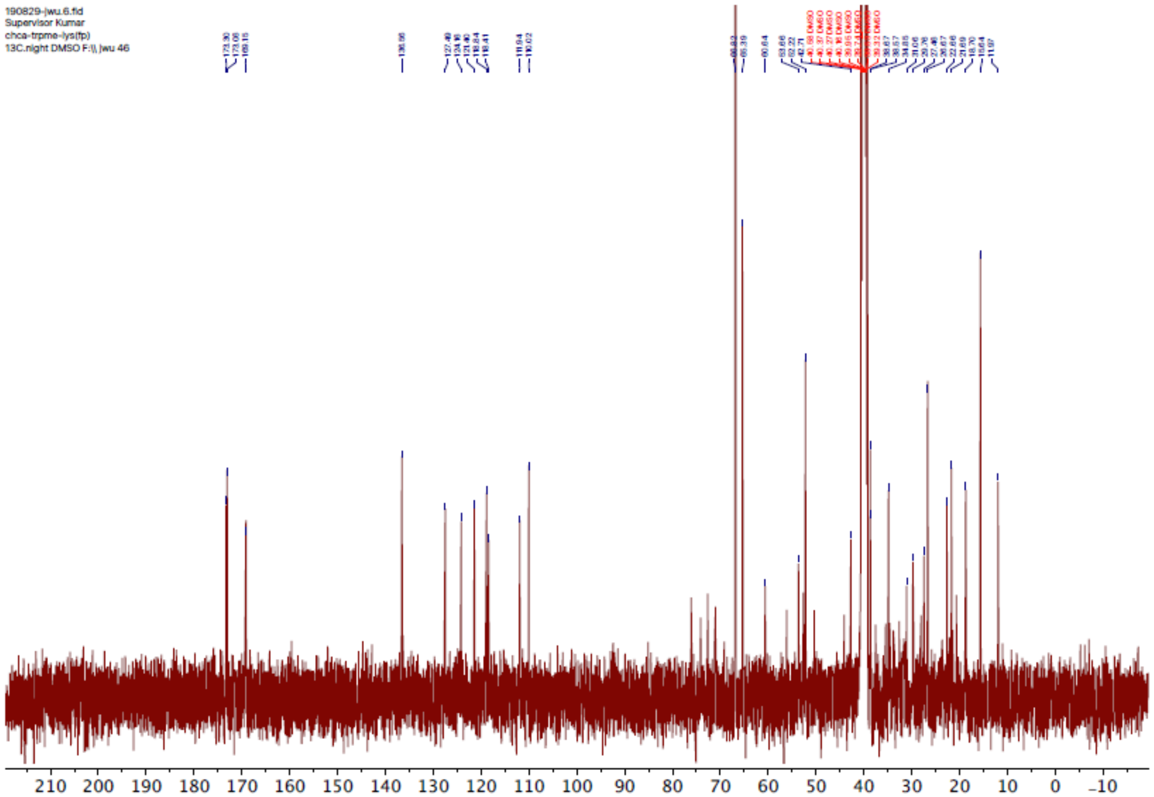

2,2'-(((3*R*,5*R*,8*R*,9*S*,10*S*,12*S*,13*R*,14*S*,17*R*)-17-((*R*)-5-(((*S*)-3-(1*H*-Indol-3-yl)-1-methoxy-1-oxopropan-2-yl)amino)-5-oxopentan-2-yl)-10,13-dimethylhexadecahydro-1*H*-cyclopenta[*a*]phenanthrene-3,12-diyl)bis(oxy))bis(2-oxoethan-1-aminium) **(18a)**

$^1$H NMR (300 MHz, DMSO-$d_6$):

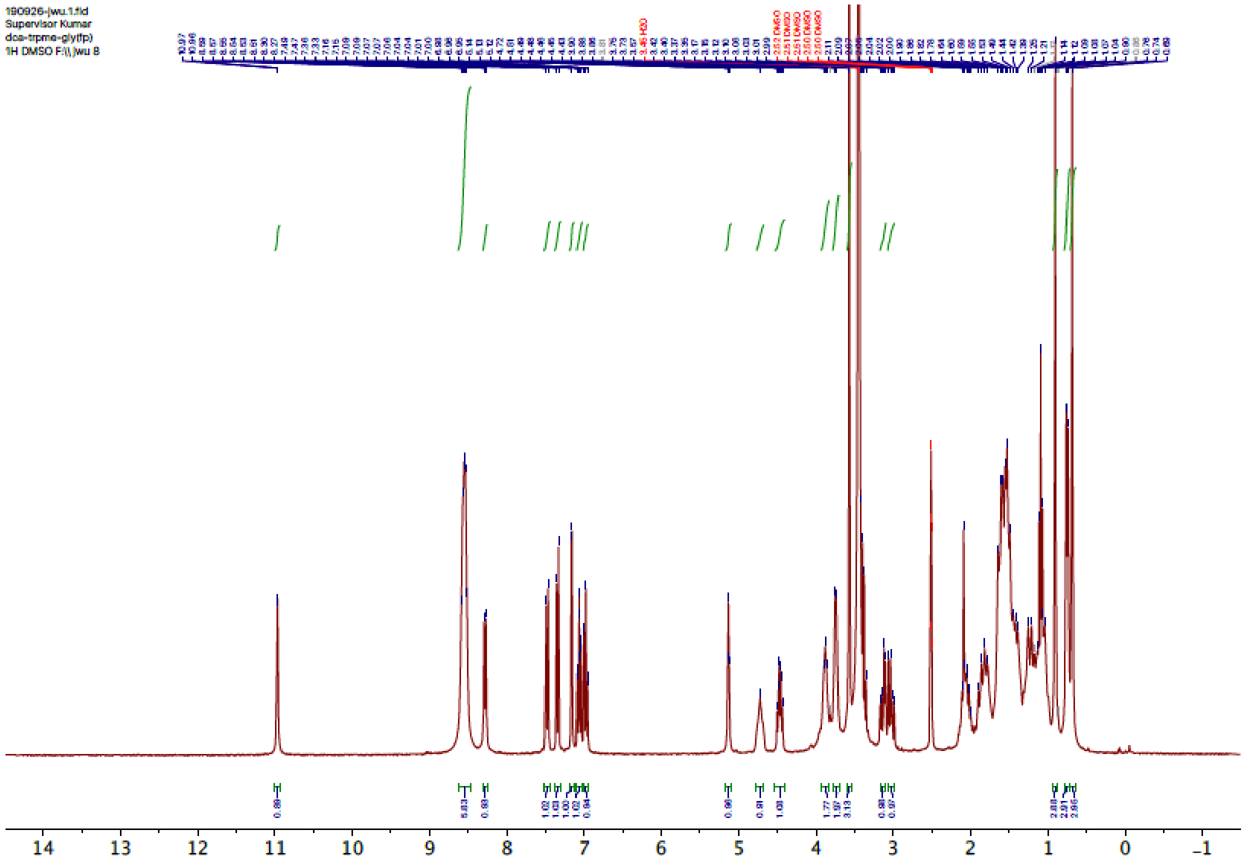

$^{13}$C NMR (75 MHz, DMSO-$d_6$):

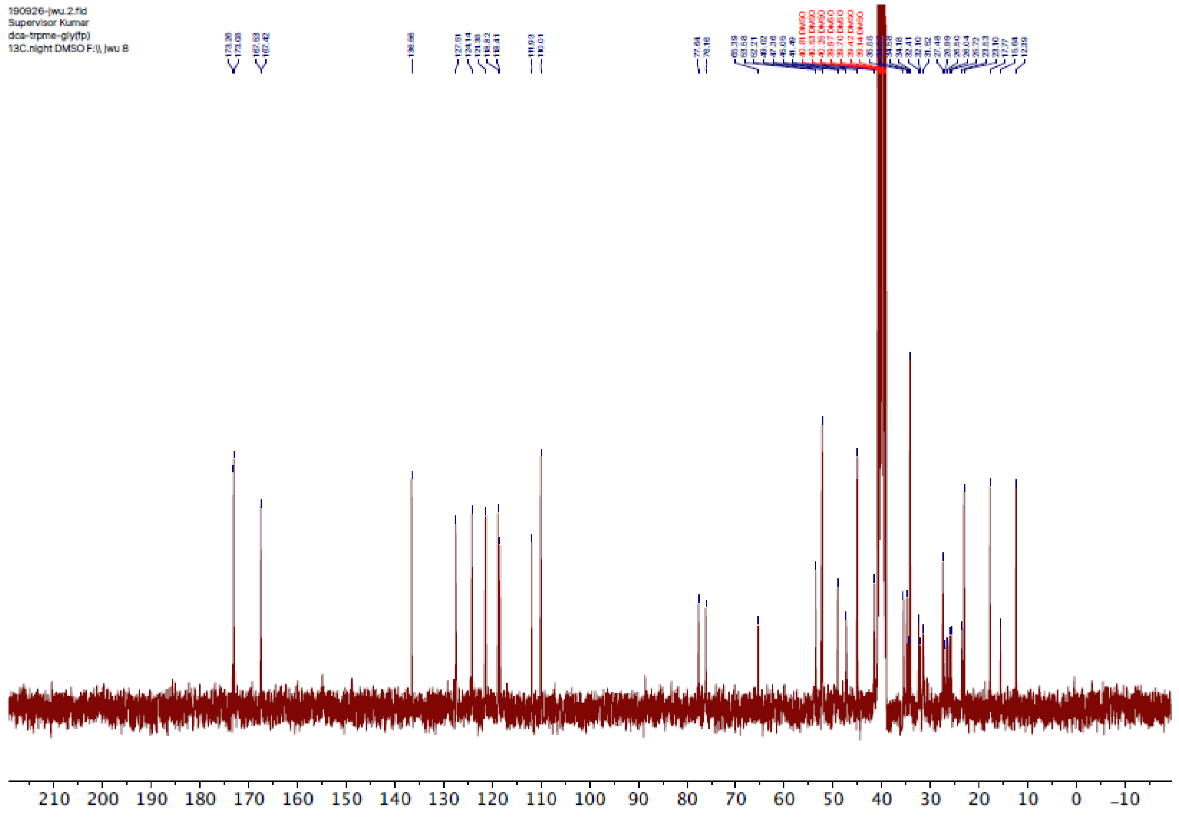

2,2'-(((3*R*,5*S*,7*R*,8*R*,9*S*,10*S*,12*S*,13*R*,14*S*,17*R*)-17-((*R*)-5-(((*S*)-3-(1*H*-Indol-3-yl)-1-methoxy-1-oxopropan-2-yl)amino)-5-oxopentan-2-yl)-3-(2-aminoacetoxy)-10,13-dimethylhexadecahydro-1*H*-cyclopenta[*a*]phenanthrene-7,12-diyl)bis(oxy))bis(2-oxoethan-1-aminium) **(19a)**

$^{1}$H NMR (400 MHz, DMSO-$d_6$):

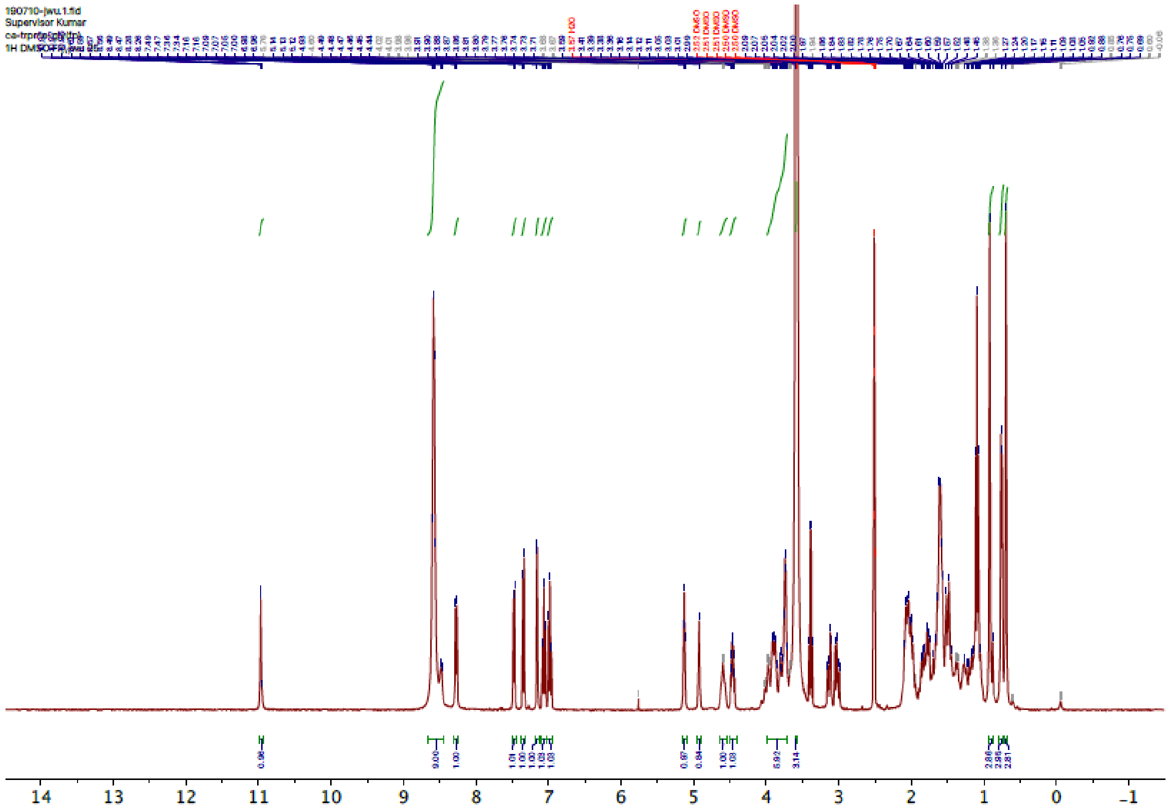

$^{13}$C NMR (100 MHz, DMSO-$d_6$):

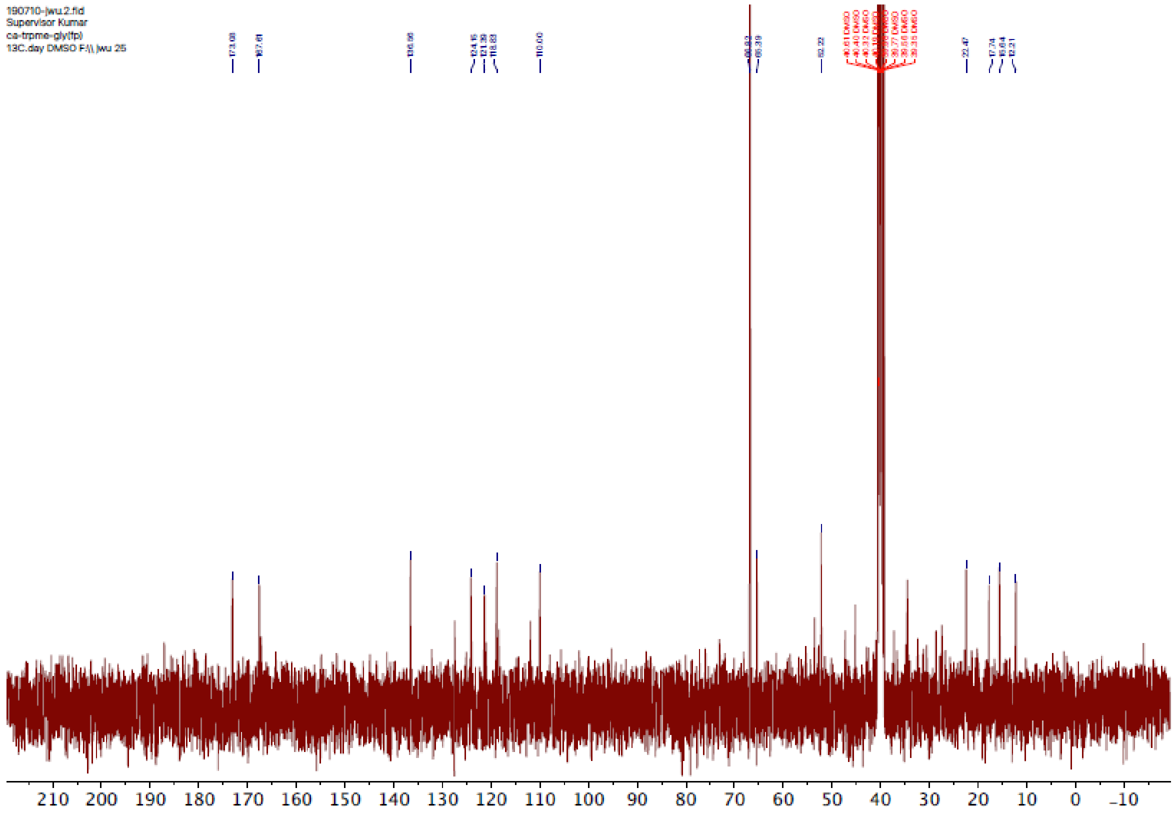

4,4',4''-(((3*R*,5*S*,7*R*,8*R*,9*S*,10*S*,12*S*,13*R*,14*S*,17*R*)-17-((*R*)-5-(((*S*)-3-(1*H*-Indol-3-yl)-1-methoxy-1-oxopropan-2-yl)amino)-5-oxopentan-2-yl)-10,13-dimethylhexadecahydro-1*H*-cyclopenta[*a*]phenanthrene-3,7,12-triyl)tris(oxy))tris(4-oxobutan-1-aminium) **(19b)**

$^{1}$H NMR (400 MHz, DMSO-$d_6$):

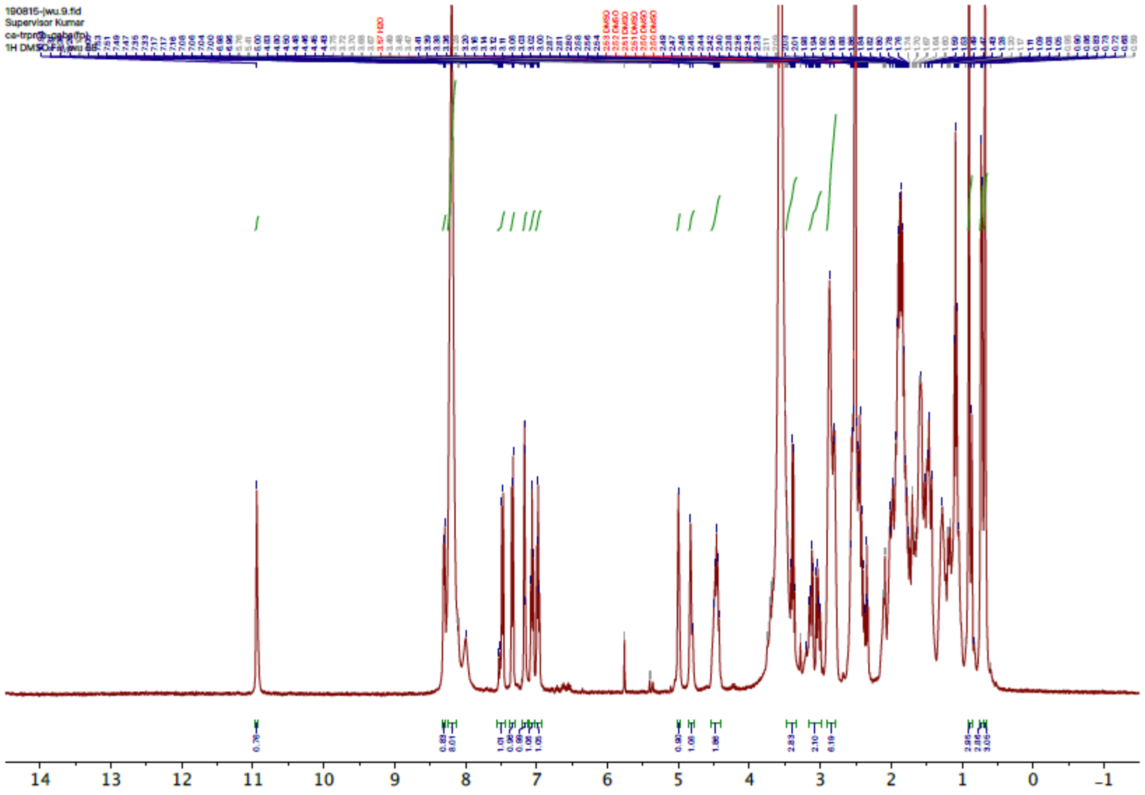


$^{13}$C NMR (100 MHz, DMSO-$d_6$):

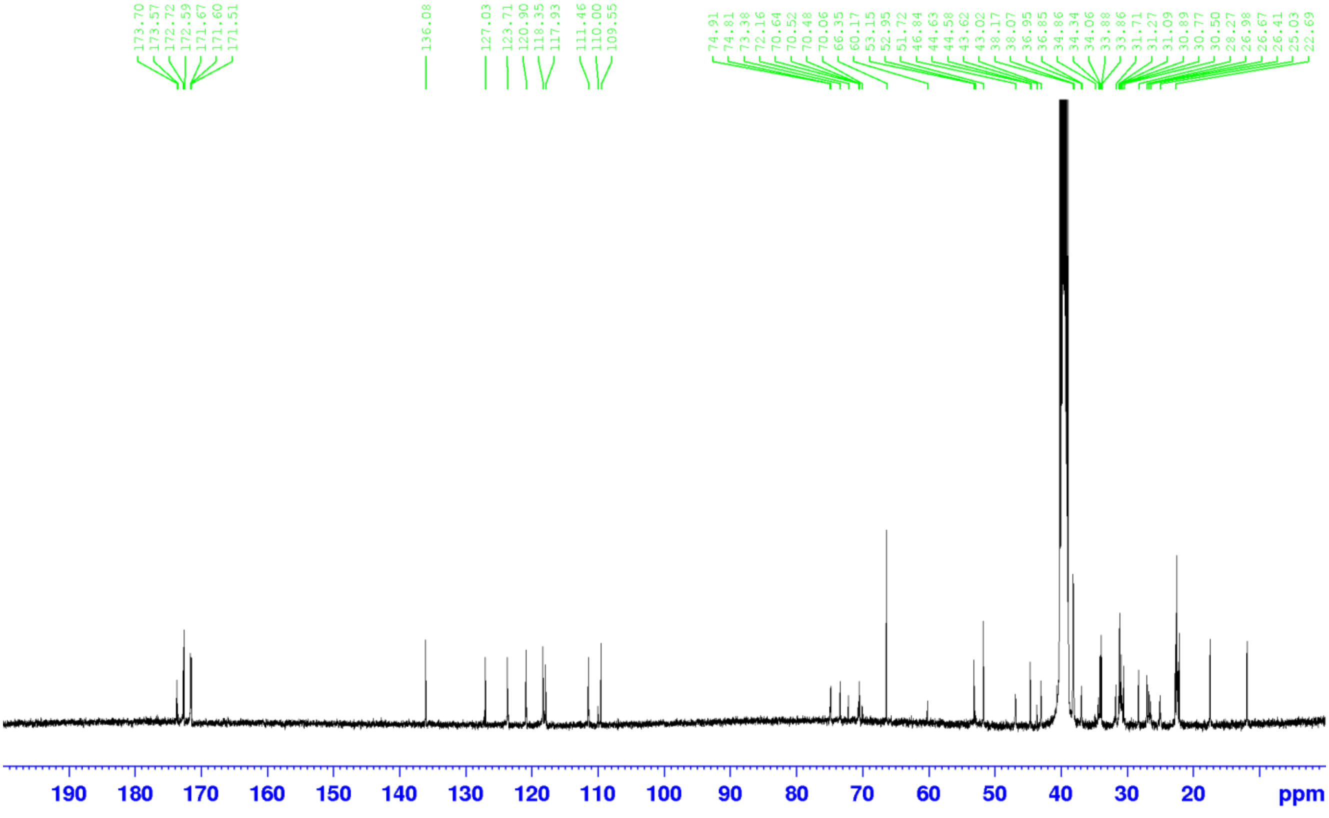

(4*S*,4'*S*,4''*S*)-5,5',5''-(((3*R*,5*S*,7*R*,8*R*,9*S*,10*S*,12*S*,13*R*,14*S*,17*R*)-17-((*R*)-5-(((*S*)-3-(1*H*-Indol-3-yl)-1-methoxy-1-oxopropan-2-yl)amino)-5-oxopentan-2-yl)-10,13-dimethylhexadecahydro-1*H*-cyclopenta[*a*]phenanthrene-3,7,12-triyl)tris(oxy))tris(5-oxopentane-1,4-diaminium) **(19c)**

$^{1}$H NMR (600 MHz, DMSO-$d_6$):

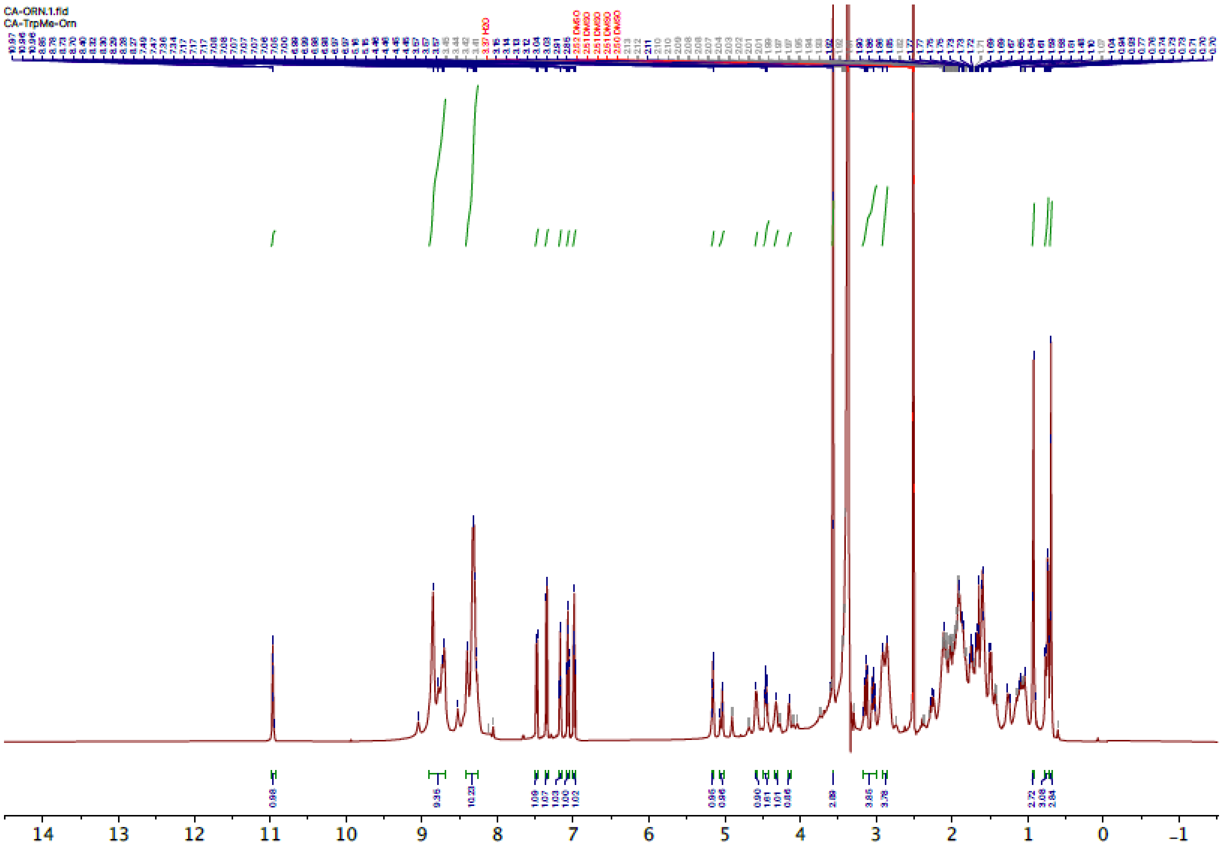

$^{13}$C NMR (150 MHz, DMSO-$d_6$):

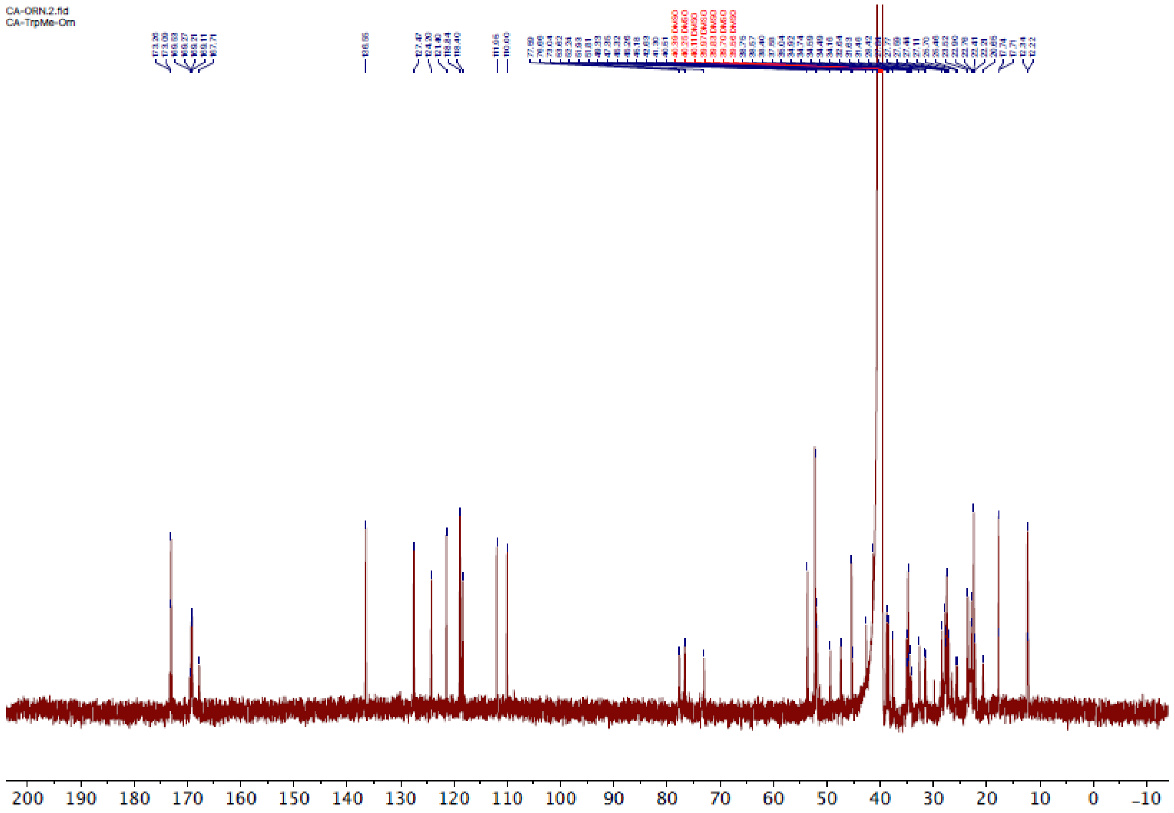

(5*S*,5'*S*,5"*S*)-6,6',6"-(((3*R*,5*S*,7*R*,8*R*,9*S*,10*S*,12*S*,13*R*,14*S*,17*R*)-17-((*R*)-5-(((*S*)-3-(1*H*-Indol-3-yl)-1-methoxy-1-oxopropan-2-yl)amino)-5-oxopentan-2-yl)-10,13-dimethylhexadecahydro-1*H*-cyclopenta[*a*]phenanthrene-3,7,12-triyl)tris(oxy))tris(6-oxohexane-1,5-diaminium) **(19d)**

$^{1}$H NMR (600 MHz, DMSO-$d_6$):

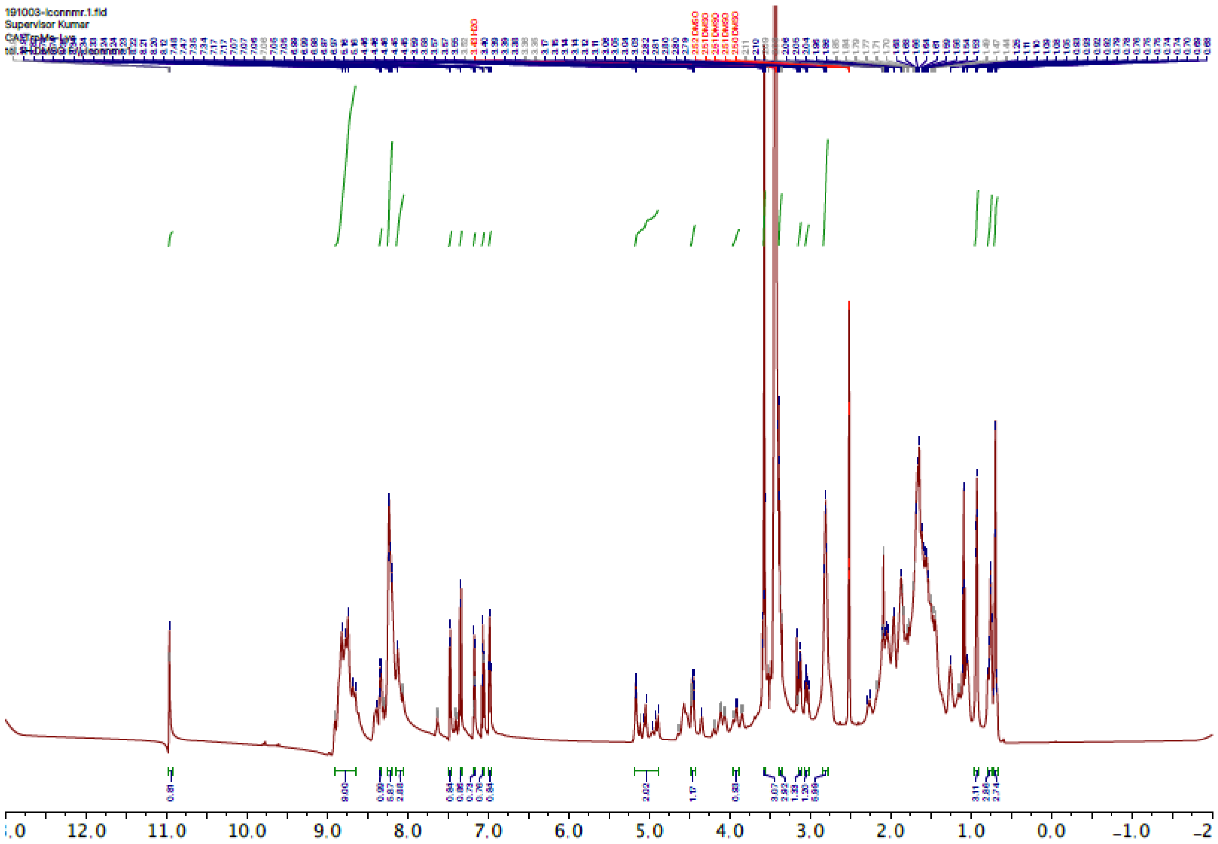

$^{13}$C NMR (150 MHz, DMSO-$d_6$):

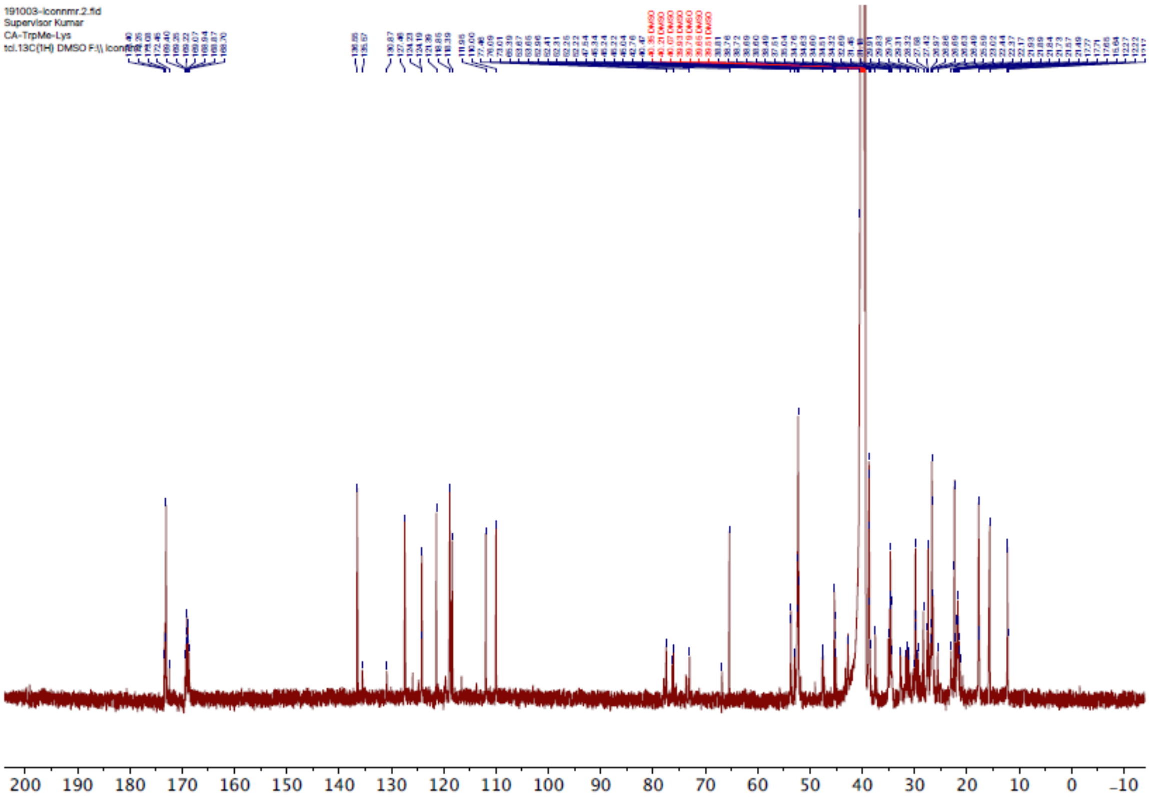

(4*S*,4'*S*)-5,5'-(((3*R*,5*S*,7*R*,8*R*,9*S*,10*S*,13*R*,14*S*,17*R*)-17-((*R*)-5-(((*S*)-1-Methoxy-1-oxo-3-phenylpropan-2-yl)amino)-5-oxopentan-2-yl)-10,13-dimethylhexadecahydro-1*H*-cyclopenta[*a*]phenanthrene-3,7-diyl)bis(oxy))bis(5-oxopentane-1,4-diaminium) **(20)**

$^{1}$H NMR (400 MHz, DMSO-$d_6$):

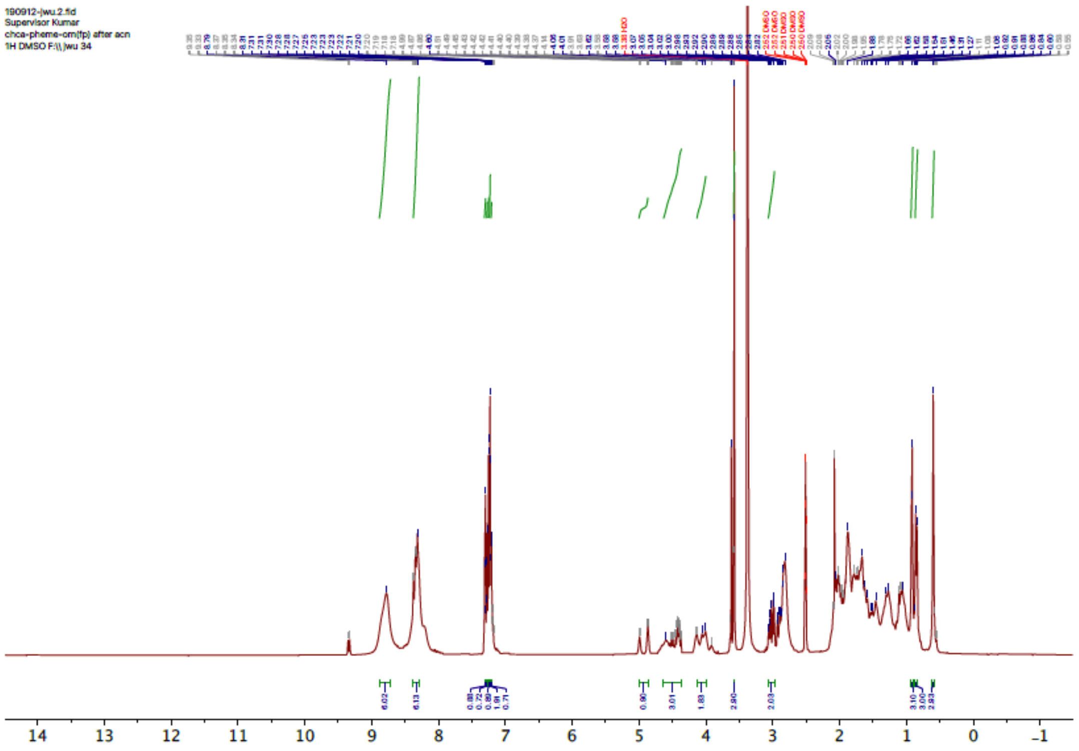


(4*S*,4'*S*)-5,5'-(((3*R*,5*S*,7*R*,8*R*,9*S*,10*S*,13*R*,14*S*,17*R*)-17-((*R*)-5-(Benzylamino)-5-oxopentan-2-yl)-10,13-dimethylhexadecahydro-1*H*-cyclopenta[*a*]phenanthrene-3,7-diyl)bis(oxy))bis(5-oxopentane-1,4-diaminium) **(21)**

$^{1}$H NMR (400 MHz, DMSO-$d_6$):

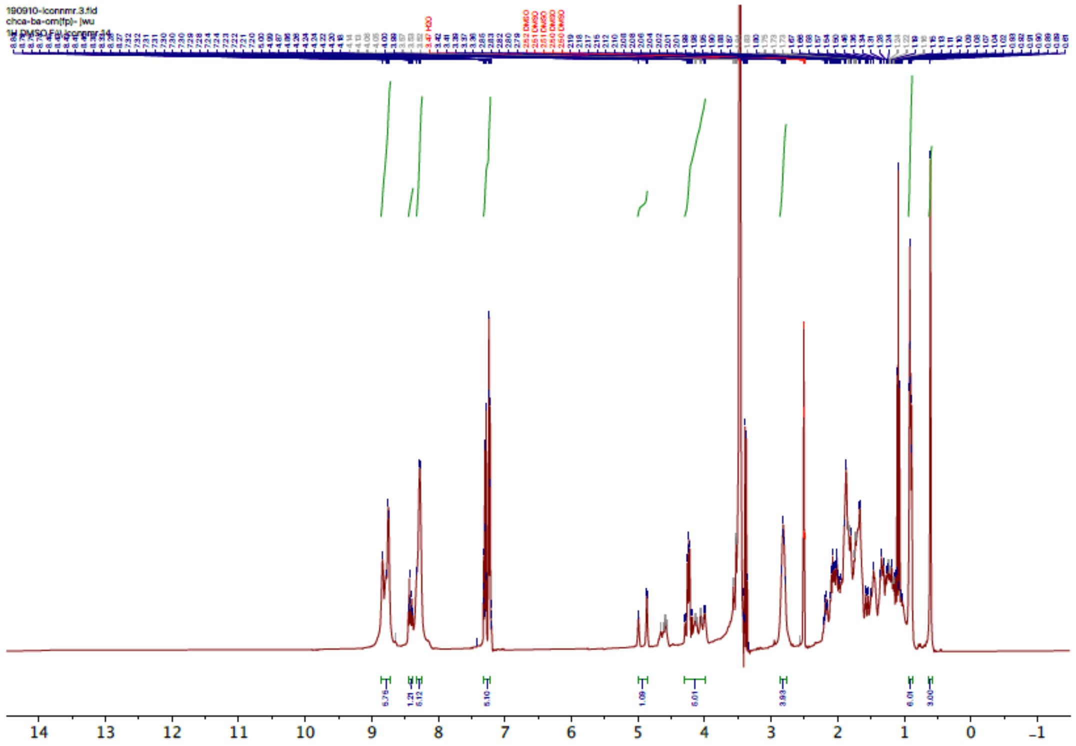

((4-(((3*R*,5*R*,8*R*,9*S*,10*S*,13*R*,14*S*,17*R*)-17-((*R*)-5-(((*S*)-3-(1*H*-Indol-3-yl)-1-methoxy-1-oxopropan-2-yl)amino)-5-oxopentan-2-yl)-10,13-dimethylhexadecahydro-1*H*-cyclopenta[*a*]phenanthren-3-yl)oxy)-4-oxobutyl)amino)(amino)methaniminium **(23)**

$^{1}$H NMR (400 MHz, DMSO-$d_6$):

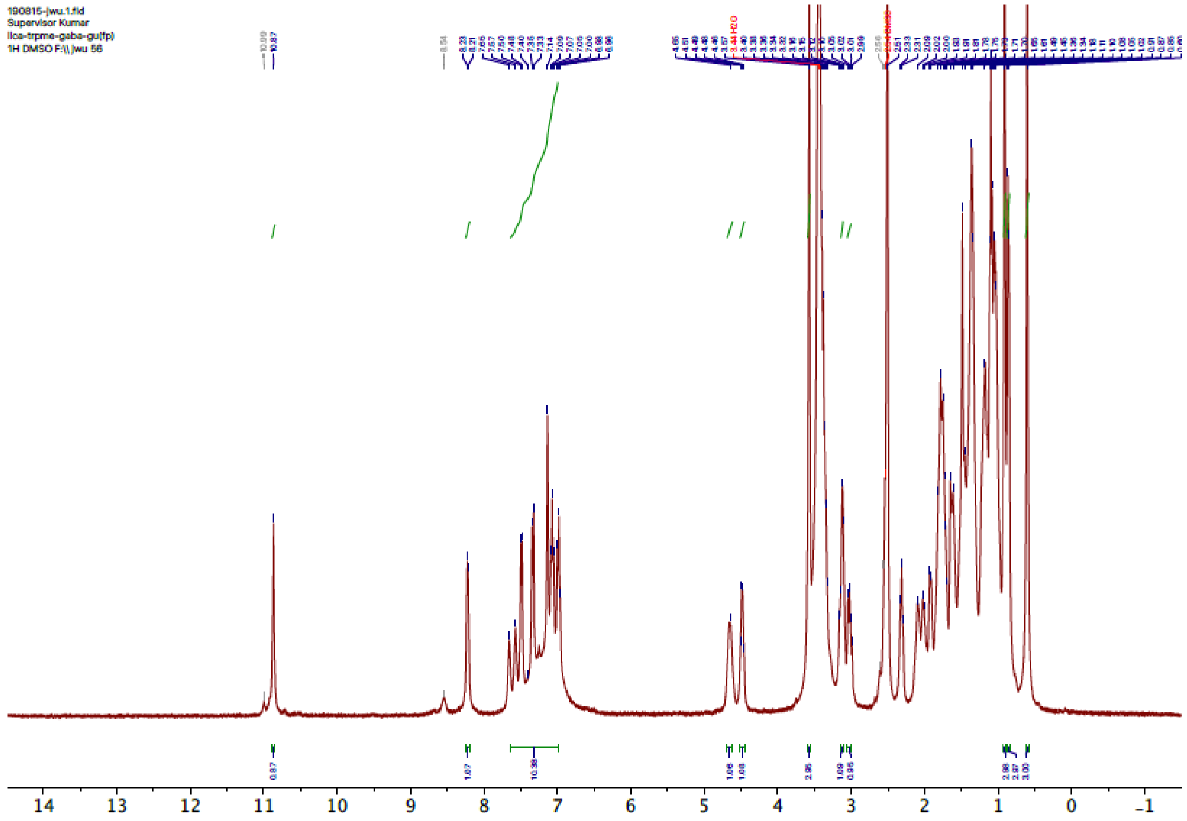


$^{13}$C NMR (75 MHz, DMSO-$d_6$):

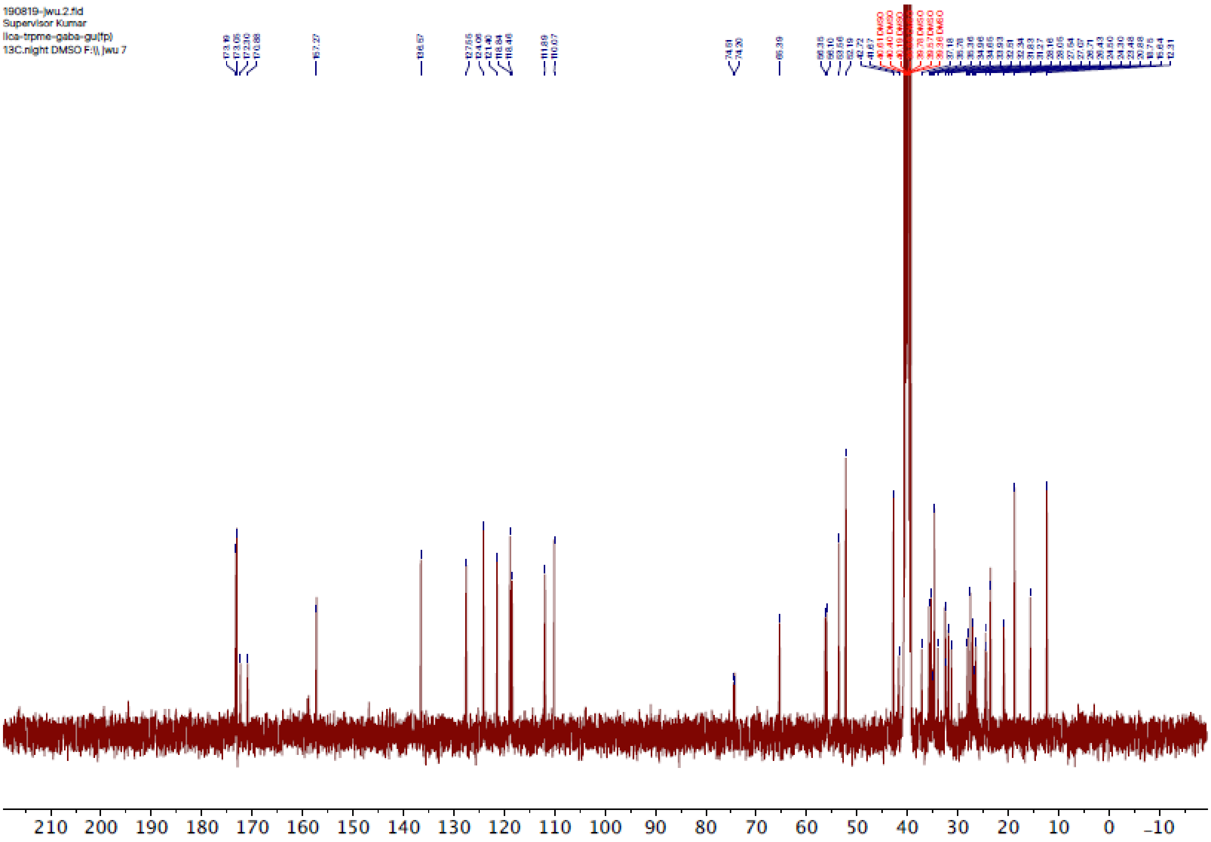

Methyl ((*R*)-4-((3*R*,5*R*,8*R*,9*S*,10*S*,13*R*,14*S*,17*R*)-3-((L-lysylglycyl)oxy)-10,13-dimethylhexadecahydro-1*H*-cyclopenta[*a*]phenanthren-17-yl)pentanoyl)-L-tryptophanate **(25)**

$^{1}$H NMR (400 MHz, DMSO-$d_6$):

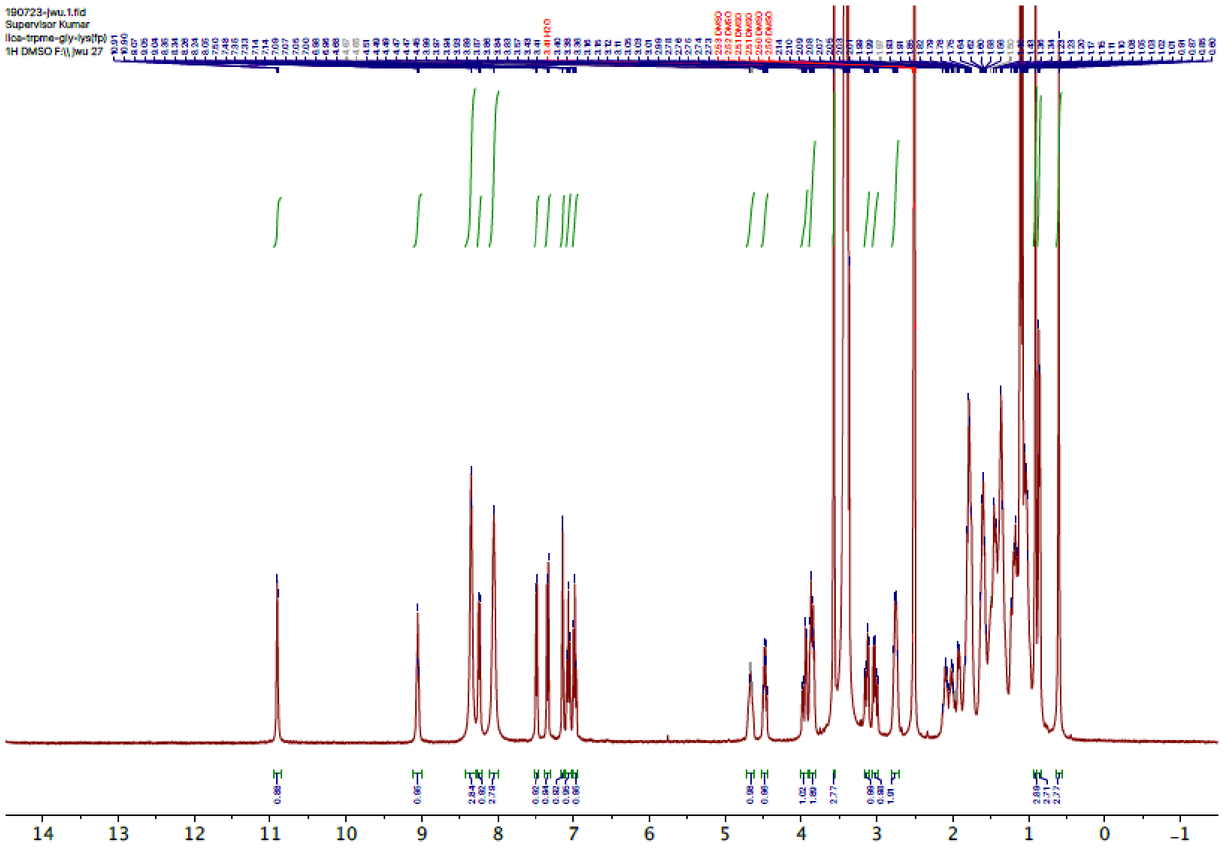

$^{13}$C NMR (75 MHz, DMSO-$d_6$):

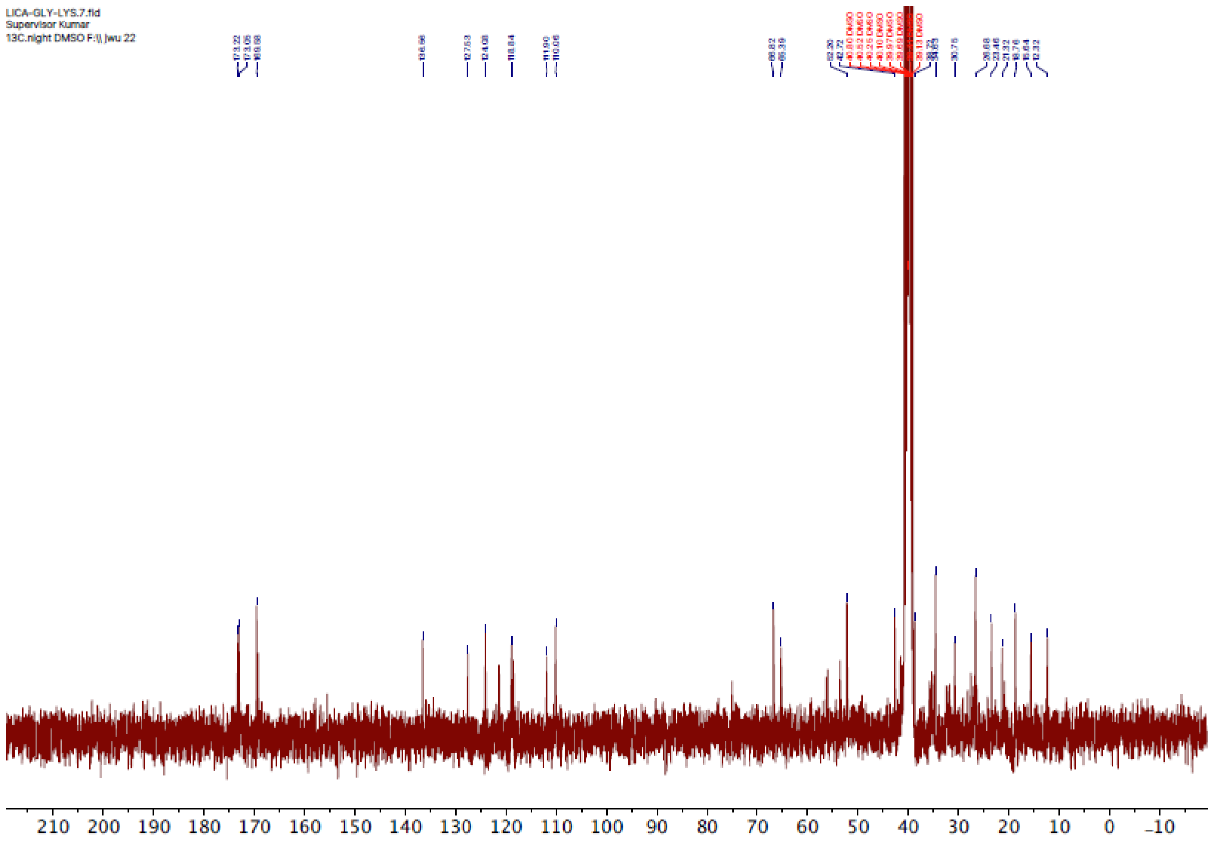

1,1',1''-(((((3*R*,5*S*,7*R*,8*R*,9*S*,10*S*,12*S*,13*R*,14*S*,17*R*)-17-((*R*)-5-(((*S*)-3-(1*H*-Indol-3-yl)-1-methoxy-1-oxopropan-2-yl)amino)-5-oxopentan-2-yl)-10,13-dimethylhexadecahydro-1*H*-cyclopenta[*a*]phenanthrene-3,7,12-triyl)tris(oxy))tris(2-oxoethane-2,1-diyl))tris(azanediyl))tris(3-methyl-1-oxobutan-2-aminium) **(27)**

$^{1}$H NMR (600 MHz, DMSO-$d_6$):

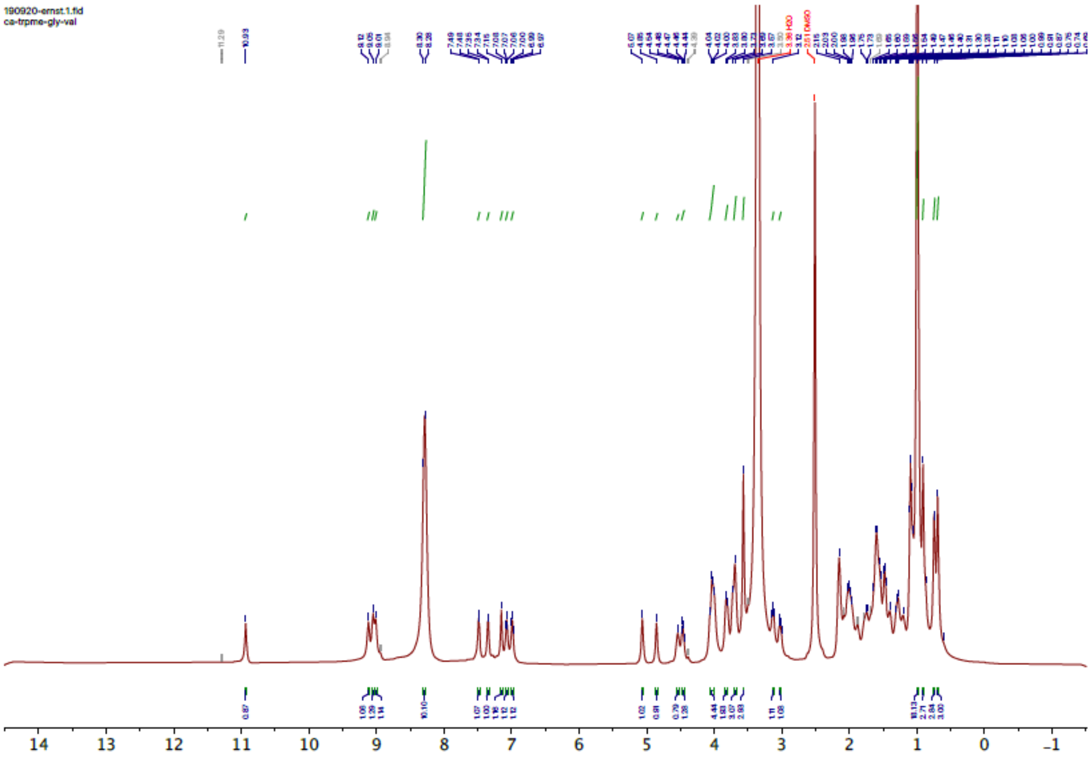

# High resolution mass spectra (HRMS) of synthesised compounds

2-(((3*R*,5*R*,8*R*,9*S*,10*S*,13*R*,14*S*,17*R*)-17-((*R*)-5-(((*S*)-3-(1*H*-Indol-3-yl)-1-methoxy-1-oxopropan-2-yl)amino)-5-oxopentan-2-yl)-10,13-dimethylhexadecahydro-1*H*-cyclopenta[*a*]phenanthren-3-yl)oxy)-2-oxoethan-1-aminium **(16a)**

Full spectrum

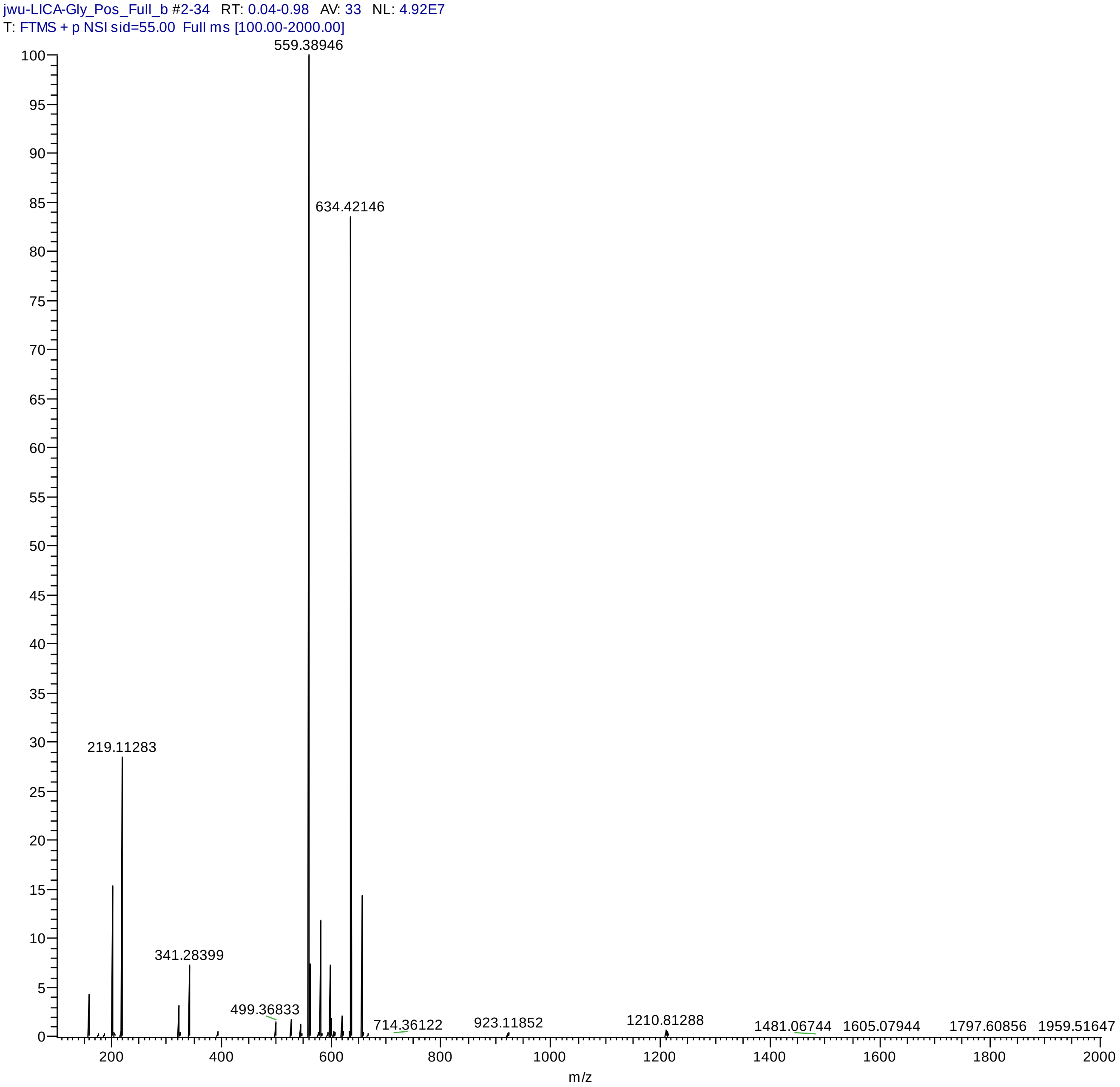

Zoomed spectrum (Top spectrum: measured mass; bottom spectrum: calculated mass)

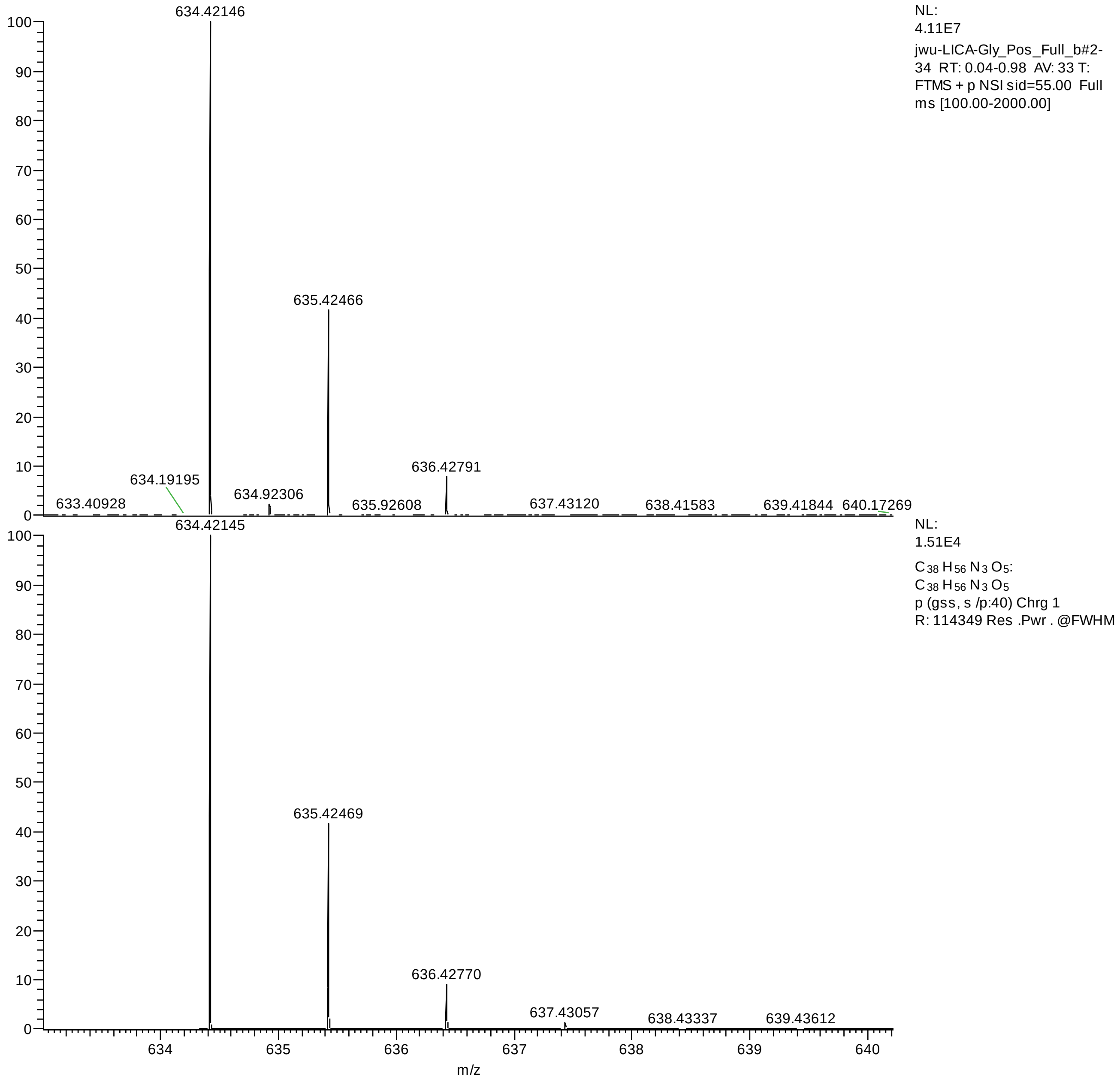

4-(((3*R*,5*R*,8*R*,9*S*,10*S*,13*R*,14*S*,17*R*)-17-((*R*)-5-(((*S*)-3-(1*H*-Indol-3-yl)-1-methoxy-1-oxopropan-2-yl)amino)-5-oxopentan-2-yl)-10,13-dimethylhexadecahydro-1*H*-cyclopenta[*a*]phenanthren-3-yl)oxy)-4-oxobutan-1-aminium **(16b)**

Full spectrum

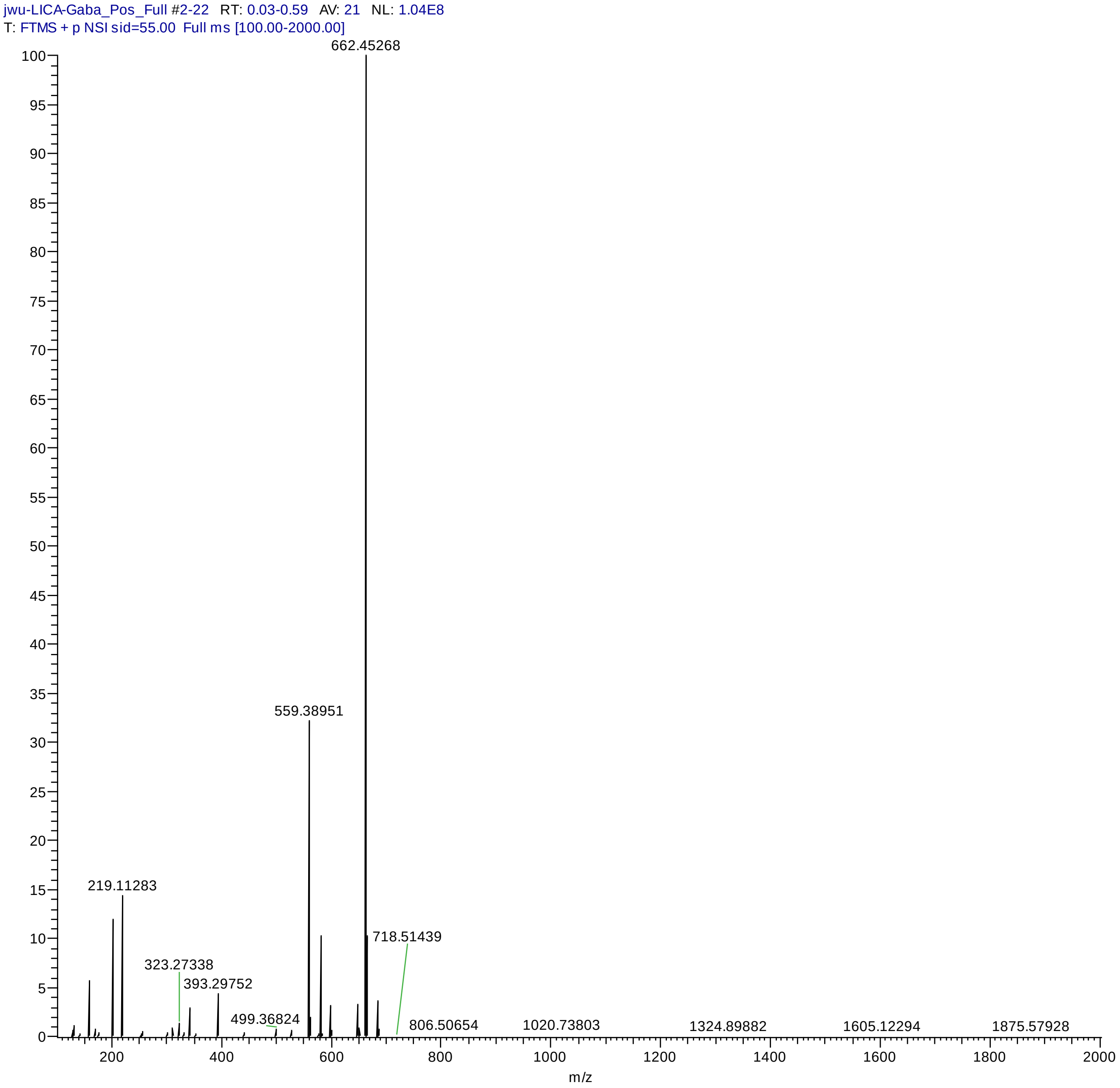

Zoomed spectrum (Top spectrum: measured mass; bottom spectrum: calculated mass)

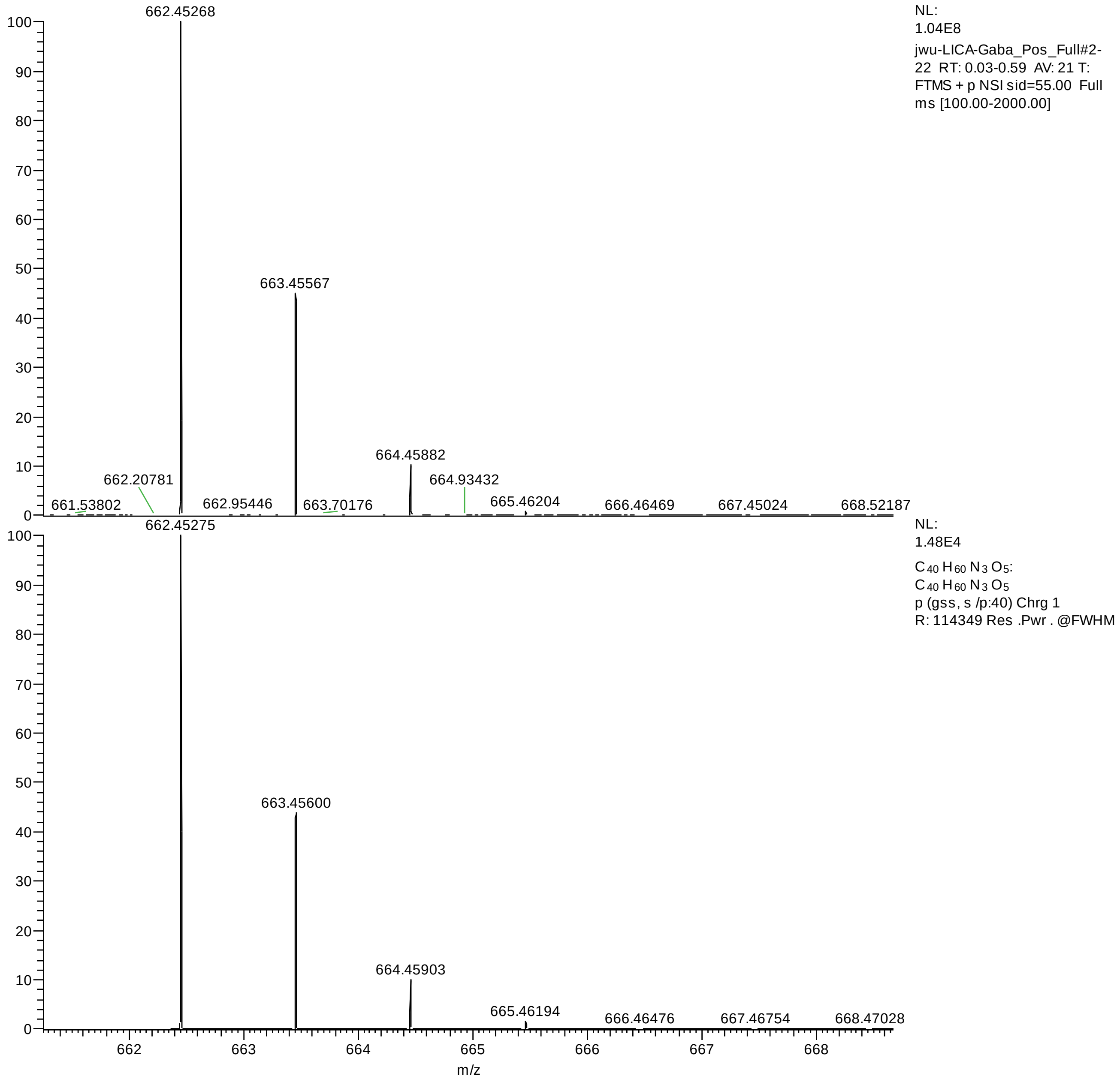

(*S*)-5-(((3*R*,5*R*,8*R*,9*S*,10*S*,13*R*,14*S*,17*R*)-17-((*R*)-5-(((*S*)-3-(1*H*-Indol-3-yl)-1-methoxy-1-oxopropan-2-yl)amino)-5-oxopentan-2-yl)-10,13-dimethylhexadecahydro-1*H*-cyclopenta[*a*]phenanthren-3-yl)oxy)-5-oxopentane-1,4-diaminium **(16c)**

Full spectrum

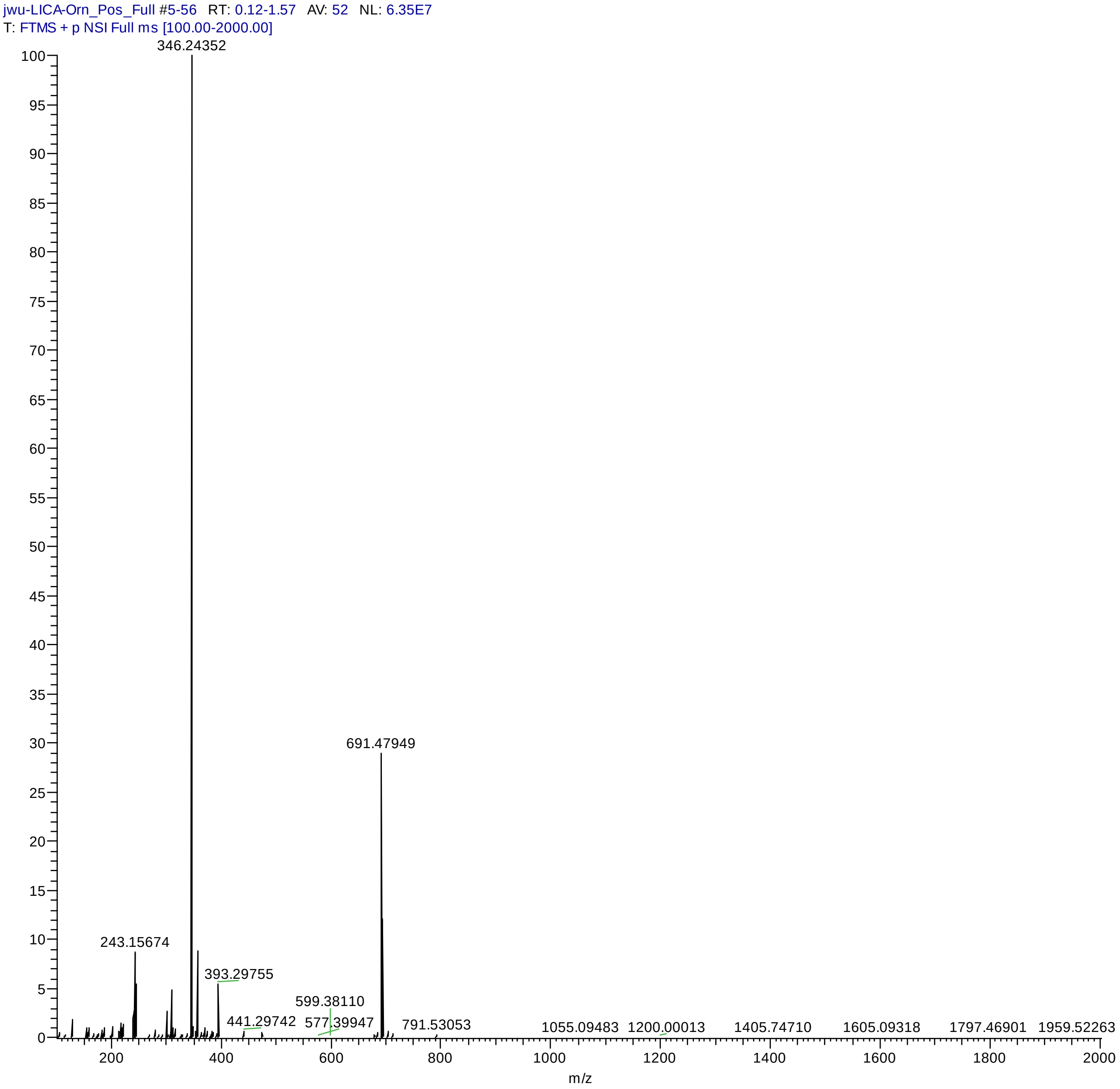

Zoomed spectrum (Top spectrum: measured mass; bottom spectrum: calculated mass)

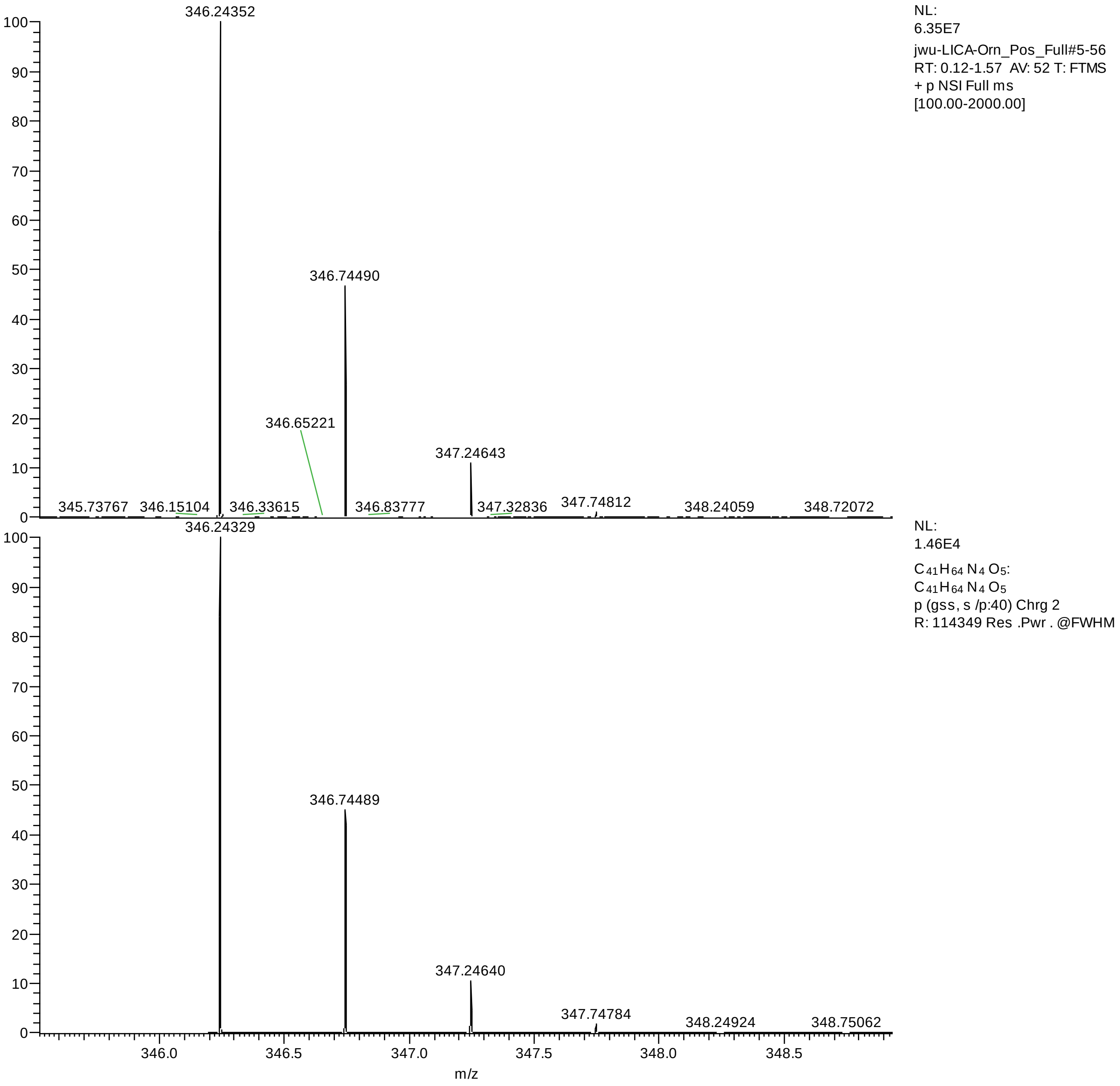

(*S*)-6-(((3*R*,5*R*,8*R*,9*S*,10*S*,13*R*,14*S*,17*R*)-17-((*R*)-5-(((*S*)-3-(1*H*-Indol-3-yl)-1-methoxy-1-oxopropan-2-yl)amino)-5-oxopentan-2-yl)-10,13-dimethylhexadecahydro-1*H*-cyclopenta[*a*]phenanthren-3-yl)oxy)-6-oxohexane-1,5-diaminium **(16d)**

Full spectrum

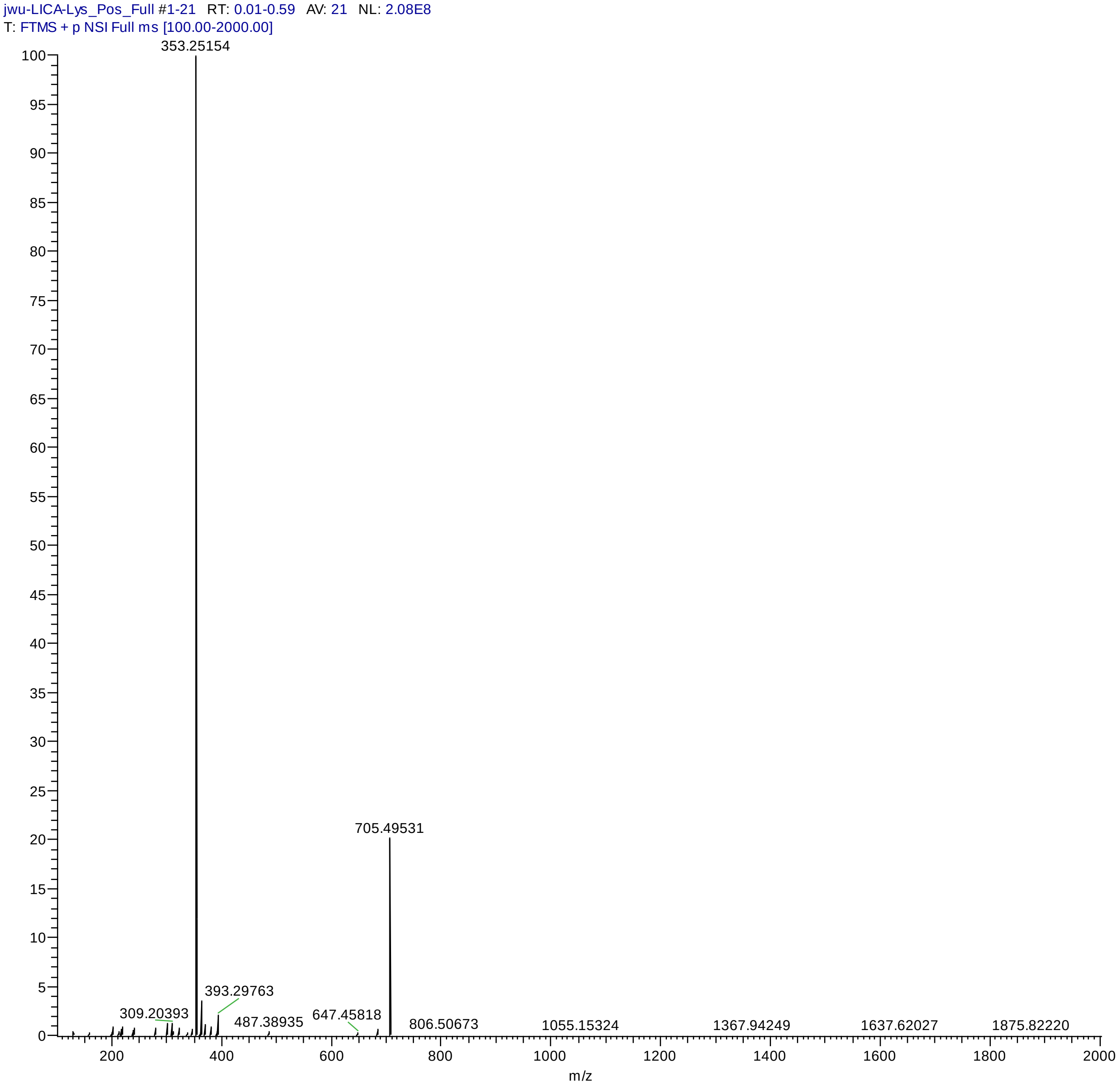

Zoomed spectrum (Top spectrum: measured mass; bottom spectrum: calculated mass)

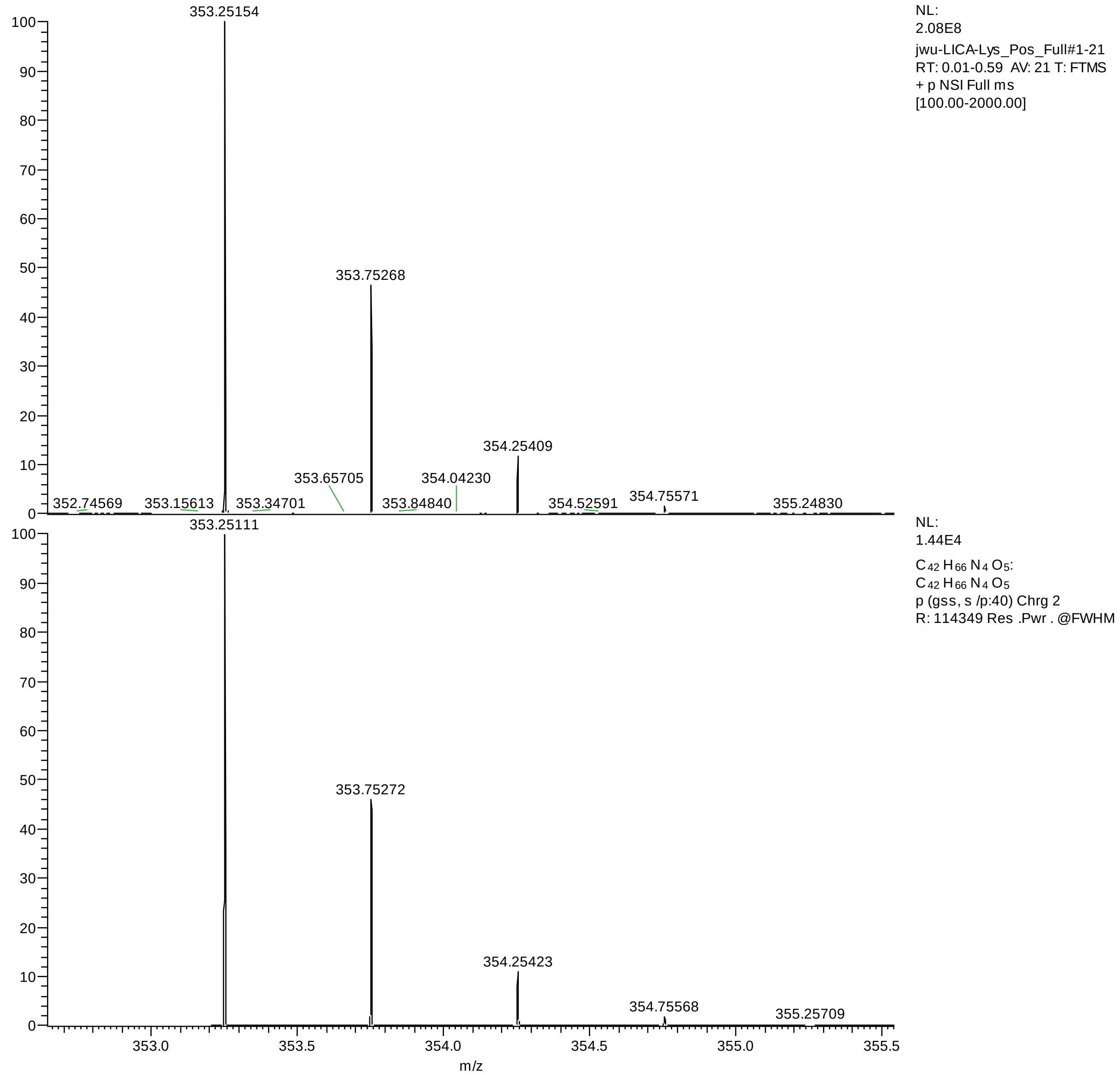

2,2'-(((3*R*,5*S*,7*R*,8*R*,9*S*,10*S*,13*R*,14*S*,17*R*)-17-((*R*)-5-(((*S*)-3-(1*H*-Indol-3-yl)-1-methoxy-1-oxopropan-2-yl)amino)-5-oxopentan-2-yl)-10,13-dimethylhexadecahydro-1*H*-cyclopenta[*a*]phenanthrene-3,7-diyl)bis(oxy))bis(2-oxoethan-1-aminium) **(17a)**

Full spectrum

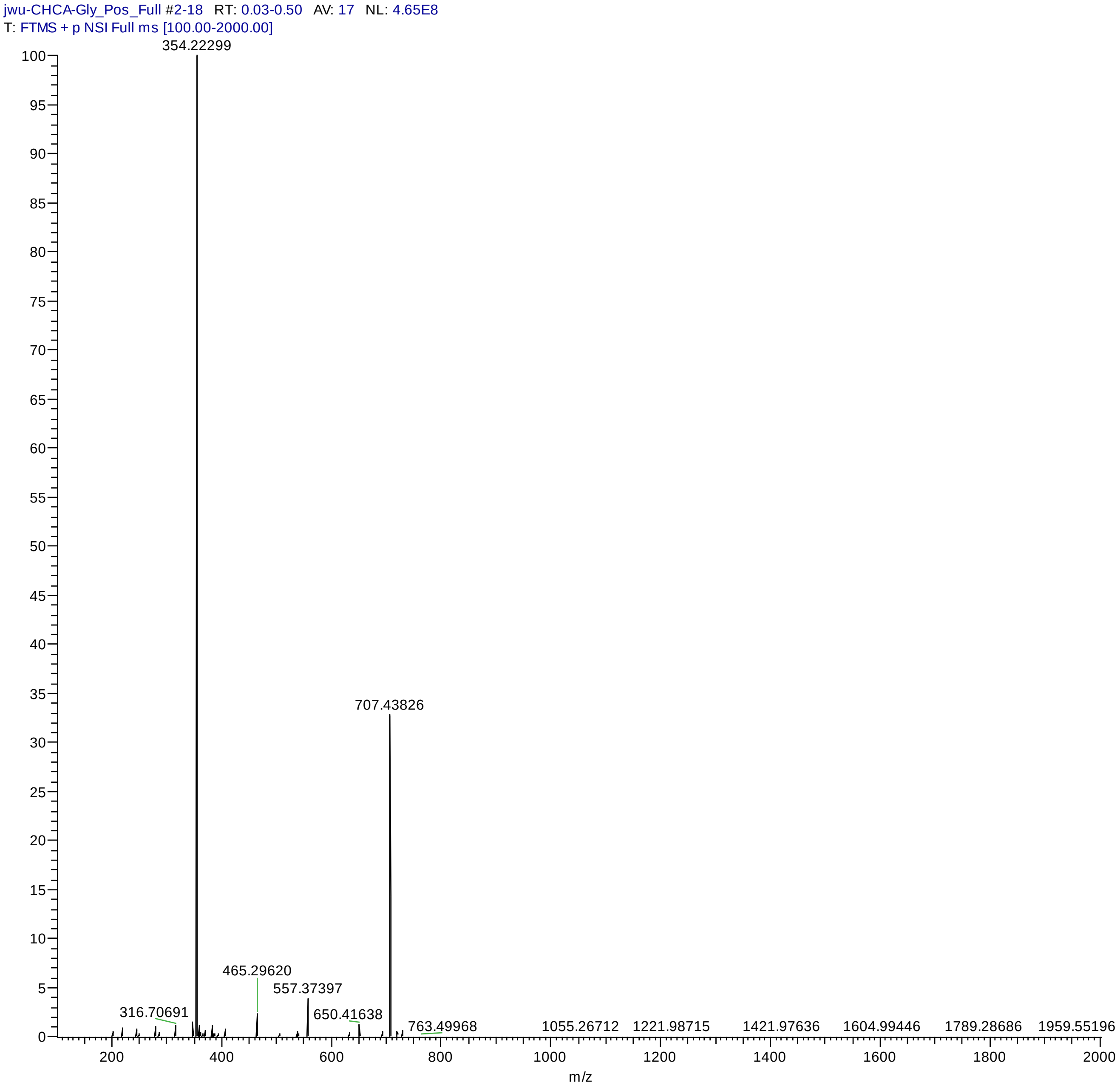

Zoomed spectrum (Top spectrum: measured mass; bottom spectrum: calculated mass)

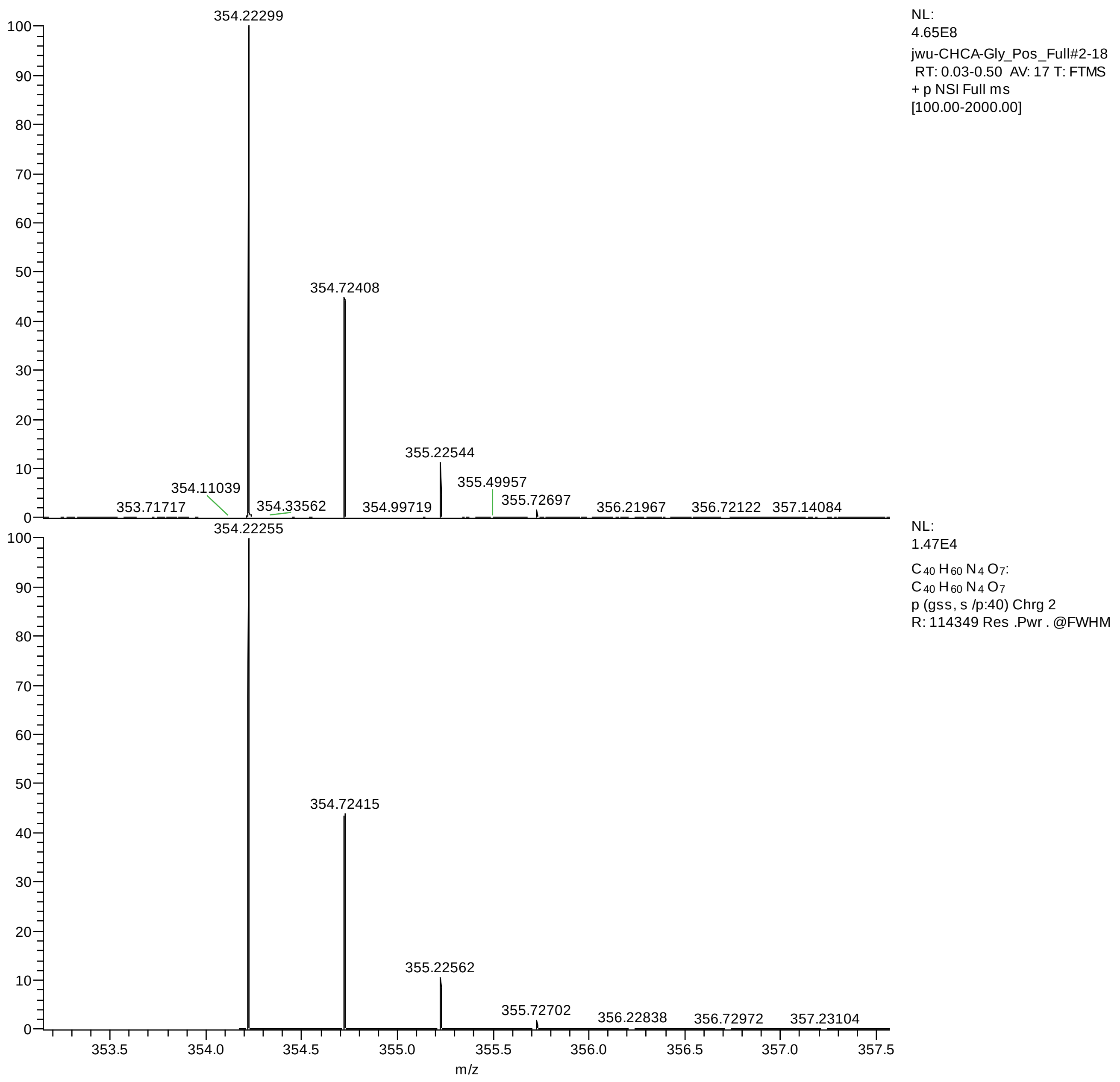

4,4'-(((3*R*,5*S*,7*R*,8*R*,9*S*,10*S*,13*R*,14*S*,17*R*)-17-((*R*)-5-(((*S*)-3-(1*H*-Indol-3-yl)-1-methoxy-1-oxopropan-2-yl)amino)-5-oxopentan-2-yl)-10,13-dimethylhexadecahydro-1*H*-cyclopenta[*a*]phenanthrene-3,7-diyl)bis(oxy))bis(4-oxobutan-1-aminium) **(17b)**

Full spectrum

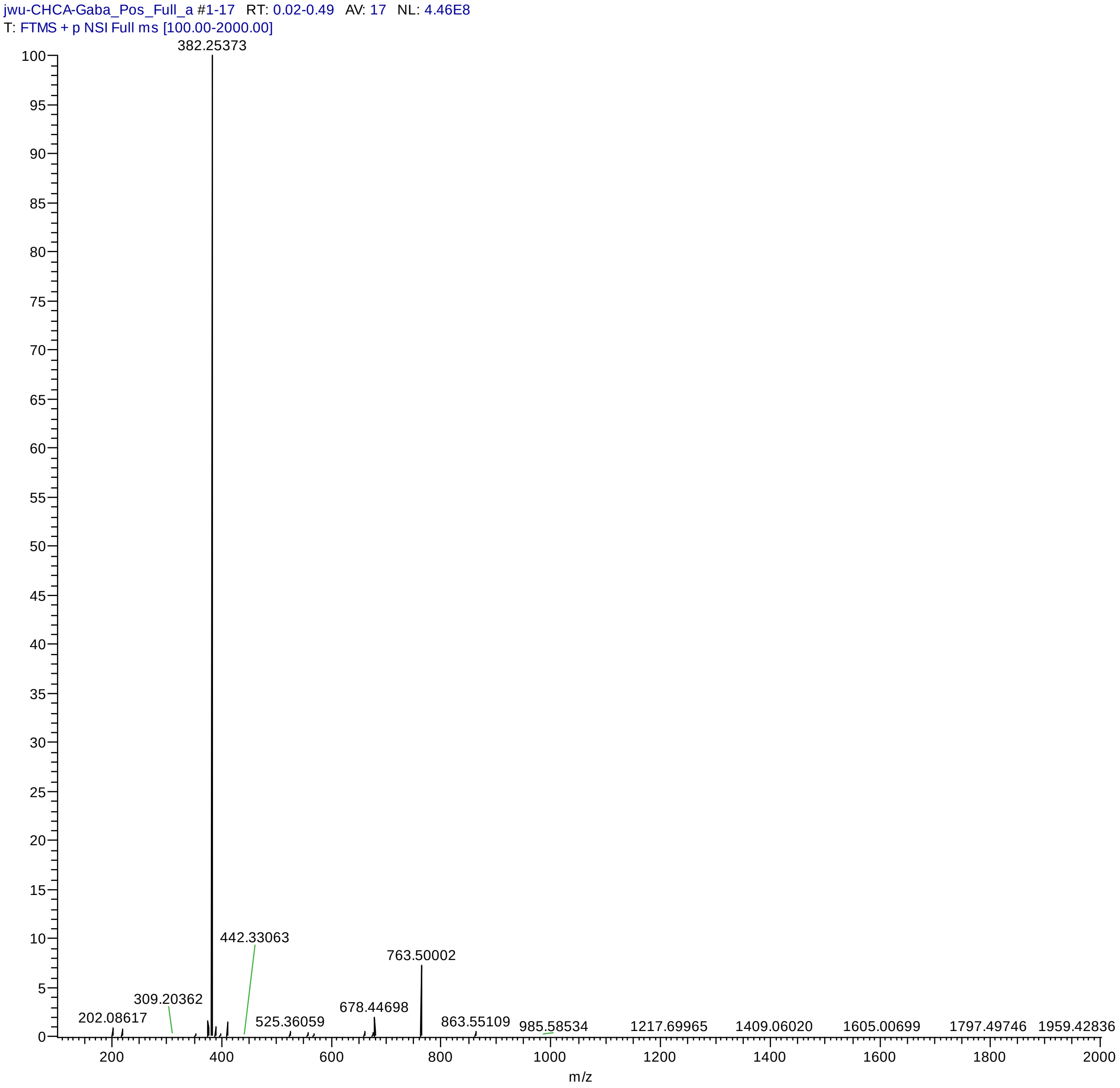

Zoomed spectrum (Top spectrum: measured mass; bottom spectrum: calculated mass)

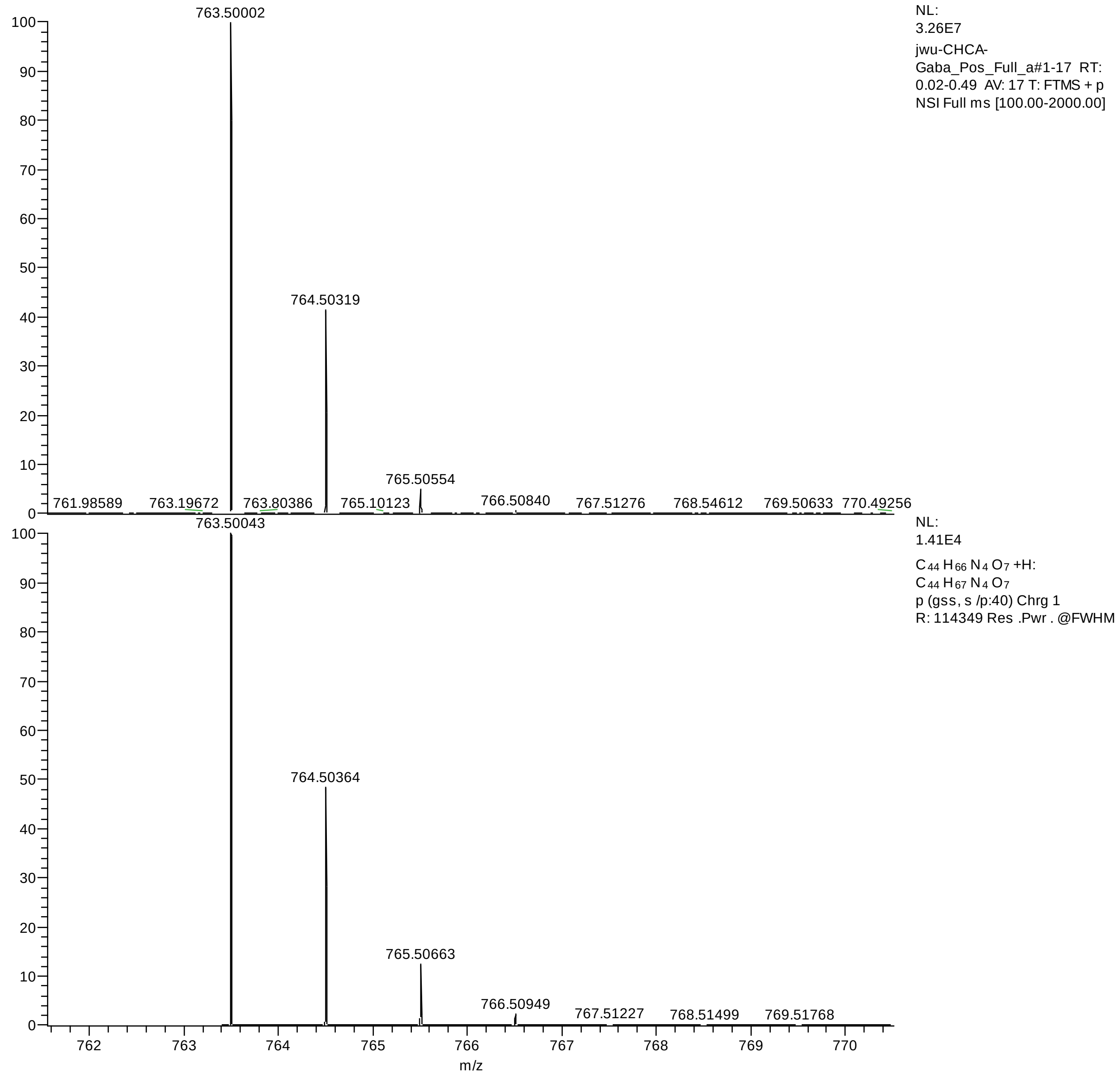

(4*S*,4'*S*)-5,5'-(((3*R*,5*S*,7*R*,8*R*,9*S*,10*S*,13*R*,14*S*,17*R*)-17-((*R*)-5-(((*S*)-3-(1*H*-Indol-3-yl)-1-methoxy-1-oxopropan-2-yl)amino)-5-oxopentan-2-yl)-10,13-dimethylhexadecahydro-1*H*-cyclopenta[*a*]phenanthrene-3,7-diyl)bis(oxy))bis(5-oxopentane-1,4-diaminium) **(17c)**

Full spectrum

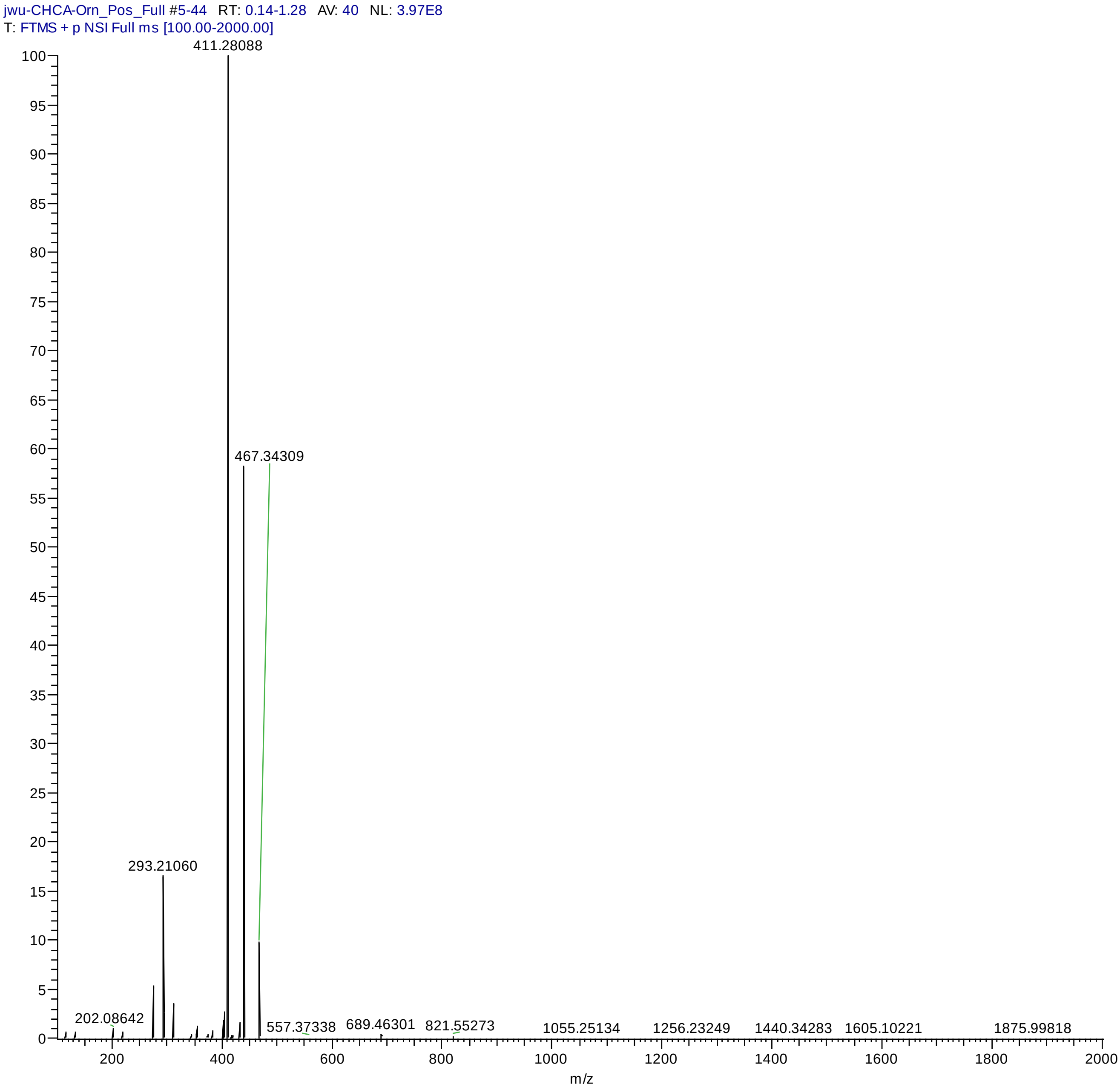

Zoomed spectrum (Top spectrum: measured mass; bottom spectrum: calculated mass)

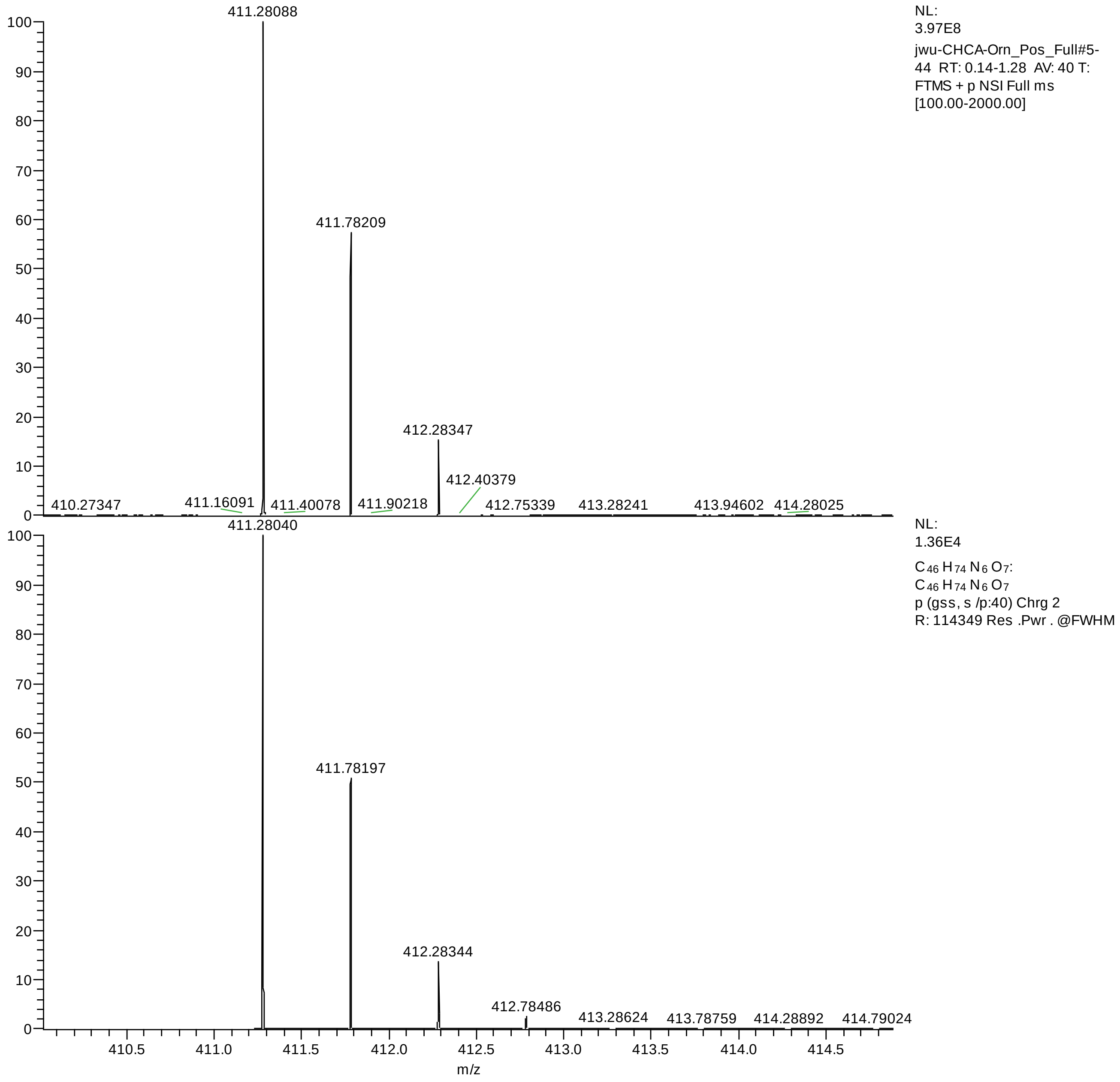

(5*S*,5'*S*)-6,6'-(((3*R*,5*S*,7*R*,8*R*,9*S*,10*S*,13*R*,14*S*,17*R*)-17-((*R*)-5-(((*S*)-3-(1*H*-Indol-3-yl)-1-methoxy-1-oxopropan-2-yl)amino)-5-oxopentan-2-yl)-10,13-dimethylhexadecahydro-1*H*-cyclopenta[*a*]phenanthrene-3,7-diyl)bis(oxy))bis(6-oxohexane-1,5-diaminium) **(17d)**

Full spectrum

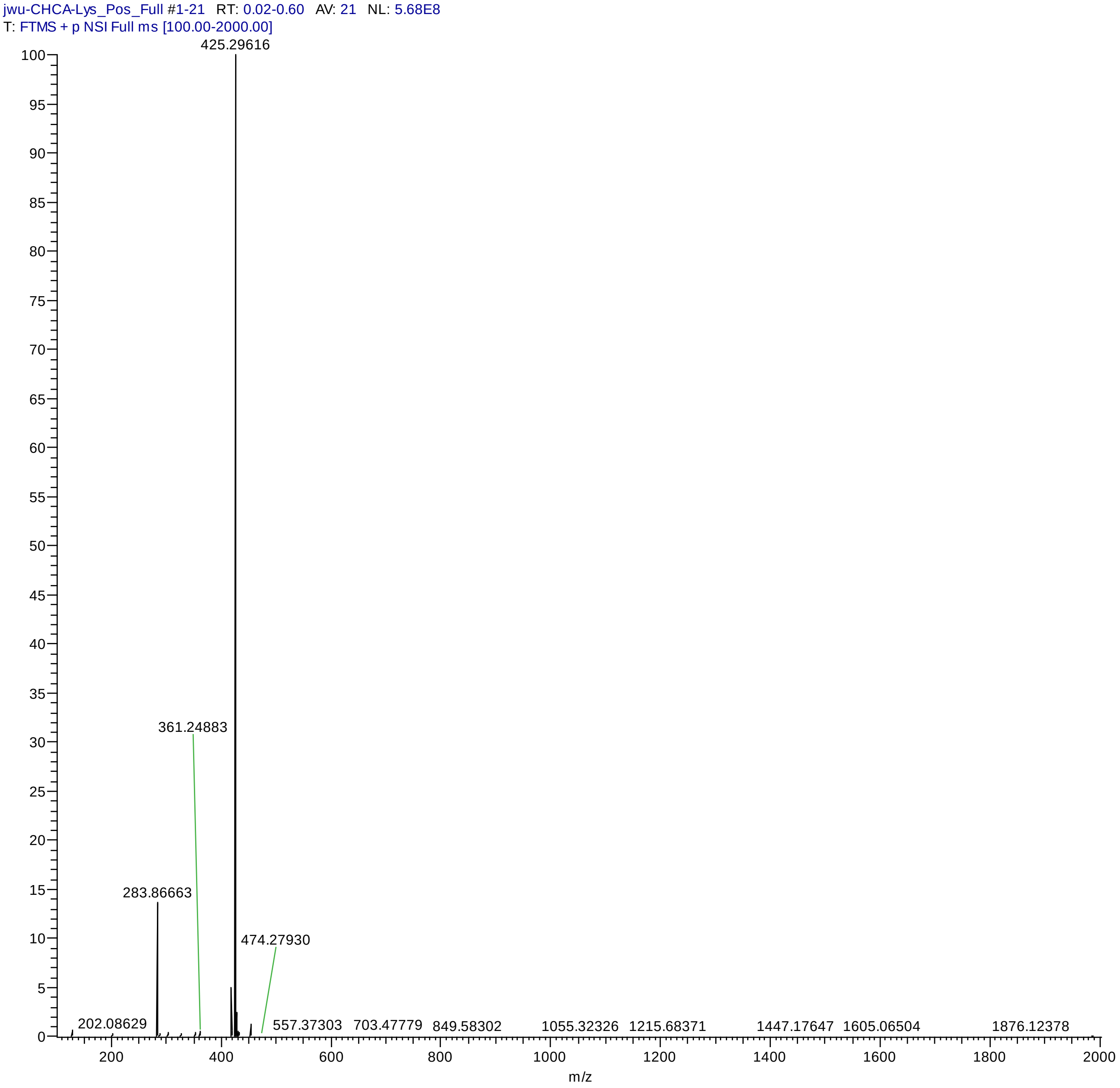

Zoomed spectrum (Top spectrum: measured mass; bottom spectrum: calculated mass)

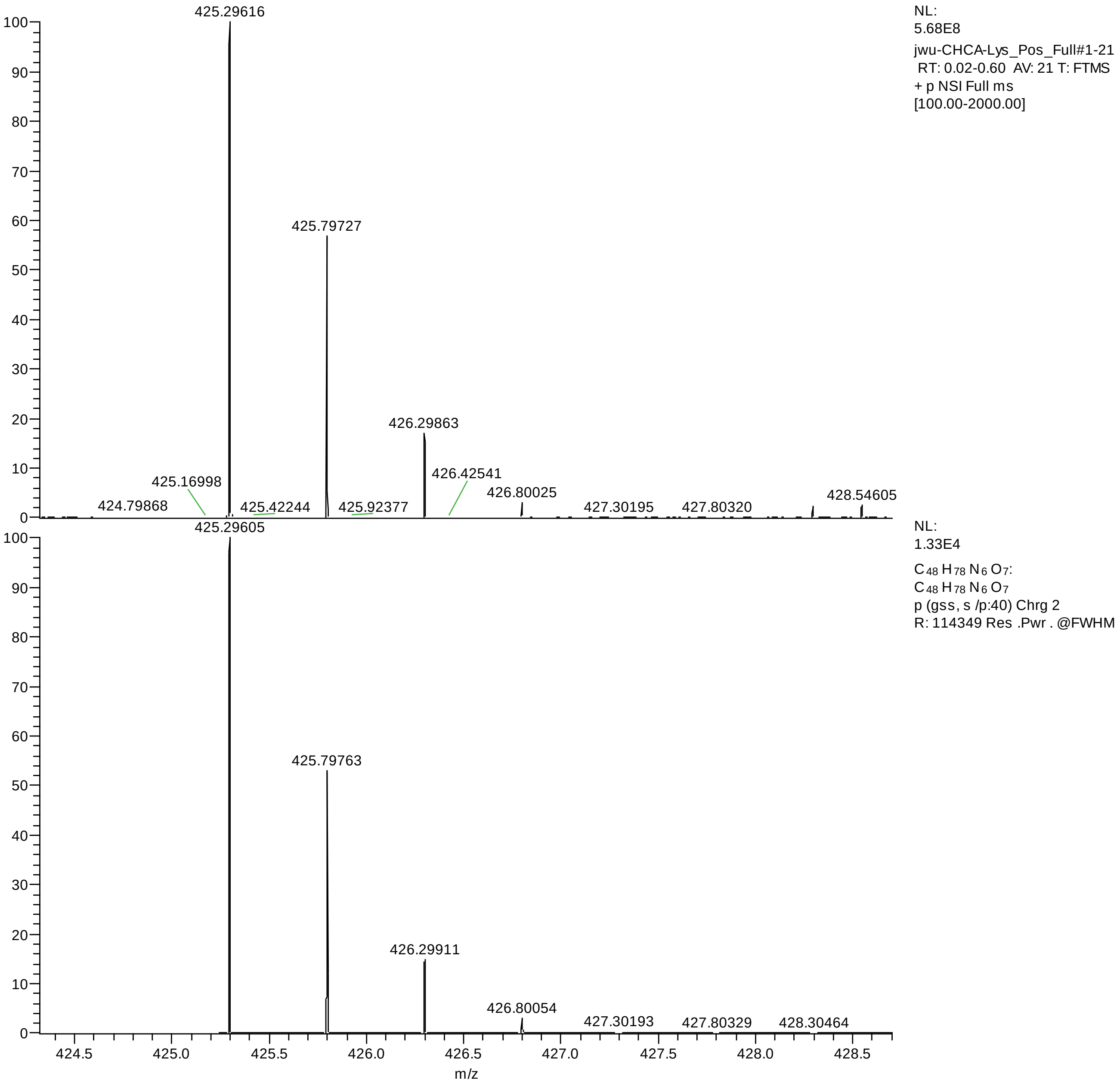

2,2'-(((3*R*,5*R*,8*R*,9*S*,10*S*,12*S*,13*R*,14*S*,17*R*)-17-((*R*)-5-(((*S*)-3-(1*H*-Indol-3-yl)-1-methoxy-1-oxopropan-2-yl)amino)-5-oxopentan-2-yl)-10,13-dimethylhexadecahydro-1*H*-cyclopenta[*a*]phenanthrene-3,12-diyl)bis(oxy))bis(2-oxoethan-1-aminium) **(18a)**

Full spectrum

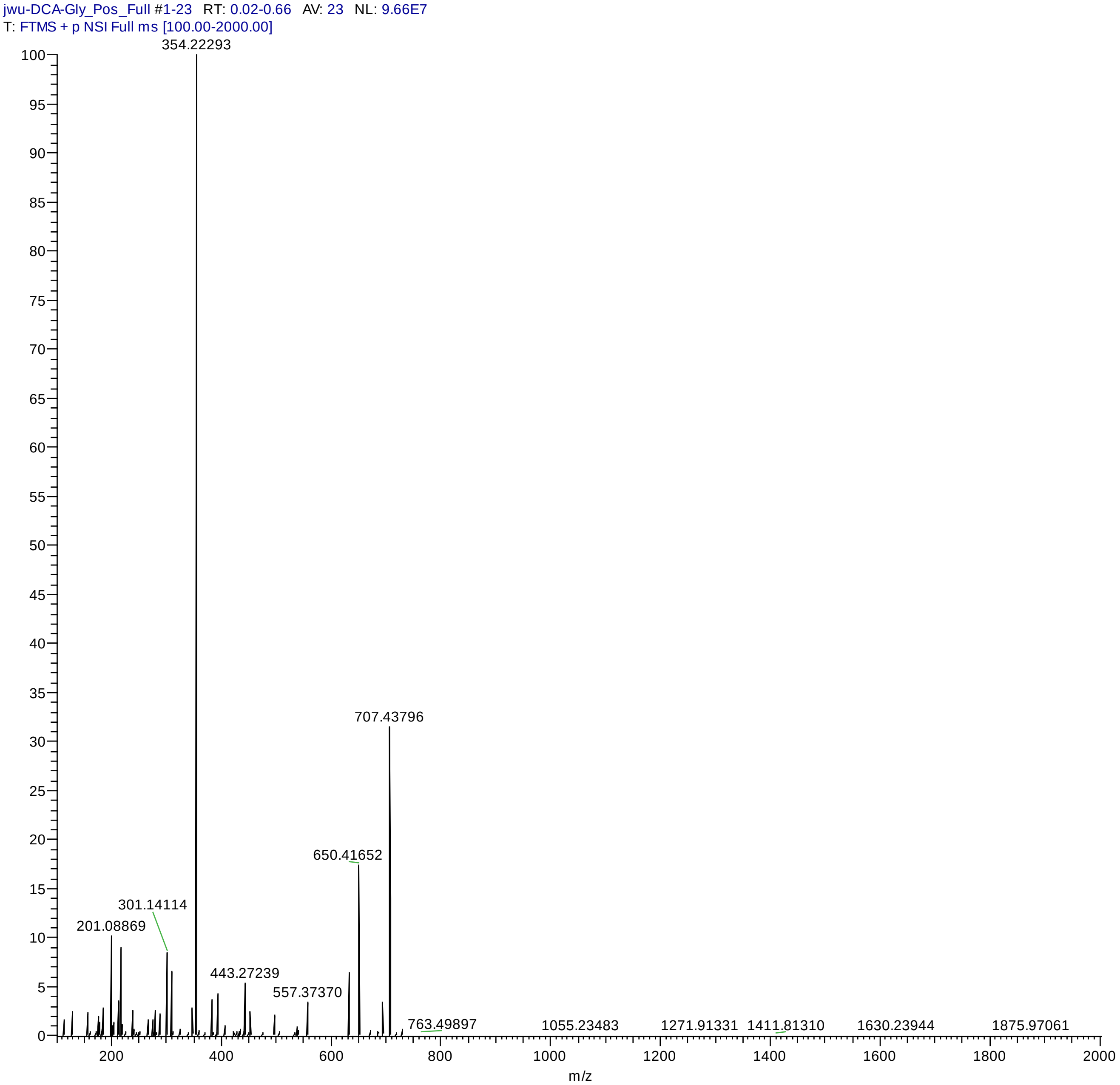

Zoomed spectrum (Top spectrum: measured mass; bottom spectrum: calculated mass)

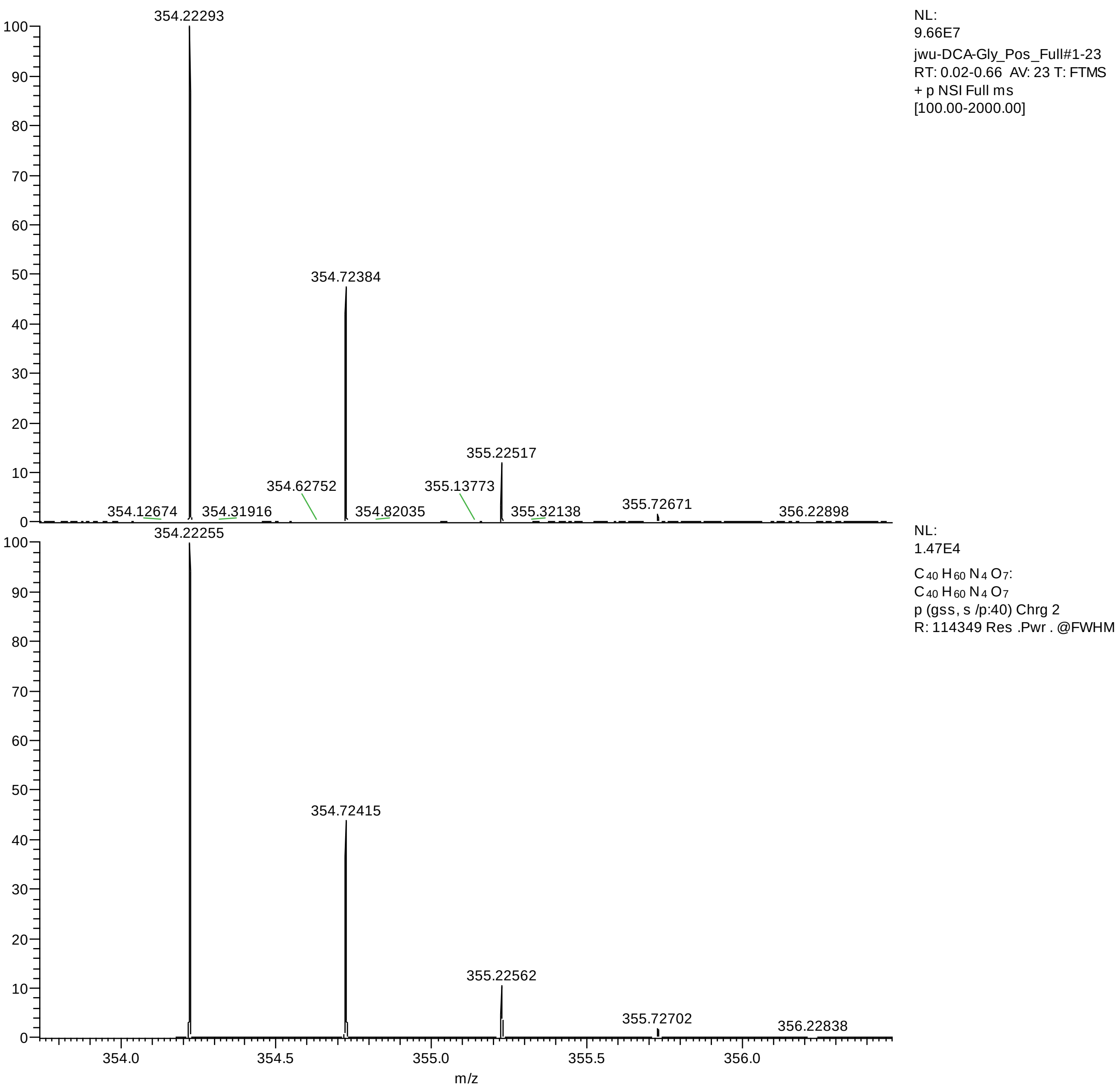

2,2'-(((3*R*,5*S*,7*R*,8*R*,9*S*,10*S*,12*S*,13*R*,14*S*,17*R*)-17-((*R*)-5-(((*S*)-3-(1*H*-Indol-3-yl)-1-methoxy-1-oxopropan-2-yl)amino)-5-oxopentan-2-yl)-3-(2-aminoacetoxy)-10,13-dimethylhexadecahydro-1*H*-cyclopenta[*a*]phenanthrene-7,12-diyl)bis(oxy))bis(2-oxoethan-1-aminium) **(19a)**

Full spectrum

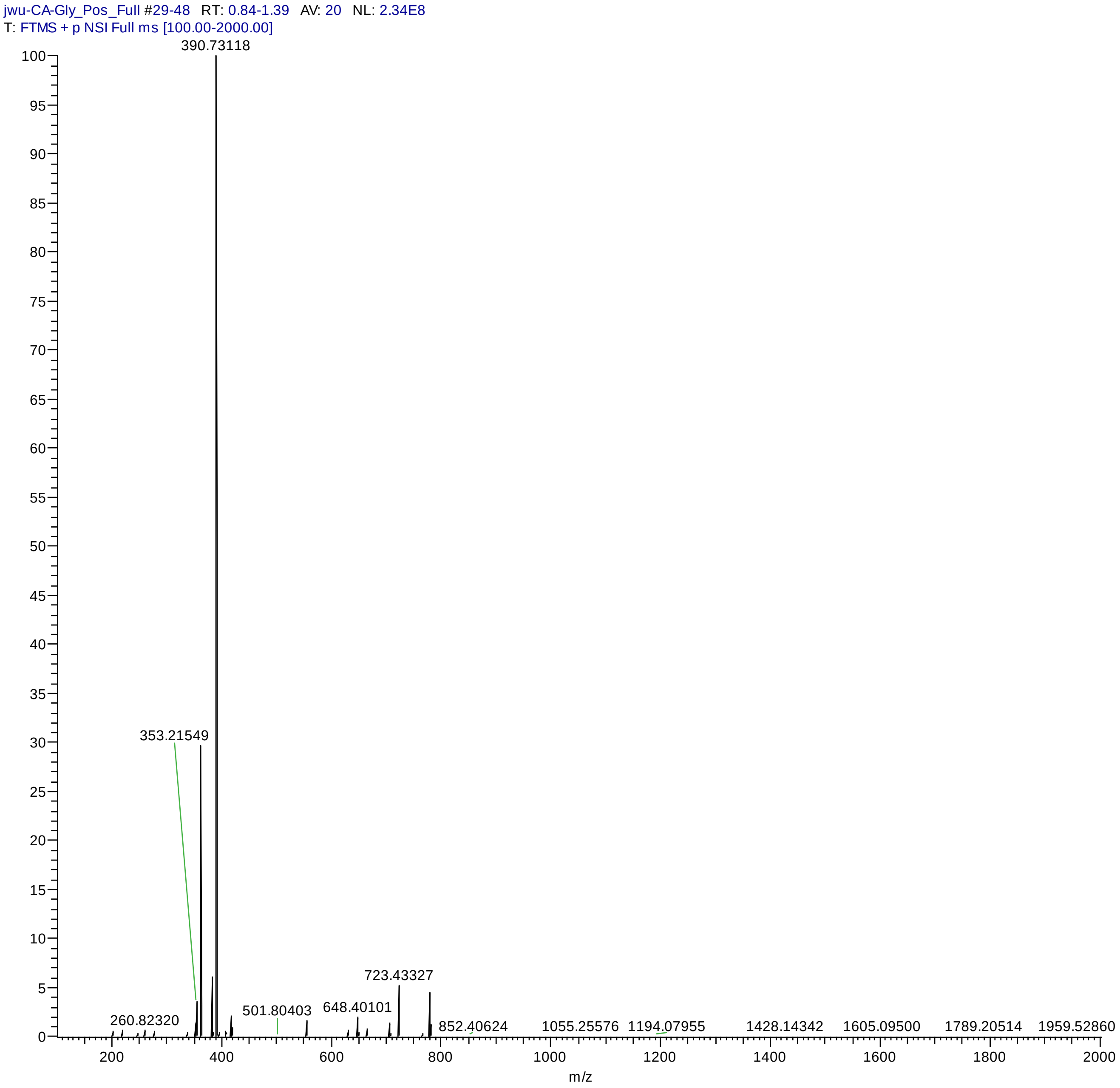

Zoomed spectrum (Top spectrum: measured mass; bottom spectrum: calculated mass)

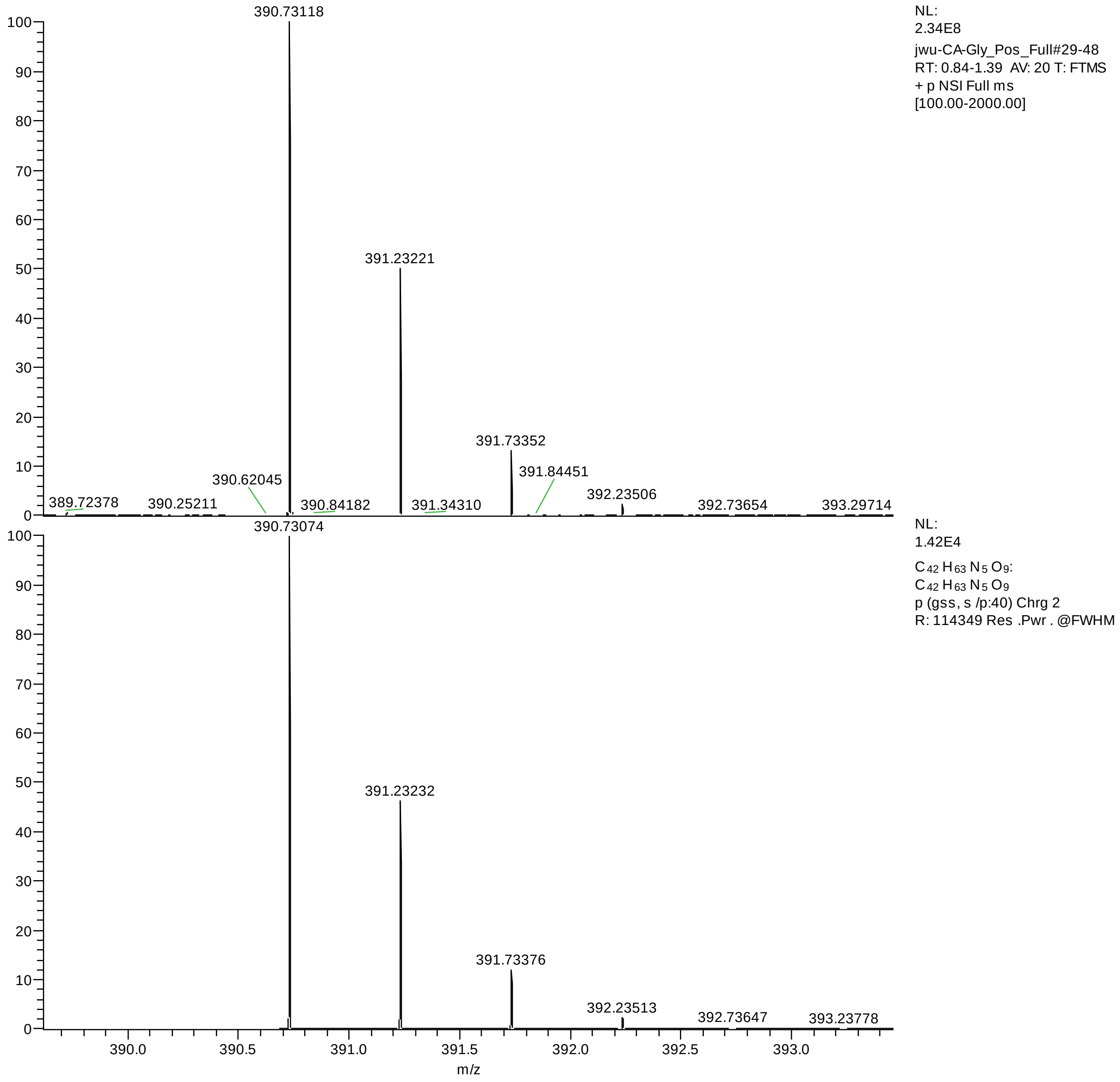

4,4',4''-(((3*R*,5*S*,7*R*,8*R*,9*S*,10*S*,12*S*,13*R*,14*S*,17*R*)-17-((*R*)-5-(((*S*)-3-(1*H*-Indol-3-yl)-1-methoxy-1-oxopropan-2-yl)amino)-5-oxopentan-2-yl)-10,13-dimethylhexadecahydro-1*H*-cyclopenta[*a*]phenanthrene-3,7,12-triyl)tris(oxy))tris(4-oxobutan-1-aminium) **(19b)**

Full spectrum

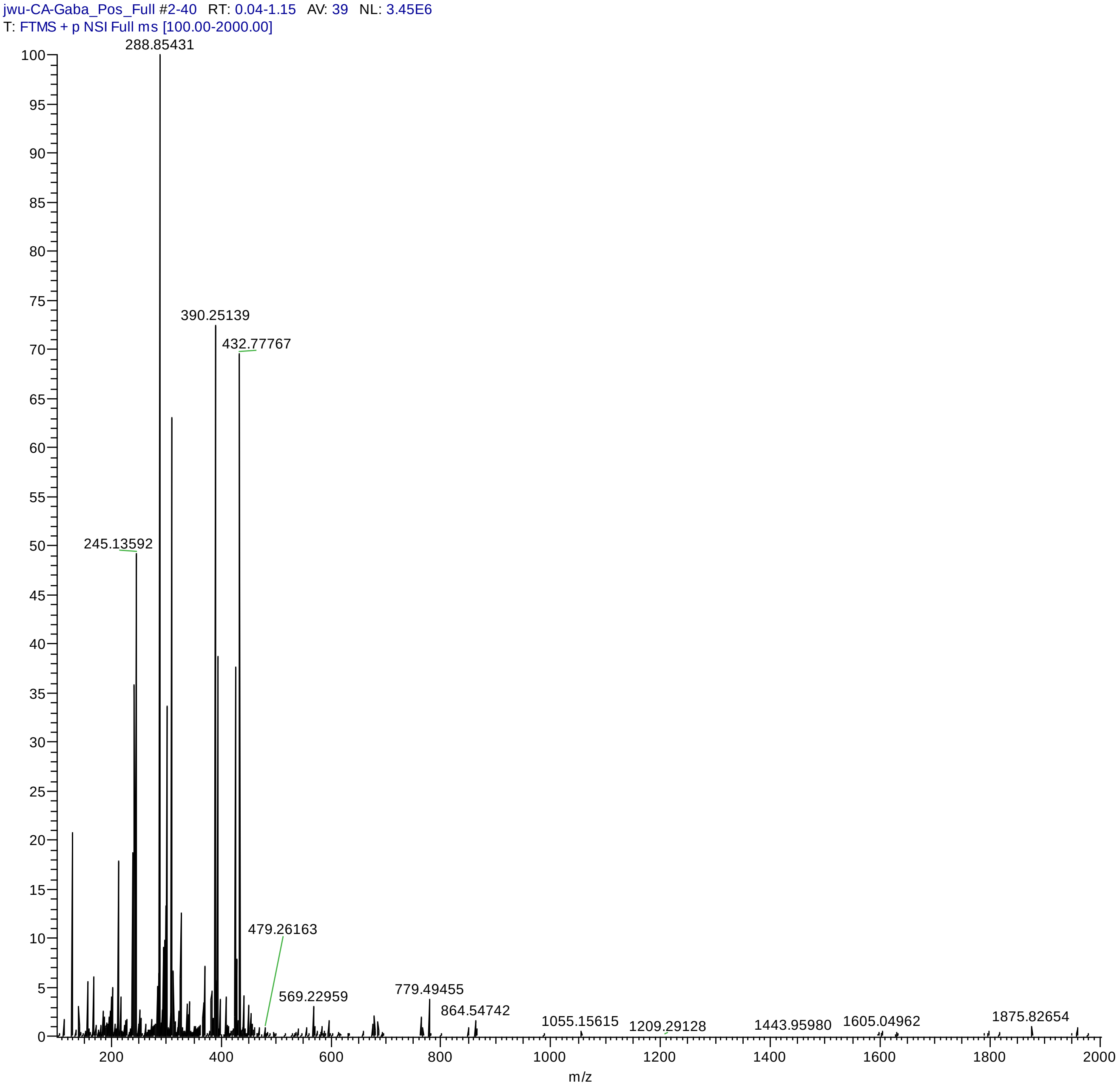

Zoomed spectrum (Top spectrum: measured mass; bottom spectrum: calculated mass)

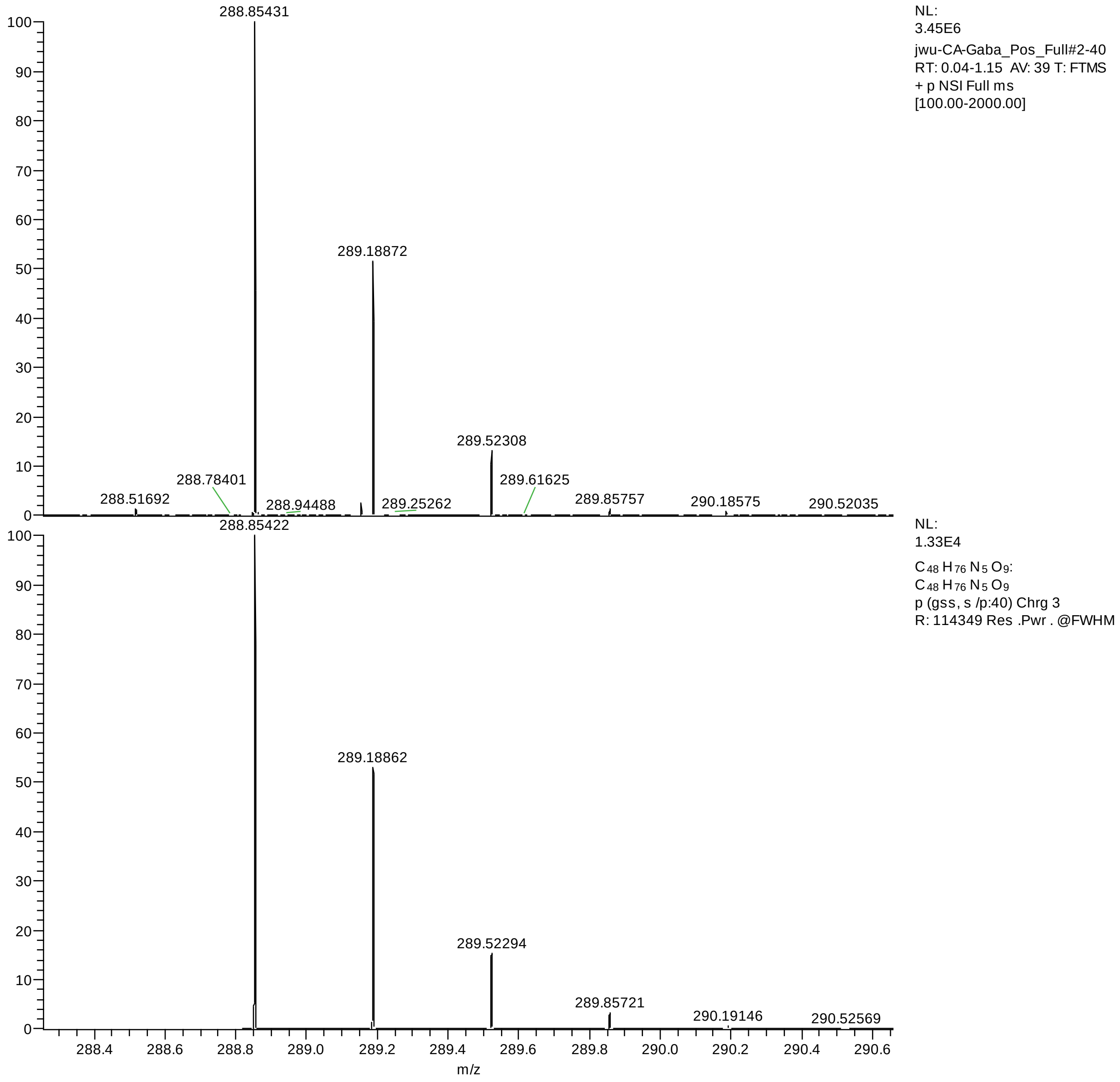

(4*S*,4'*S*,4''*S*)-5,5',5''-(((3*R*,5*S*,7*R*,8*R*,9*S*,10*S*,12*S*,13*R*,14*S*,17*R*)-17-((*R*)-5-(((*S*)-3-(1*H*-Indol-3-yl)-1-methoxy-1-oxopropan-2-yl)amino)-5-oxopentan-2-yl)-10,13-dimethylhexadecahydro-1*H*-cyclopenta[*a*]phenanthrene-3,7,12-triyl)tris(oxy))tris(5-oxopentane-1,4-diaminium) **(19c)**

Full spectrum

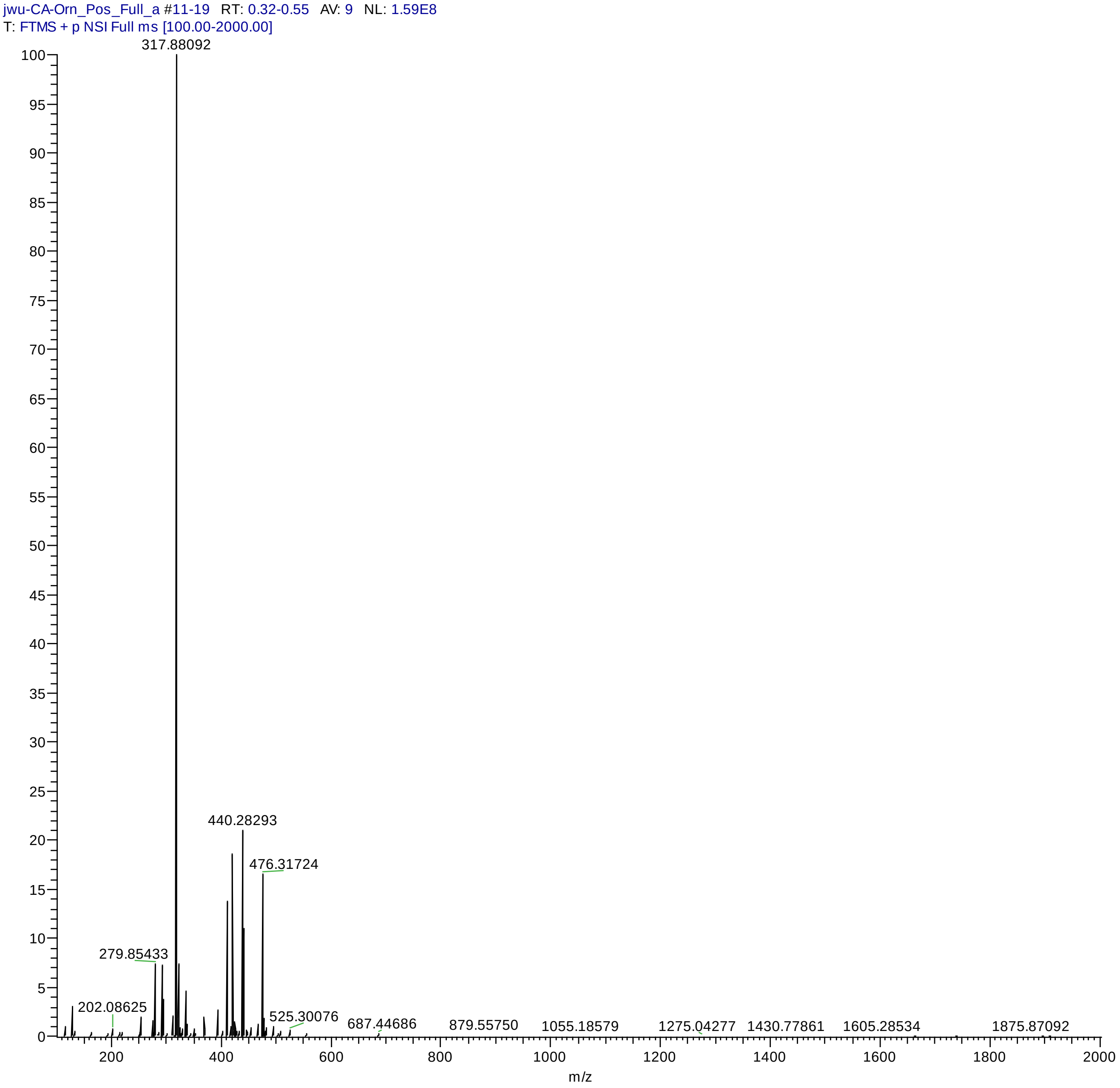

Zoomed spectrum (Top spectrum: measured mass; bottom spectrum: calculated mass)

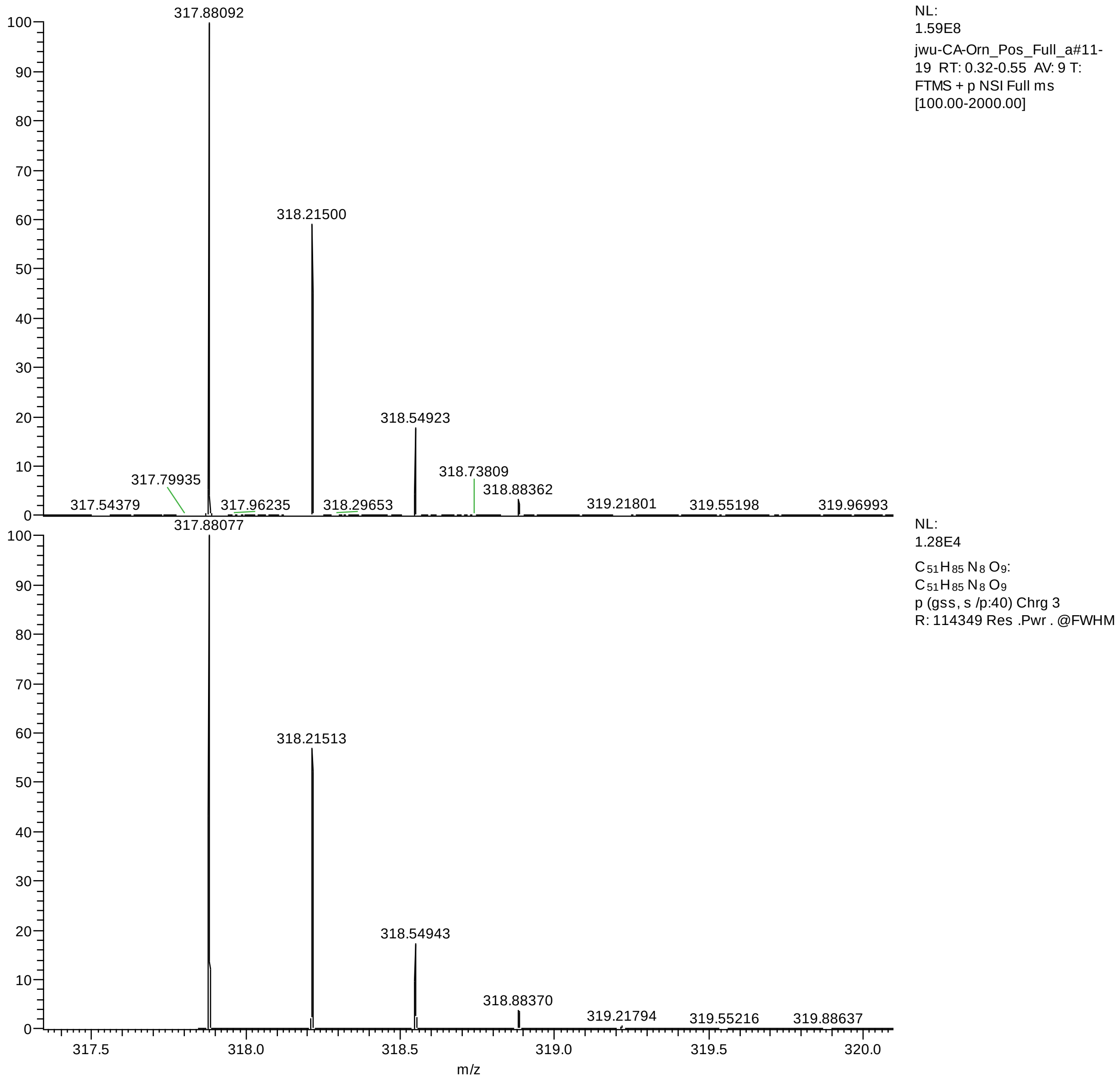

(5*S*,5'*S*,5''*S*)-6,6',6''-(((3*R*,5*S*,7*R*,8*R*,9*S*,10*S*,12*S*,13*R*,14*S*,17*R*)-17-((*R*)-5-(((*S*)-3-(1*H*-Indol-3-yl)-1-methoxy-1-oxopropan-2-yl)amino)-5-oxopentan-2-yl)-10,13-dimethylhexadecahydro-1*H*-cyclopenta[*a*]phenanthrene-3,7,12-triyl)tris(oxy))tris(6-oxohexane-1,5-diaminium) **(19d)**

Full spectrum

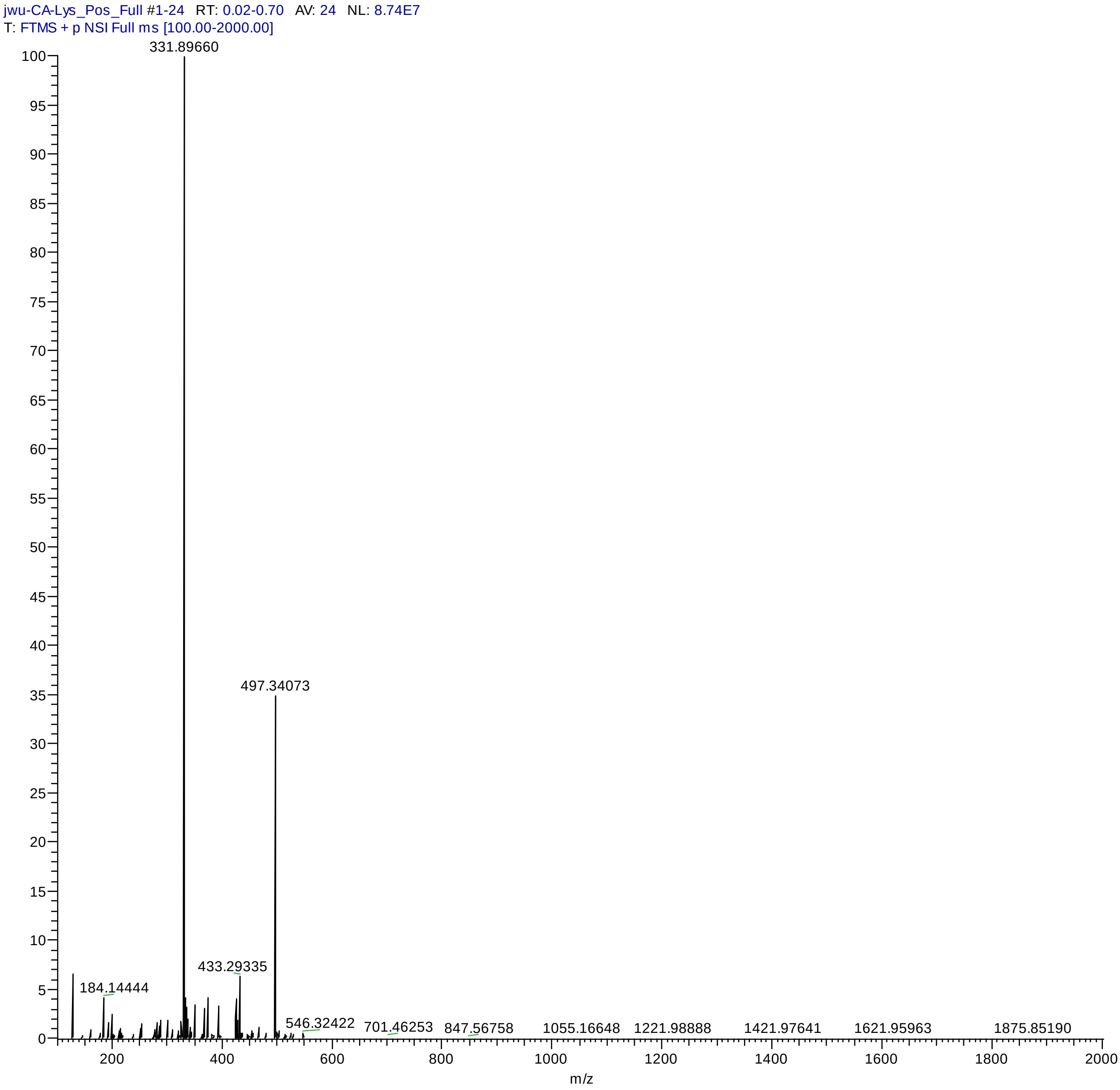

Zoomed spectrum (Top spectrum: measured mass; bottom spectrum: calculated mass)

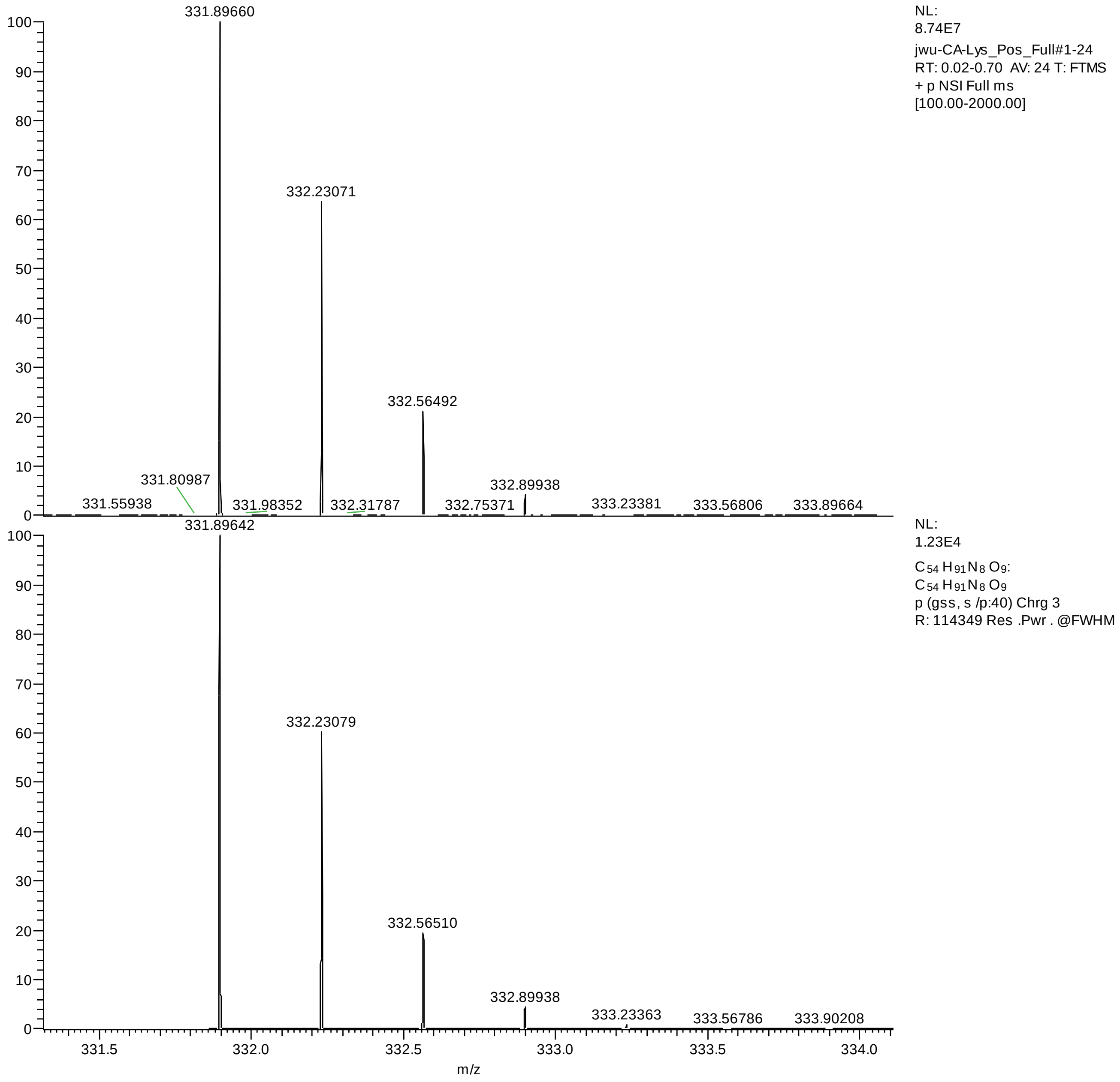

(4*S*,4'*S*)-5,5'-(((3*R*,5*S*,7*R*,8*R*,9*S*,10*S*,13*R*,14*S*,17*R*)-17-((*R*)-5-(((*S*)-1-Methoxy-1-oxo-3-phenylpropan-2-yl)amino)-5-oxopentan-2-yl)-10,13-dimethylhexadecahydro-1*H*-cyclopenta[*a*]phenanthrene-3,7-diyl)bis(oxy))bis(5-oxopentane-1,4-diaminium) **(20)**

Full spectrum

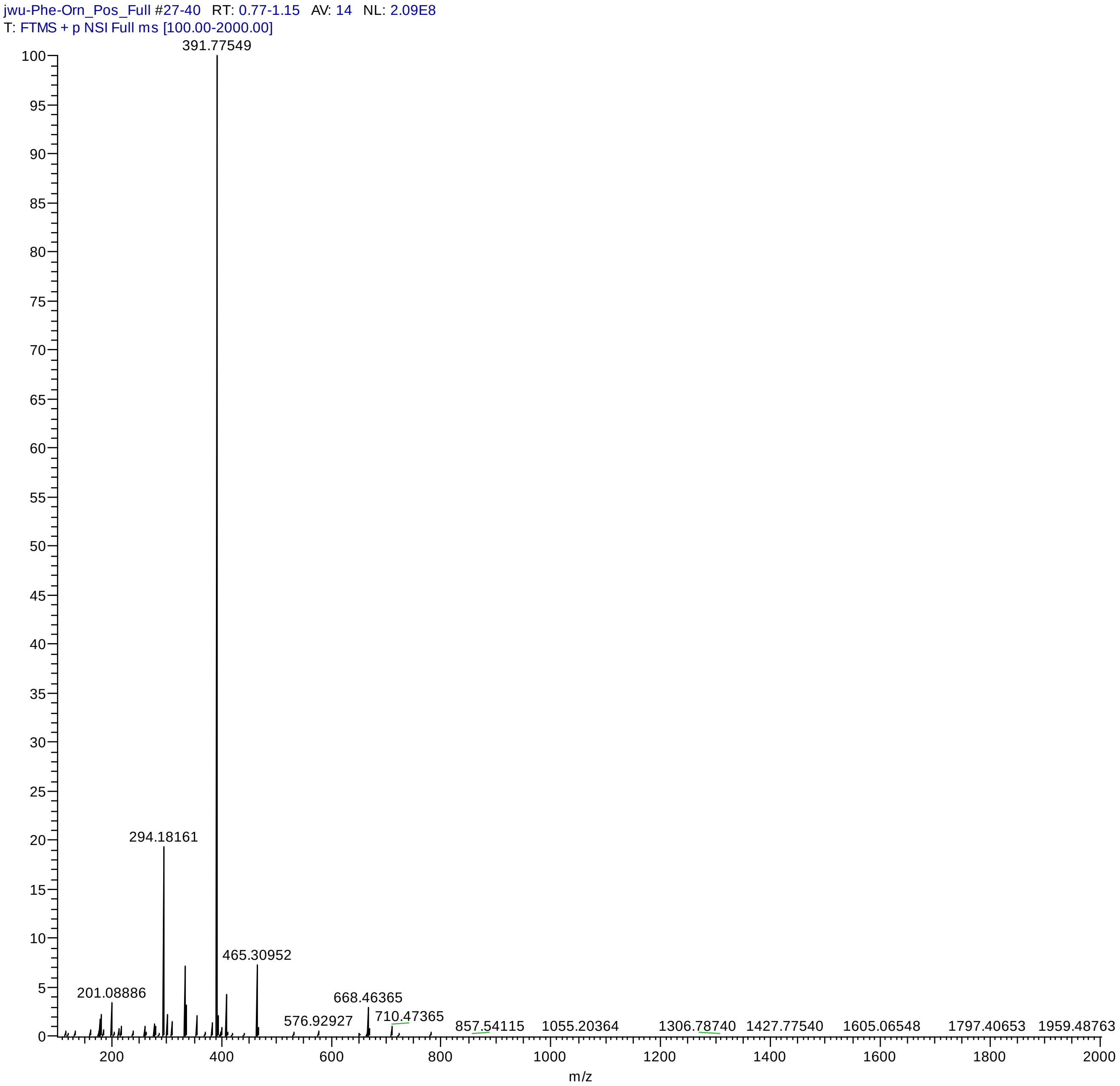

Zoomed spectrum (Top spectrum: measured mass; bottom spectrum: calculated mass)

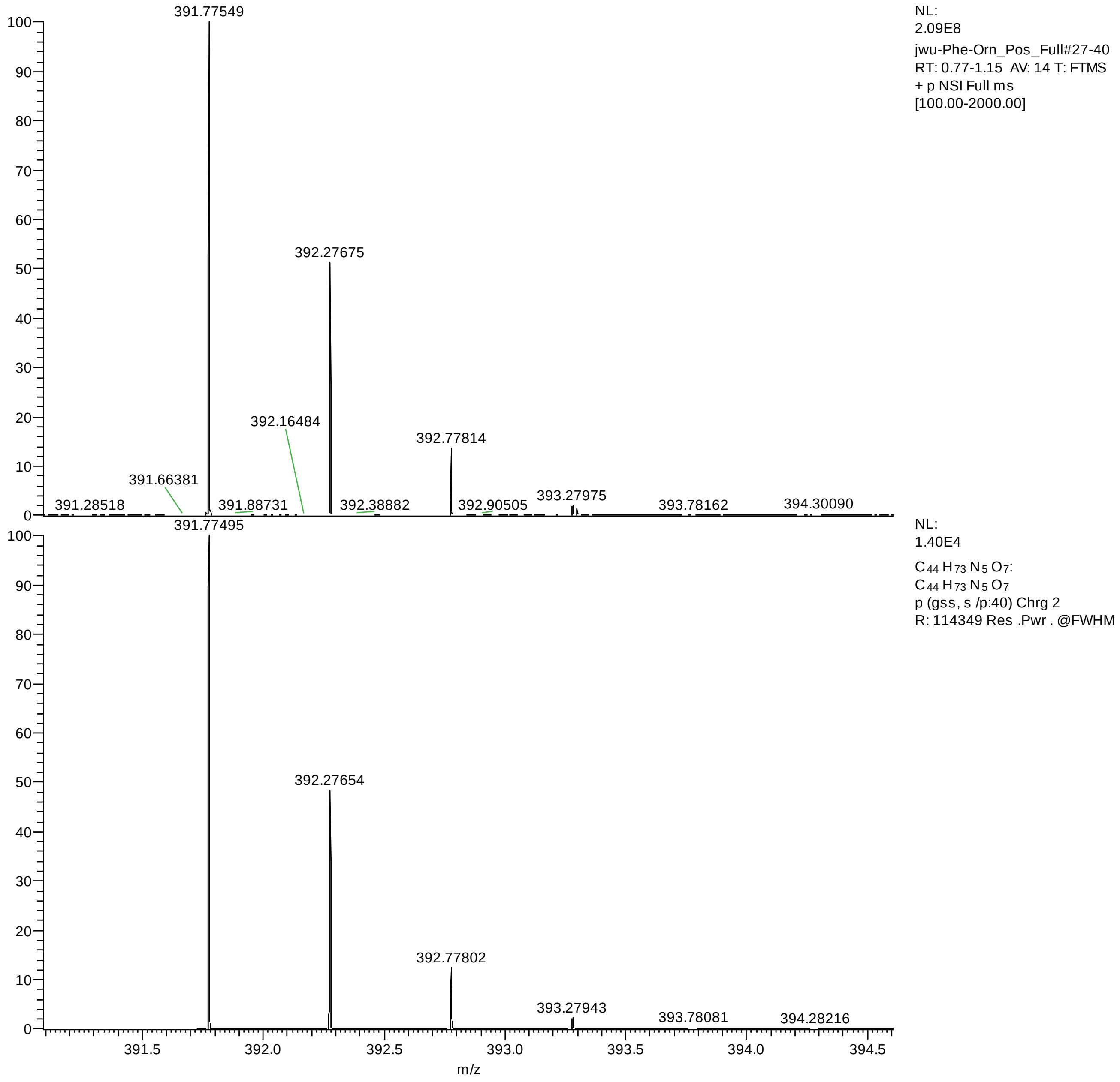

(4*S*,4'*S*)-5,5'-(((3*R*,5*S*,7*R*,8*R*,9*S*,10*S*,13*R*,14*S*,17*R*)-17-((*R*)-5-(Benzylamino)-5-oxopentan-2-yl)-10,13-dimethylhexadecahydro-1*H*-cyclopenta[*a*]phenanthrene-3,7-diyl)bis(oxy))bis(5-oxopentane-1,4-diaminium) **(21)**

Full spectrum

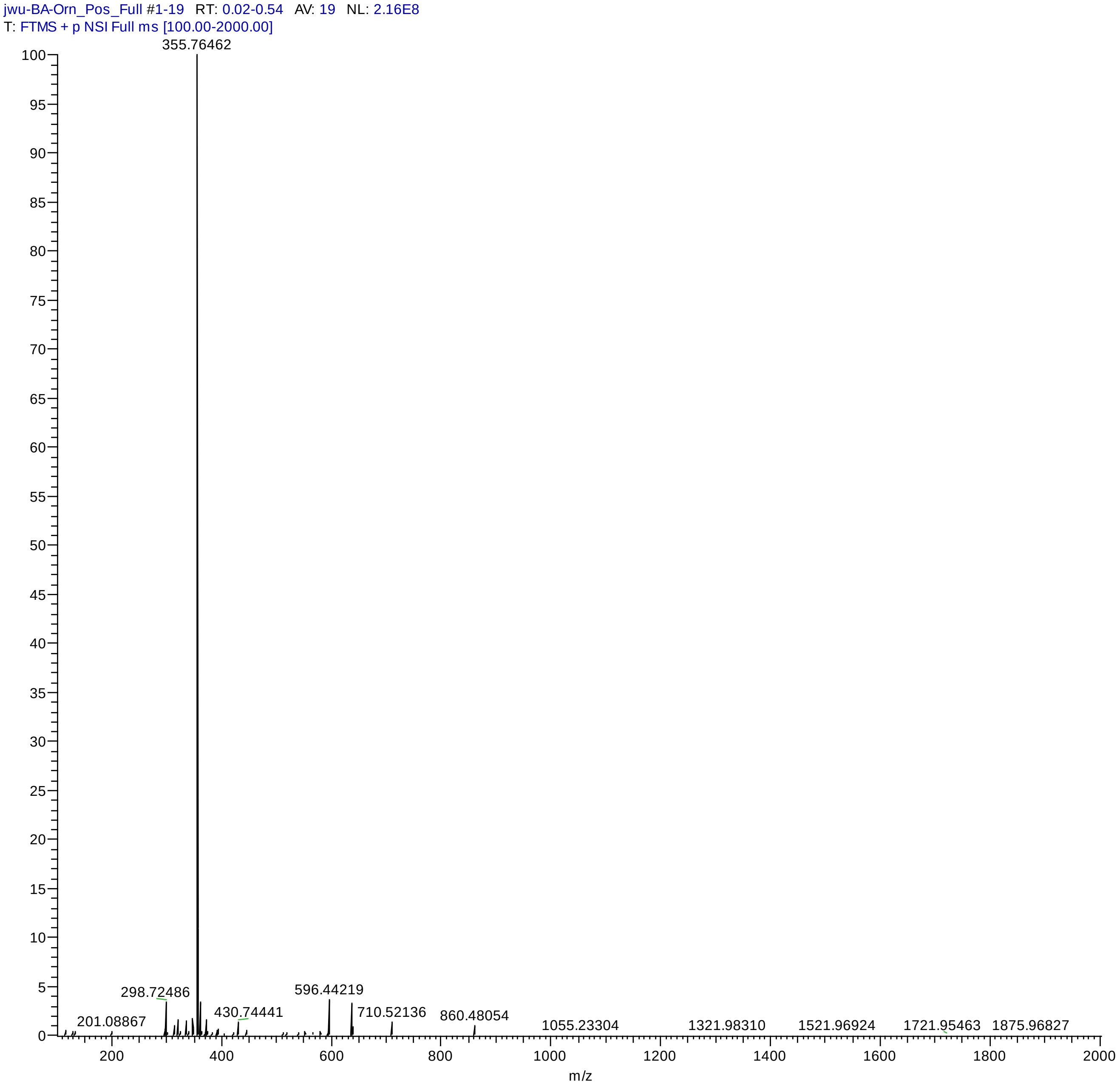

Zoomed spectrum (Top spectrum: measured mass; bottom spectrum: calculated mass)

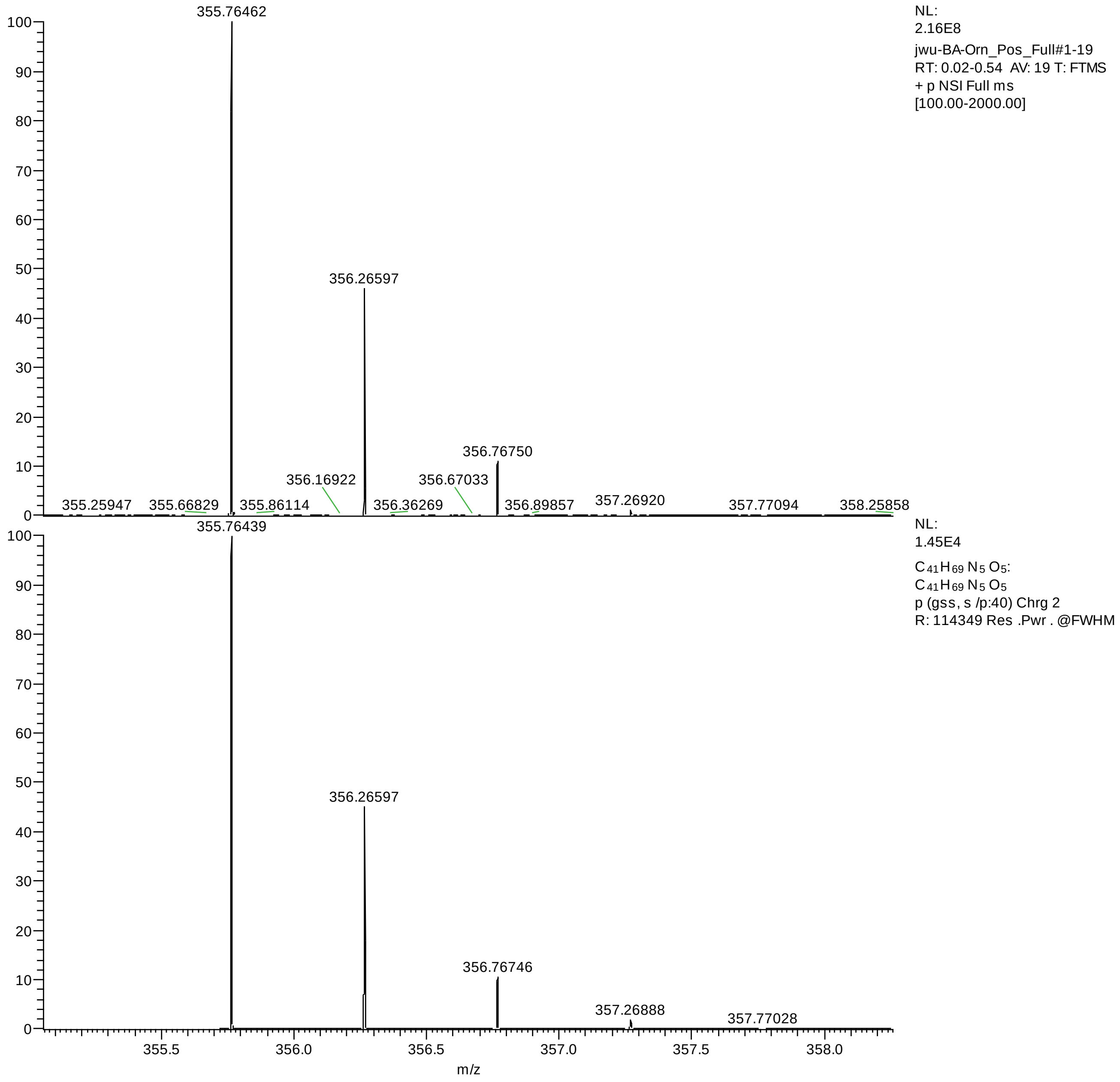

((4-(((3*R*,5*R*,8*R*,9*S*,10*S*,13*R*,14*S*,17*R*)-17-((*R*)-5-(((*S*)-3-(1*H*-Indol-3-yl)-1-methoxy-1-oxopropan-2-yl)amino)-5-oxopentan-2-yl)-10,13-dimethylhexadecahydro-1*H*-cyclopenta[*a*]phenanthren-3-yl)oxy)-4-oxobutyl)amino)(amino)methaniminium **(23)**

Full spectrum

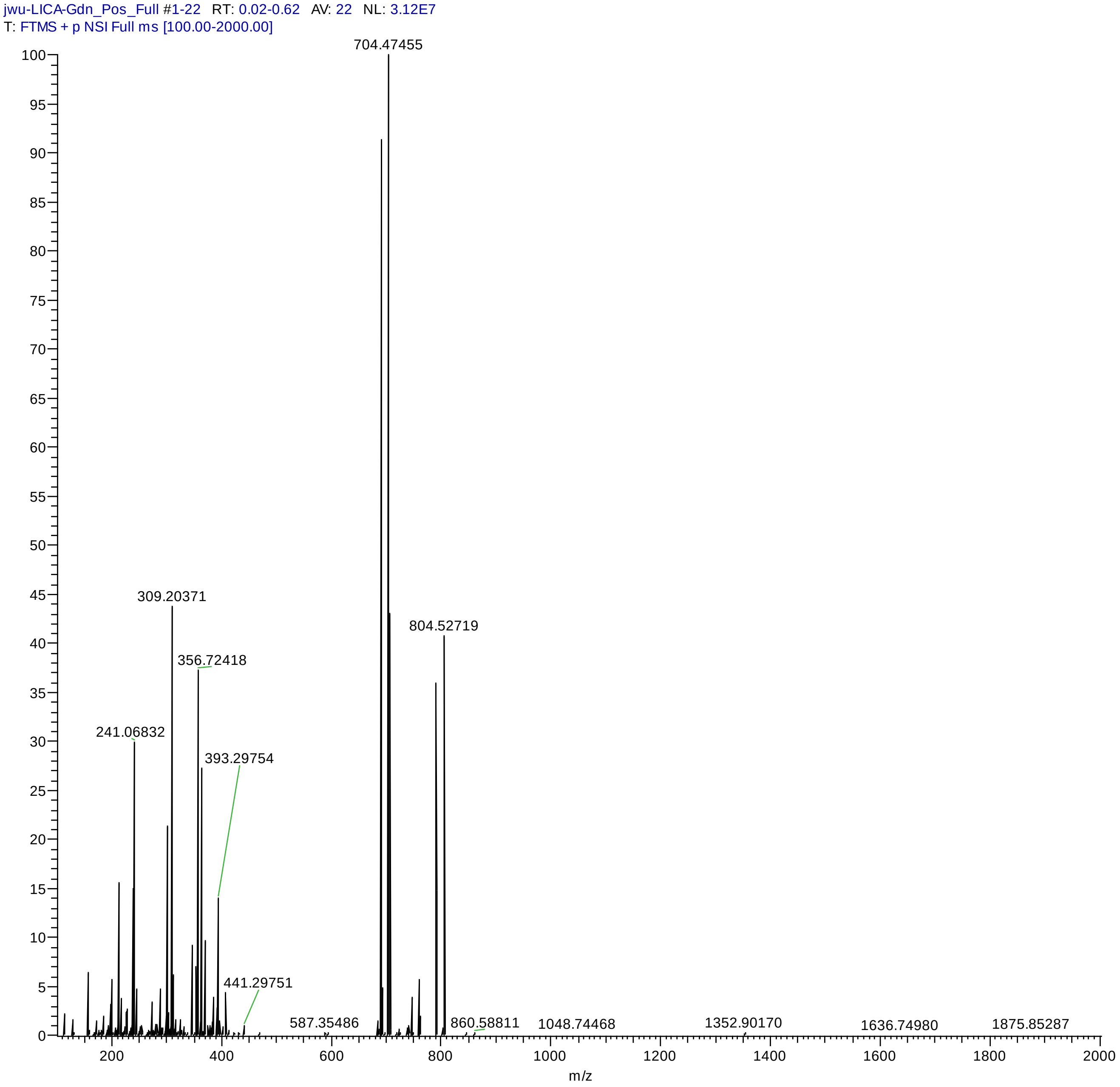

Zoomed spectrum (Top spectrum: measured mass; bottom spectrum: calculated mass)

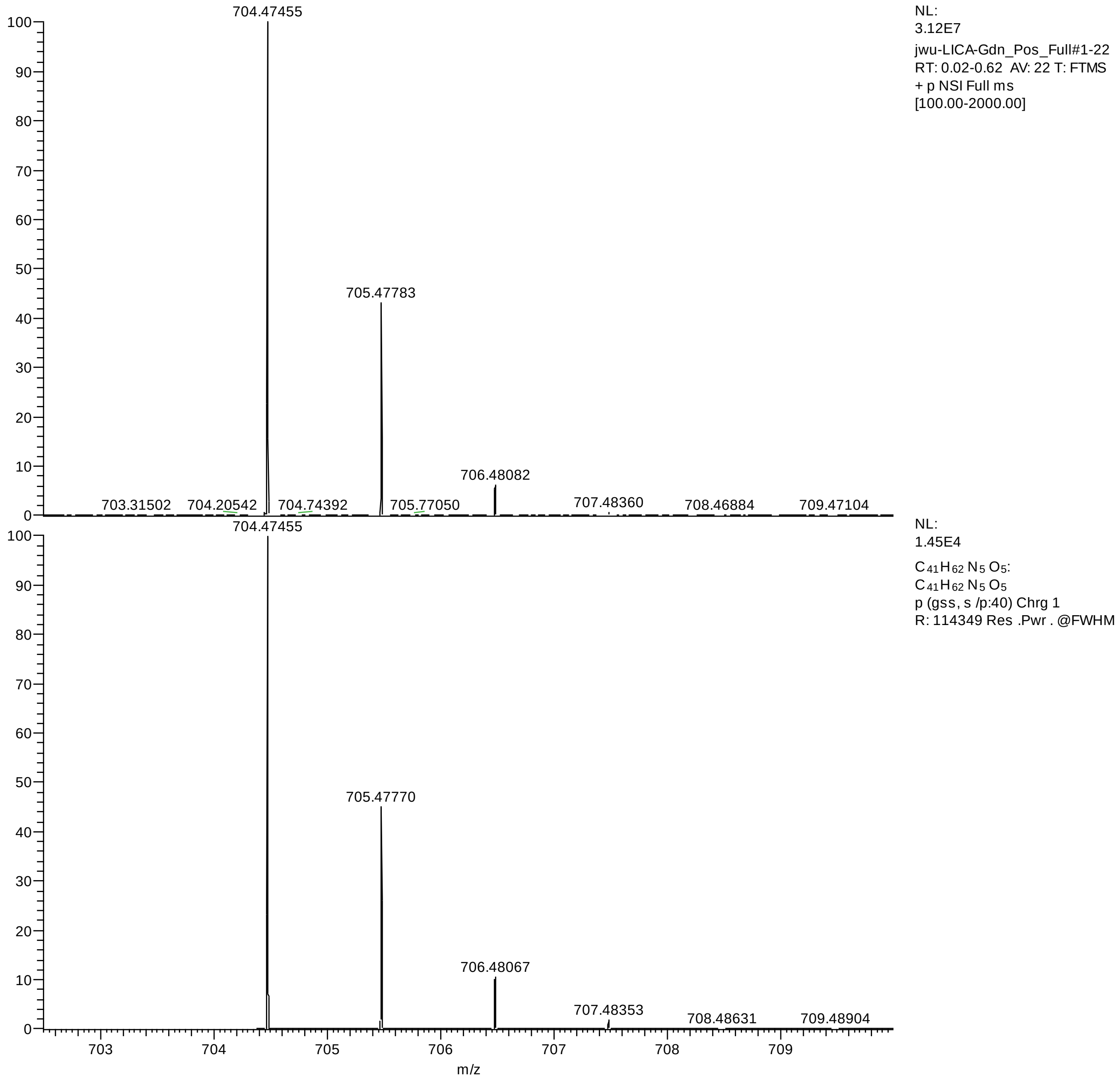

Methyl ((*R*)-4-((3*R*,5*R*,8*R*,9*S*,10*S*,13*R*,14*S*,17*R*)-3-((L-lysylglycyl)oxy)-10,13-dimethylhexadecahydro-1*H*-cyclopenta[*a*]phenanthren-17-yl)pentanoyl)-L-tryptophanate **(25)**

Full spectrum

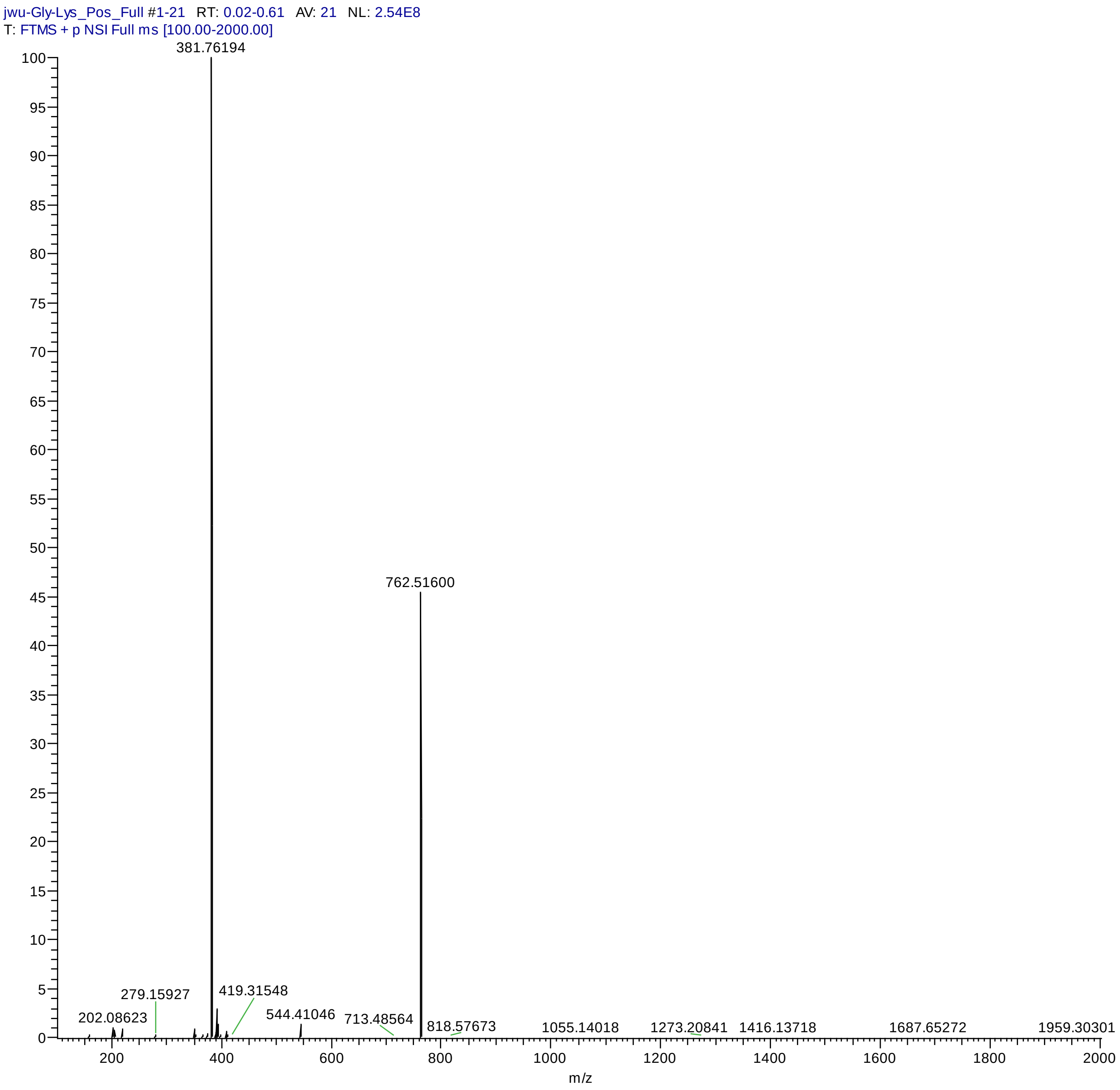

Zoomed spectrum

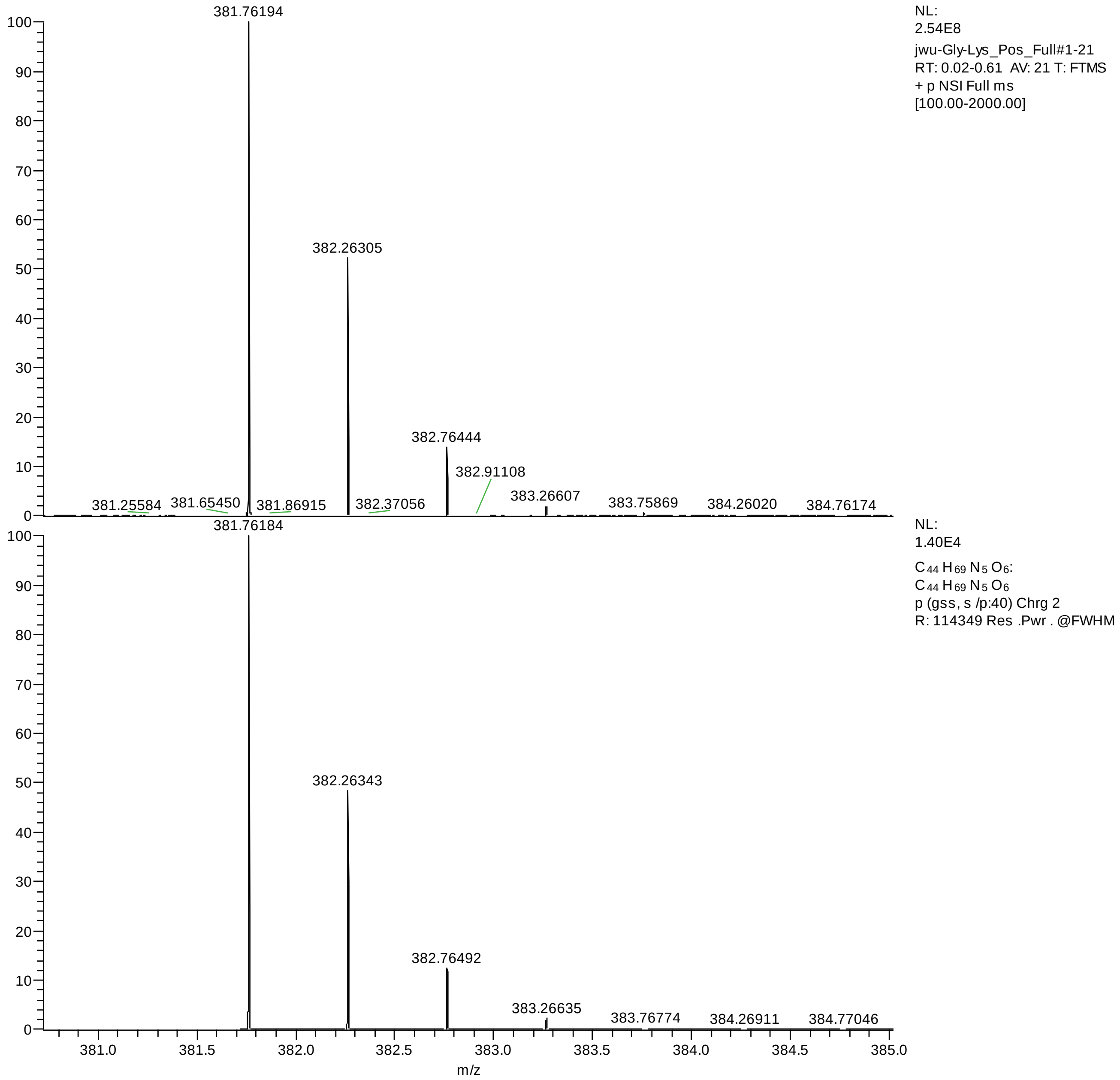

1,1',1''-(((((3*R*,5*S*,7*R*,8*R*,9*S*,10*S*,12*S*,13*R*,14*S*,17*R*)-17-((*R*)-5-(((*S*)-3-(1*H*-Indol-3-yl)-1-methoxy-1-oxopropan-2-yl)amino)-5-oxopentan-2-yl)-10,13-dimethylhexadecahydro-1*H*-cyclopenta[*a*]phenanthrene-3,7,12-triyl)tris(oxy))tris(2-oxoethane-2,1-diyl))tris(azanediyl))tris(3-methyl-1-oxobutan-2-aminium) **(27)**

Full spectrum

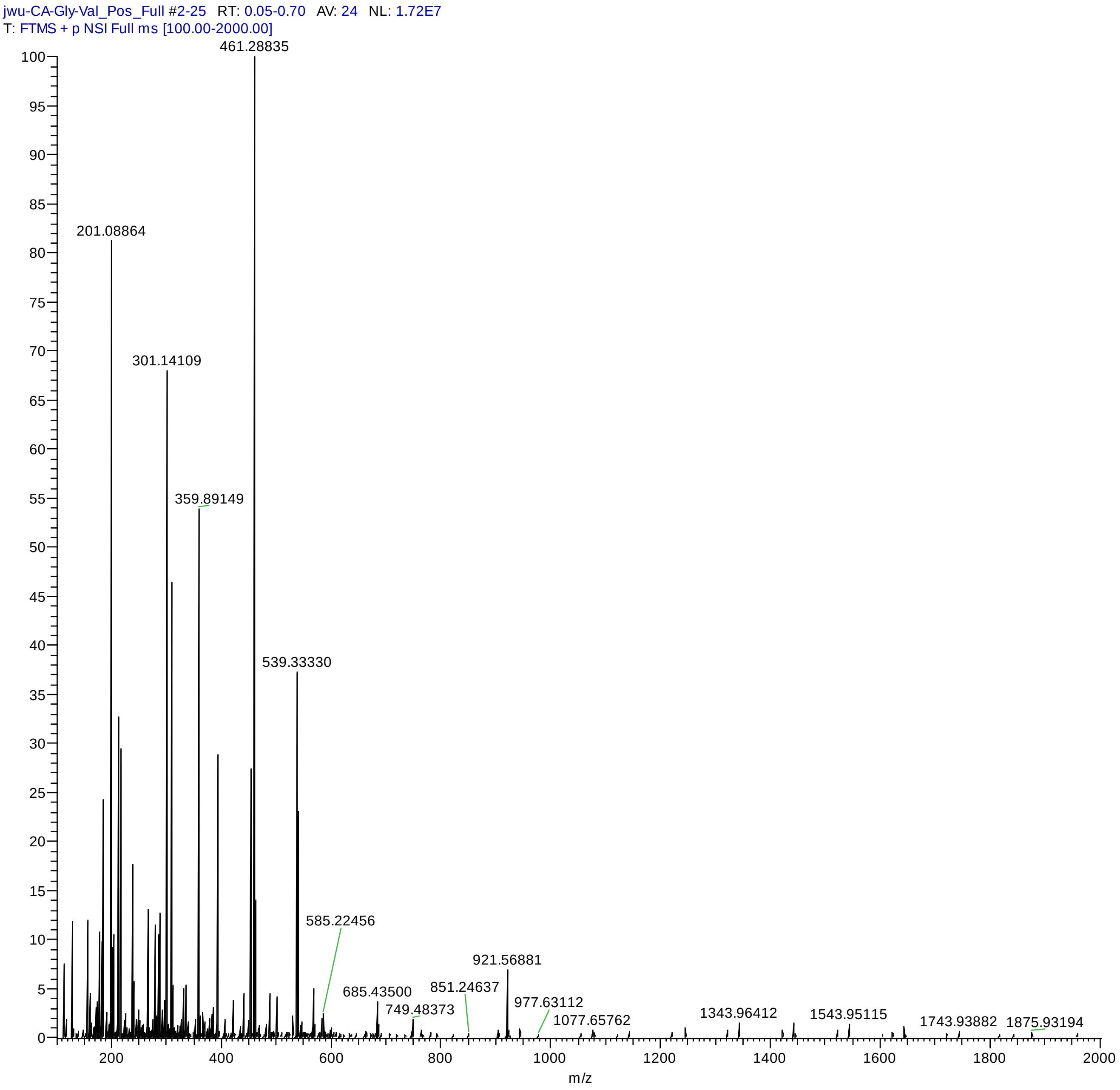

Zoomed spectrum (Top spectrum: measured mass; bottom spectrum: calculated mass)

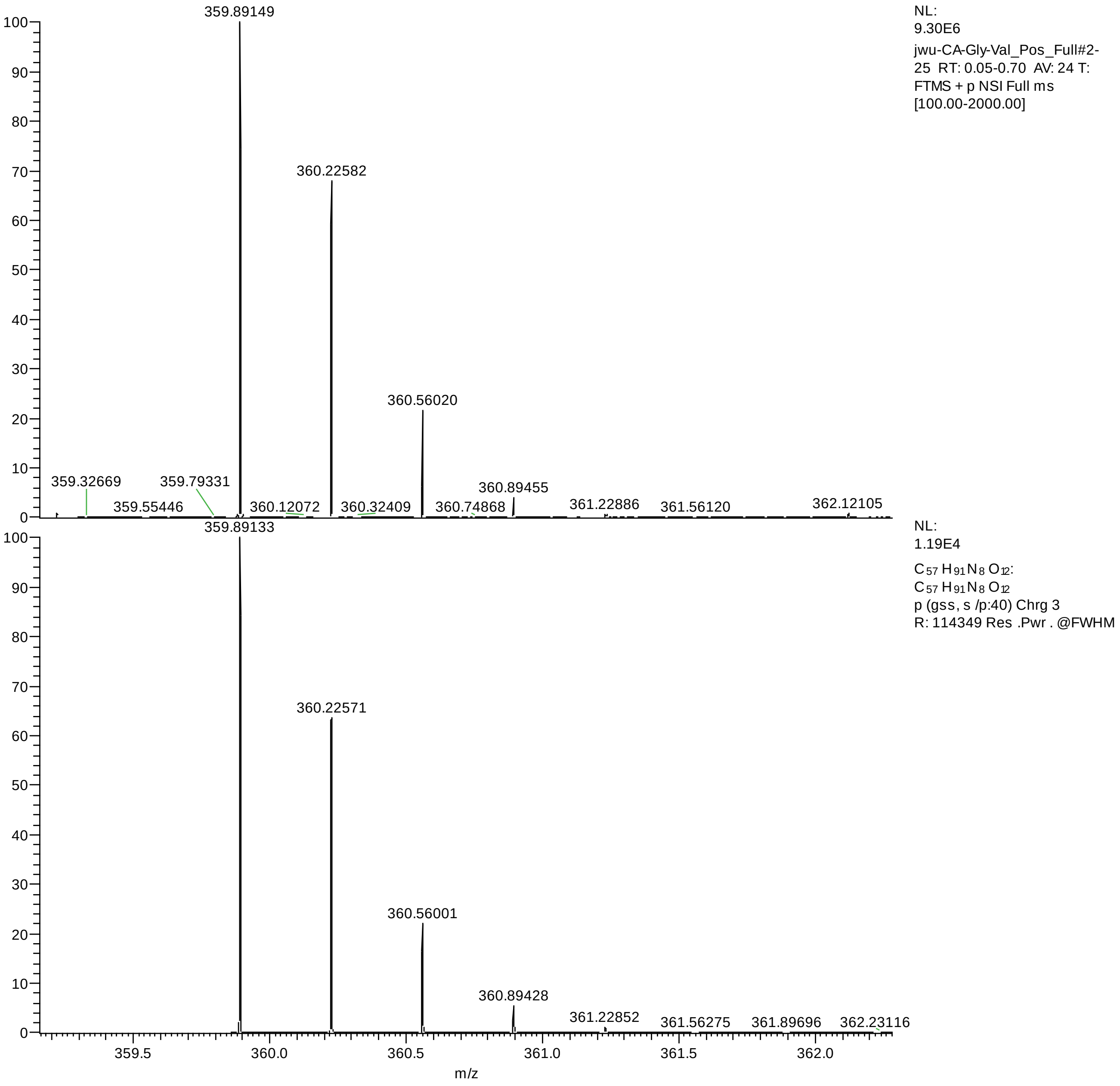

Supplement: Supplementary file 1 [file ijms-23-04623-s001.zip › ijms-1558601-supplementary.pdf]
